# A simple protocol for determining the zone axis direction from selected-area electron diffraction spot patterns of cubic materials

**DOI:** 10.1107/S1600576724004333

**Published:** 2024-06-18

**Authors:** Thomas E. Weirich

**Affiliations:** a Gemeinschaftslabor für Elektronenmikroskopie (GFE) RWTH Aachen University Ahornstrasse 55 52074Aachen Germany; Ecole National Supérieure des Mines, Saint-Etienne, France

**Keywords:** electron diffraction, zone axis spot pattern, orientation determination, cubic symmetry, ratio method

## Abstract

Using the well known *R_n_* ratio method, a protocol has been elaborated for determining the lattice direction for the 15 most common cubic zone axis spot patterns. The method makes use of the lengths of the three shortest reciprocal-lattice vectors in each pattern and the angles between them. No prior pattern calibration is required for the method to work, as the *R_n_* ratio method is based entirely on geometric relationships. In the first step the pattern is assigned to one of three possible pattern types according to the angles that are measured between the three reciprocal-lattice vectors. The lattice direction [*uvw*] and possible Bravais type(s) and Laue indices of the corresponding reflections can then be determined by using lookup tables. In addition to determining the lattice direction, this simple geometric analysis allows one to distinguish between the *P*, *I* and *F* Bravais lattices for spot patterns aligned along [013], [112], [114] and [233]. Moreover, the *F* lattice can always be uniquely identified from the [011] and [123] patterns.

## Abstract 2

Reexamination of the well known *R_n_* ratio method for indexing electron diffraction zone axis patterns of materials with cubic symmetry has revealed some unrecognized potential for zone axis direction analysis and, in some cases, unambiguous identification of the Bravais lattice. A protocol has been developed which allows the identification of experimental spot patterns for the 15 most common zone axis directions.

## 1. Introduction

Despite the yearly increase in the number of crystal structures with growing structural complexity, research into metallic alloy systems, ceramics and semiconductors is still dominated by rather small unit-cell phases with cubic or pseudo-cubic symmetry. Electron microscopists are therefore often tasked with analyzing the diffraction patterns of cubic materials with respect to their structure and/or crystal direction along the electron beam. In the author’s laboratory, this task is routinely achieved first by visual inspection and comparison of the geometry of the experimental zone axis electron diffraction patterns with standard spot patterns, followed by determination of the interplanar *d* spacings for verification. The first tables of such standard spot patterns for this purpose were possibly published by Eichen and coworkers at the Ford Scientific Laboratory, Detroit, Michigan, USA (E. Eichen, C. Laird and W. R. Bitler, *Reciprocal Lattice Diffraction Patterns for F.C.C., B.C.C., and H.C.P., and Diamond Cubic Crystal Systems*). Similar tables with standard patterns are also found in the books of Andrews *et al.* (1968 ▸), Edington (1975 ▸), Spence & Zuo (1992 ▸), Champness (2001 ▸), Fultz & Howe (2002 ▸) and Williams & Carter (2009 ▸). The recently published *Atlas of Zone Axis Spot Patterns for Cubic Lattices* contains a recompilation of the 15 most observed directions in practice (Weirich, 2024 ▸).

Another commonly used approach for indexing zone axis diffraction patterns and calculation of lattice direction is the *R_n_* ratio method. The *R_n_* ratio method is as old as X-ray crystallography itself and was first used by William Lawrence Bragg in 1913 to index the diffraction patterns of various materials with cubic symmetry (Bragg, 1913 ▸). This method takes advantage of the special geometric relations of the cubic lattice which allow one to eliminate the lattice parameter *a*, when the interplanar spacings of two reflections *A* and *B* are known,
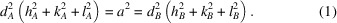
Due to the inverse relationship between the lattices in real and reciprocal space, the interplanar *d* spacings can be replaced for practical applications by the inverse distances 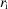 of the diffraction spots from the primary or zero beam at the center of the diffraction pattern. This leads to the key formula of the ratio method (Andrews *et al.*, 1968 ▸):
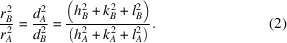
Because of its simplicity, this method has since become part of the standard teaching repertoire for the evaluation of X-ray diffraction data, particularly powder X-ray diffraction data (*e.g.* Cullity, 1978 ▸). Short introductions to the method of *R_n_* ratios for indexing zone axis spot patterns with application to electron diffraction are found in the books of Andrews *et al.* (1968 ▸) and Reimer (1993 ▸). A more detailed description of the approach including extended lists of ratios of interplanar spacings is found in the report of Nolder & Thomas (1962 ▸), later reprinted in the book of Heimendahl (1980 ▸). Despite the extensive dissemination of the *R_n_* ratio method, a search through the literature failed to find any evidence for the existence of a reliable scheme for zone axis determination based on aligned spot diffraction images. Therefore, the ratio method always needs a subsequent calculation of the angles between the indexed reflections to prove the correct zone axis assignment (Nolder & Thomas, 1962 ▸). To generalize the indexing process, the ratio method was reexamined and a protocol for lattice direction analysis was derived from visual inspection of the calculated pattern geometry.

## 2. Analysis of the geometry of cubic zone axis patterns

A systematic analysis of the 15 most frequently encountered directions in practice of the *P*, *I* and *F* Bravais lattices (Edington, 1975 ▸; Weirich, 2024 ▸) shows that there are basically only three different types of diffraction patterns that can be classified by the geometry of the three shortest reciprocal-lattice vectors.

**Figure 1:**
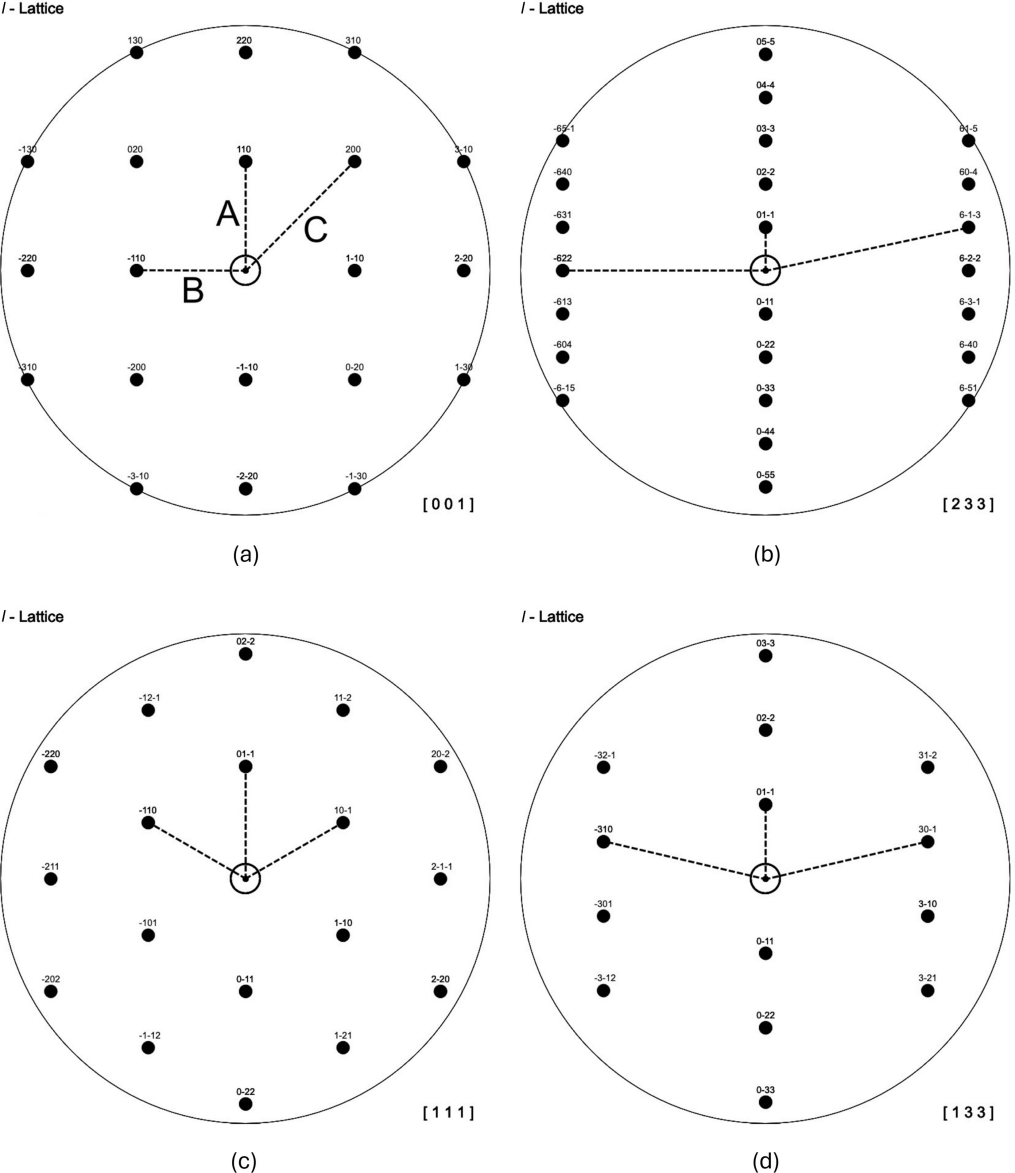
Standard spot patterns for the body-centered cubic lattice showing type I and type II patterns (patterns are not drawn to scale). The patterns of type I in (*a*) and (*b*) are characterized by a 90° angle between the shortest *A* and the second-shortest reciprocal-lattice vector *B*. The angle between the third-shortest reciprocal-lattice vector *C* and vector *A* can range from 45° for the [001] direction (*a*) to 77.96° for the [233] direction (*b*). The patterns of type II in (*c*) and (*d*) always possess two identical angles, *i.e.* ∠*A*–*B* = ∠*A*–*C*. The angles in type II patterns can range from 60° for the [111] direction in (*c*) to 77.08° for the [133] direction in (*d*).

Type I: all patterns of type I have a 90° angle between the shortest and the second-shortest reciprocal-space vector as shown by the examples in (*a*) and (*b*) in Fig. 1 ▸. The third-shortest vector and the shortest vector include an angle that can range between 45 and 77.96° for cubic lattices.

Type II: the patterns of type II are all characterized by two identical angles (and distances) between the shortest and the two second-shortest reciprocal-space vectors. In this case the angles can range between 60 and 77.08° [see patterns (*c*) and (*d*) in Fig. 1 ▸].

Type III: all type III patterns have different angles between the shortest and the next two shortest reciprocal-space vectors, which vary between 54.74 and 82.39°.

A summary of this pure geometric analysis is given in Table 1 ▸, which lists the angles and *R_n_* ratios between the three shortest reciprocal-space vectors *A*, *B* and *C* for the *P*, *I* and *F* lattices together with their corresponding lattice directions [*uvw*]. The Laue indices of the corresponding reflections *A*, *B* and *C* for the zone axes listed in Table 1 ▸ are provided in Table 2 ▸.

## 3. Protocol for indexing cubic spot patterns

**Figure 2:**
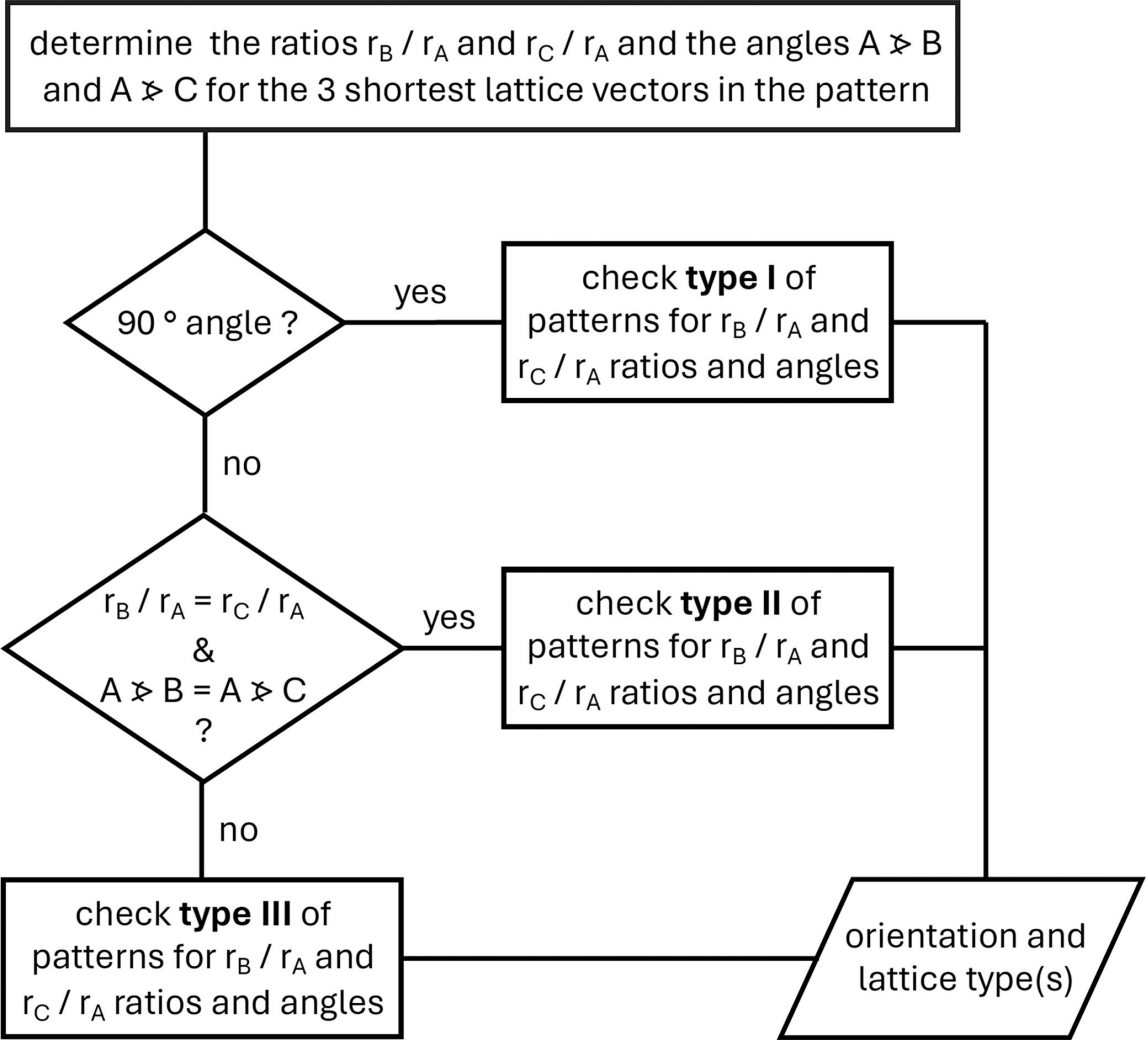
Flowchart for indexing spot patterns of cubic lattices with Table 1 ▸.

The procedure for the indexing of cubic spot patterns with the help of Table 1 ▸ is straightforward, as illustrated by the flowchart in Fig. 2 ▸. The process involves the following steps.

(i) First, the shortest reciprocal-lattice vector(s) in the pattern are identified. The corresponding reflection will be referred to throughout as *A*. Note that the square root of *N*, the sum of the squared *hkl* Laue indices *h*^2^ + *k*^2^ + *l*^2^, of this diffraction spot is always smaller than or equal to that of the next-shortest reciprocal-lattice vector *B* (see Table 3). For consistency, the direction of the *A* reciprocal-lattice vector is always drawn upwards in Fig. 1 ▸ and likewise in the *Atlas of Zone Axis Spot Patterns for Cubic Lattices* (Weirich, 2024 ▸).

(ii) Then the next-shortest reciprocal-lattice vector in the pattern is identified, which is referred to as *B*. According to the scheme used here, the diffraction spot *B* is always located on the left side of spot *A* (see Fig. 1 ▸).

(iii) Now the third-shortest reflection spot must be identified, which will be named *C*. This spot is always located on the right side of spot *A* and is obtained by vector addition according to *h*_*A*_*k*_*A*_*l*_*A*_ − *h*_*B*_*k*_*B*_*l*_*B*_ = *h*_*C*_*k*_*C*_*l*_*C*_, *e.g.* 110 − (110) = 200 as shown in Fig. 1 ▸(*a*).

(iv) For using Table 1 ▸ it is also necessary to measure the lengths of the reciprocal-lattice vectors *A*, *B* and *C* in the spot pattern (*e.g.* in pixel units), to calculate the *R_n_* ratios 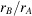 and 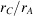 and to determine the angles between *A* and *B*, and *A* and *C*, respectively.

(v) In the next step, the type of spot pattern is identified according to the angle between *A* and *B* (see Fig. 2 ▸). An angle of 90° between *A* and *B* indicates that the spot pattern belongs to type I. If the angle between *A* and *B* is the same as that between *A* and *C* (the corresponding *R_n_* ratios will necessarily also be identical), the spot pattern belongs to type II. If none of these conditions apply, the spot pattern will be of type III (Table 1 ▸).

(vi) Using the lookup Table 1 ▸ with the determined *R_n_* ratios and angles allows one finally to identify the corresponding zone axis direction [*uvw*] and the Bravais lattice for the spot pattern. The Laue indices for *A*, *B* and *C* of the diffraction spots for a particular lattice type are given in Table 2 ▸. Ambiguous solutions, *i.e.* where the ratios and angles are compatible with different lattice types, have been marked in red in Table 1 ▸. All remaining zone axis orientations, printed in black, can be identified without ambiguity using this method. Note that there is no strict distinction made between the *F*-centered and the diamond-type lattice, for which the additional reflection conditions are (*h* + *k* + *l*) = 2*n* + 1 or (*h* + *k* + *l*) = 2*n* with *n* = even (Hammond, 2009 ▸). The reason for abandoning this distinction is that most of the kinematically forbidden reflections of the diamond lattice will appear in electron diffraction patterns under parallel electron beam illumination due to dynamical and/or secondary diffraction.

## 4. Examples

### 4.1. Analysis of a type I spot pattern from aluminium alloy AlSi1MgMn

**Figure 3:**
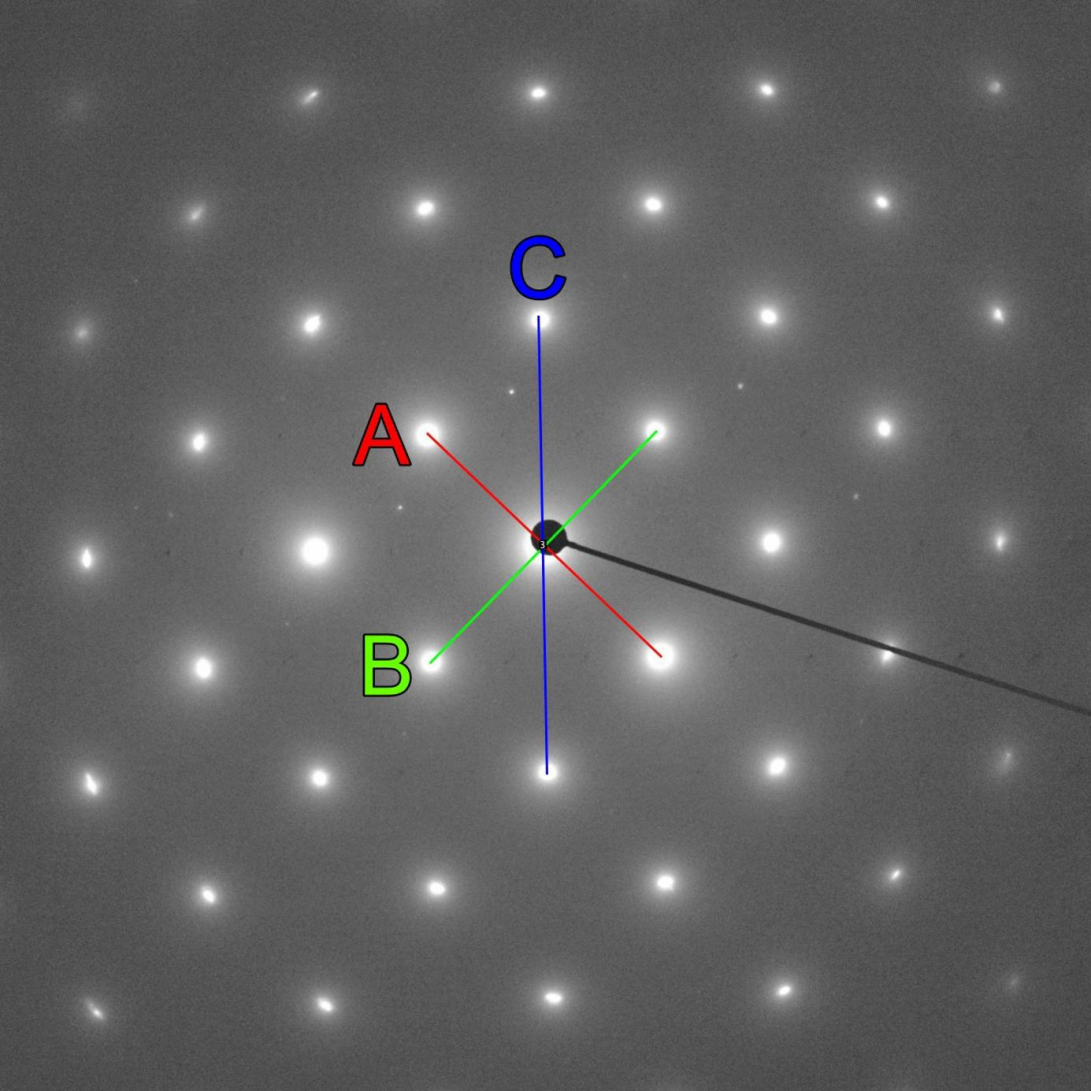
Zone axis oriented selected-area electron diffraction pattern recorded from a thin-foil sample of aluminium alloy AlSi1MgMn in a JEOL JEM-F200 at 200 kV. The uncalibrated diffraction pattern was analyzed with the line and angle tools of *ImageJ* (Schneider *et al.*, 2012 ▸) for determining the lengths of the three shortest reciprocal-lattice vectors *A*, *B* and *C* and the angles between them. For determining the distances of the reflections from the pattern center, lines were first drawn between the opposite reflections *hkl* and 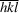 and the measured lengths were then divided by two. For the pattern shown here the thereby determined distances from the center are 209 pixels (*A*), 210 pixels (*B*) and 297 pixels (*C*), which yields the ratios 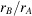 = 1.0 and 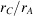 = 1.42. The measured angles are ∠*A*–*B* = 89.3° and ∠*A*–*C* = 45.3°.

The diffraction pattern in Fig. 3 ▸ was obtained from a thin-foil sample of the aluminium alloy AlSi1MgMn during an investigation on a 200 kV JEOL JEM-F200 transmission electron microscope. The diffraction pattern recorded using a OneView camera from GATAN was processed with the open-source software package *ImageJ* (Schneider *et al.*, 2012 ▸), as described in the caption of Fig. 3 ▸. The thereby determined distances of the reflections from the center are 209 pixels (*A*), 210 pixels (*B*) and 297 pixels (*C*), which yield the ratios 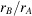 = 1.0 and 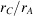 = 1.42. The measured angles between the reflections are ∠*A*–*B* = 89.3° and ∠*A*–*C* = 45.3°. Following the flowchart in Fig. 2 ▸, the diffraction pattern in Fig. 3 ▸ complies with a type I pattern since the angle ∠*A*–*B* is very close to 90°. The lookup Table 1 ▸ indicates for the experimentally determined *R_n_* ratios and the *A*–*C* angle of nearly 45° the lattice direction [001]. It should be noted that the lattice type in this case remains ambiguous since the underlying geometry for this direction is identical for all lattice types (see Weirich, 2024 ▸; pp. 4, 19, 34, 49). However, with a calibrated pattern, it would be possible to go further at this point and calculate the lattice parameter using equation (1) from the Laue indices listed in Table 2 ▸ for each solution. For the *F* lattice assumed here, the three shortest reciprocal-lattice vectors belong to the reflections with Laue indices 200 (*A*), 020 (*B*) and 220 (*C*). Alternatively, the known interplanar *d* spacings of these reflections might be used to calculate the camera constant CC for the used camera length by multiplying 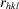 by 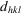.

### 4.2. Analysis of a type II spot pattern from aluminium alloy AlZn5Mg

**Figure 4:**
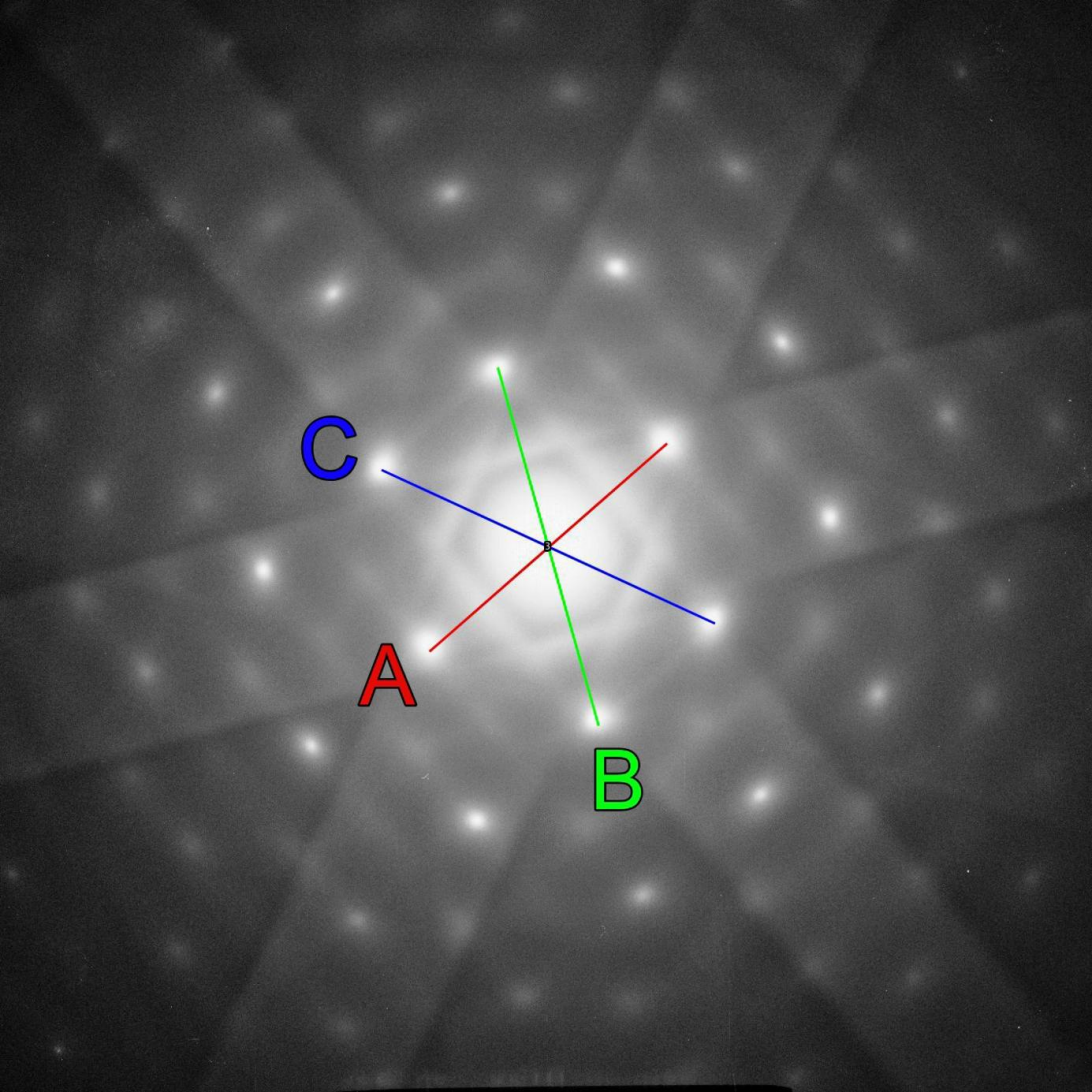
Oriented selected-area electron diffraction pattern recorded from a thin-foil sample of aluminium alloy AlZn5Mg in a 200 kV FEI Tecnai F20. The uncalibrated diffraction pattern was analyzed in the same manner as the diffraction pattern shown in Fig. 3 ▸. The determined distances of the reflections from the center are 203 pixels (*A*), 240 pixels (*B*) and 237 pixels (*C*), which yields the ratios 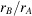 = 1.18 and 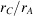 = 1.17. The measured angles are ∠*A*–*B* = 64.5° and ∠*A*–*C* = 65.9°.

As part of the investigation of the aluminium alloy AlZn5Mg in a 200 kV FEI Tecnai F20, zone axis oriented selected-area electron diffraction patterns were recorded on Kodak SO-163 film and subsequently digitized with a flatbed scanner for further analysis with *ImageJ*. The three shortest reciprocal-lattice vectors in the diffraction pattern in Fig. 4 ▸ have lengths of 203 pixels (*A*), 240 pixels (*B*) and 237 pixels (*C*). The corresponding ratios are 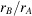 = 1.18 and 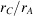 = 1.17 with the measured angles ∠*A*–*B* = 64.5° and ∠*A*–*C* = 65.9°. Following the flowchart in Fig. 2 ▸, a type I pattern can be readily ruled out as there is no angle close to 90° between the three shortest reciprocal-lattice vectors. However, since the calculated ratios 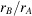 and 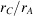 and the angles for ∠*A*–*B* and ∠*A*–*C* are practically identical within the expected experimental errors, the test for pattern type II is positive. The lookup Table 1 ▸ indicates for a pattern of type II with the averaged values of the ratios (1.175) and angles (65.2°), without ambiguity, an *F*-centered lattice with orientation [114]. With the known lattice type and direction, Table 2 ▸ readily provides the indices of the three reflections, which are 220 (*A*), 311 (*B*) and 131 (*C*). A final comparison of the experimental pattern in Fig. 4 ▸ with the [114] standard spot pattern shown in the *Atlas of Zone Axis Spot Patterns* (Weirich, 2024 ▸, p. 46) confirms the correct assignment of the zone axis direction.

### 4.3. Analysis of a type III spot pattern from aluminium alloy AlSi1MgMn

**Figure 5:**
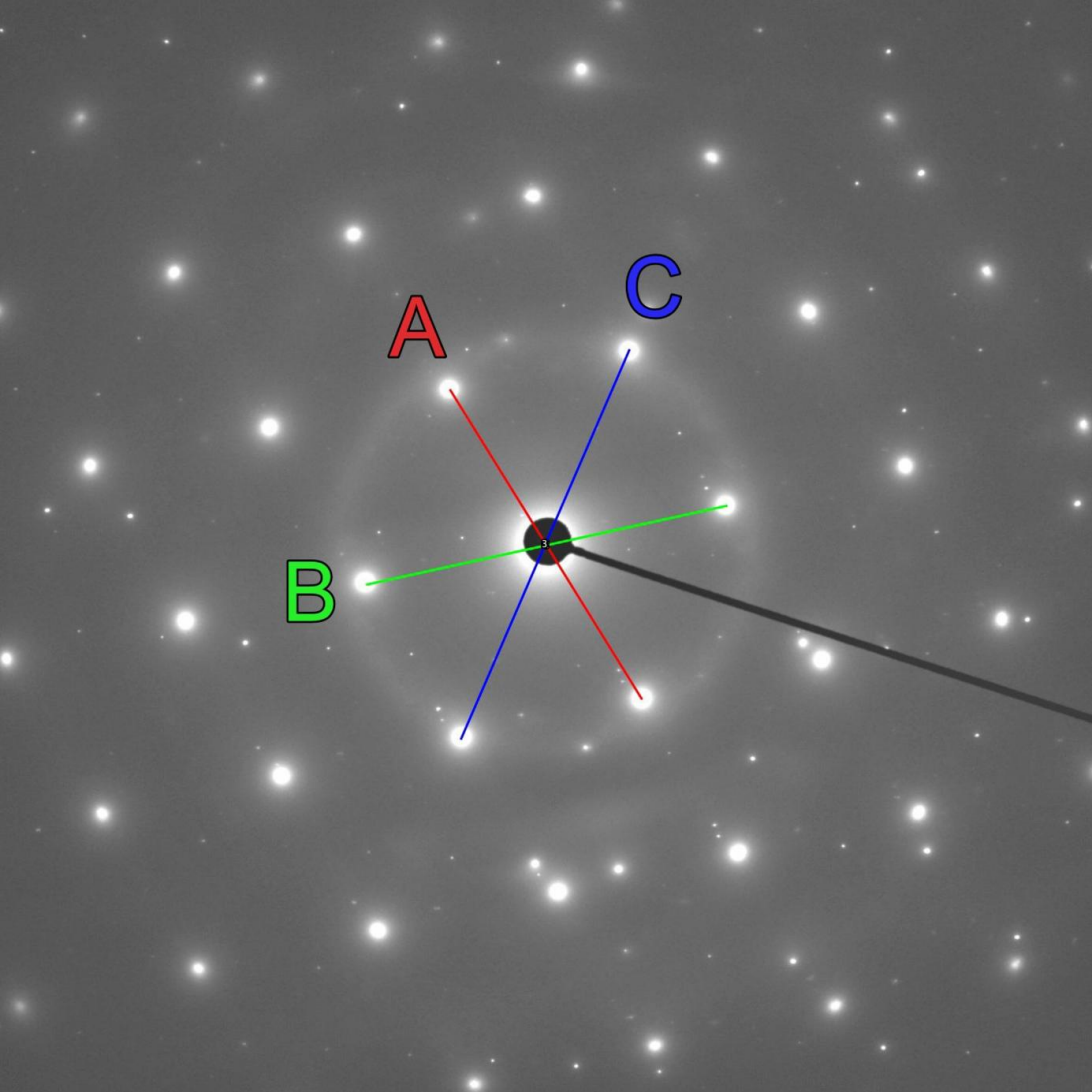
Oriented selected-area electron diffraction pattern recorded from a FIB cross section sample of aluminium alloy AlSi1MgMn in a JEOL JEM-F200 at 200 kV. The uncalibrated diffraction pattern was analyzed in the same manner as the diffraction pattern shown in Fig. 3 ▸. The determined distances of the reflections from the center are 235 pixels (*A*), 239 pixels (*B*) and 275 pixels (*C*). The thereby calculated ratios are 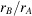 = 1.02 and 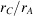 = 1.17. The measured angles are ∠*A*–*B* = 70.6° and ∠*A*–*C* = 55.2°.

The selected-area electron diffraction pattern in Fig. 5 ▸ originates from a transmission electron microscopy (TEM) investigation in a 200 kV JEOL JEM-F200 of a focused ion beam (FIB) cross section sample of deformed aluminium alloy AlSi1MgMn. Again, the basic processing of the diffraction pattern was performed as reported before. The measured distances of the reflections from the pattern center are 235 pixels (*A*), 239 pixels (*B*) and 275 pixels (*C*) with the measured angles ∠*A*–*B* = 70.6° and ∠*A*–*C* = 55.2°. The calculated ratios from the distances are 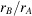 = 1.02 and 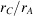 = 1.17. Following the flowchart in Fig. 2 ▸, type I and type II can easily be ruled out, as there is no 90° angle between the three reflections, nor are the two ratios and angles nearly identical. Therefore, this pattern must belong to type III. Examination of the few entries listed in Table 1 ▸ under category III shows that the ratios and angles determined are only compatible with an *F* lattice with orientation [011]. The corresponding indices of the three reflections taken from Table 2 ▸ are 111 (*A*), 111 (*B*) and 200 (*C*), which agrees with the [011] standard spot pattern in the *Atlas of Zone Axis Spot Patterns* (Weirich, 2024 ▸, p. 35).

## 5. Discussion and conclusion

The present study reexamined the long-established ratio method for indexing the 15 most commonly found cubic zone axis spot patterns in practice and developed a reliable protocol for determining the lattice direction. The method proposed here requires only analysis of the ratios of the three shortest reciprocal-lattice vectors and the angles between them. Hence no prior calibration of the diffraction pattern is required as is typical for the approach with *R_n_* ratios. On the basis of the measured angles, each pattern can be assigned to one of three different types of patterns in the first step. Using a lookup table with the calculated experimental *R_n_* ratios and angles, the lattice direction [*uvw*] and the possible Bravais lattice type can readily be identified. Once the lattice type and crystal direction along the electron beam are known, the corresponding indices of the three basic reflections can be taken from another lookup table.

**Figure 6:**
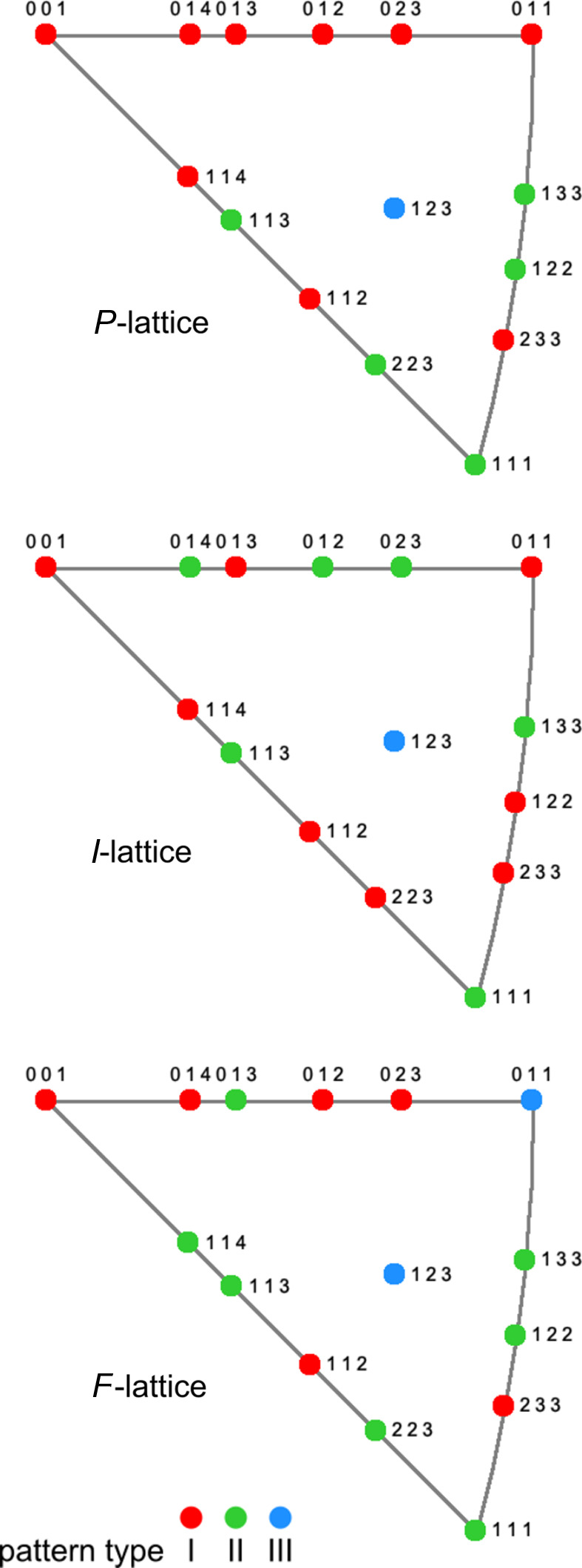
IPF plots for the *I*, *P* and *F* lattices with the pattern type for the analyzed lattice directions [*uvw*] by color (data taken from Table 1 ▸). The [*uvw*] indices within the IPF triangle are subject to the following rules: [0*kl*] between [001] and [011], [*hhl*] between [001] and [111], and [*hkk*] between [111] and [011].

As the inverse pole figure (IPF) plots in Fig. 6 ▸ show, the pattern type sequence along the large circles between [001] and the other two corners is different for the *P*, *I* and *F* lattices. While for the *P* lattice the four directions between [001] and [111] belong to pattern type I only, the pattern type sequence for the *I* and *F* lattices is mixed and reversed for these directions. Therefore, these IPF plots may serve to quickly resolve ambiguous results through analysis of another pattern from the same sample region, which is easily accessible by tilting the crystal, or by analyzing a pattern that is from the same material in the sample.

A closer analysis of lookup Table 1 ▸ reveals that type I spot patterns allow one to distinguish ten different combinations of lattice type and direction. For type II and type III only two patterns each can be distinguished and uniquely identified. A summary of all uniquely determinable combinations of lattice type and direction is presented in Table 3 ▸. The latter shows that *P*, *I* and *F* lattices can be uniquely identified by geometric analysis alone when spot patterns with directions [013], [112], [114] and [233] are used. Furthermore, the *F* lattice can be uniquely identified from the [011] and [123] directions.

The analysis of the pattern geometry outlined here has demonstrated that the well known *R_n_* ratio method bears some hidden potential for rapid determination of lattice direction and, in some cases, even allows unambiguous identification of the Bravais lattice, as in the case of the frequently encountered [011] diffraction pattern of the face-centered cubic lattice. Therefore, the proposed method could be highly beneficial for all electron microscopists who work with cubic materials.

## Figures and Tables

**Table 1:** Result of the geometric analysis of the 15 standard spot patterns The analysis is based on the three shortest distances *A*, *B* and *C*, where *A* ≤ *B* ≤ *C*. The values for 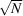 = (*h*^2^ + *k*^2^ + *l*^2^)^1/2^ were calculated with the corresponding indices from Table 2 ▸. Directions marked with a star (*) should be checked for a possible diamond-type lattice with the same ratios and angles (see Weirich, 2024 ▸).

Type	∠*A*–*B* (°)	*r_B_*/*r_A_*	∠*A*–*C* (°)	*r_C_* /*r_A_*	Lattice type	[*uvw*]
I	90.0	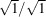 = 1.0	45.0	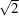 / 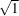 = 1.414	*P*	001*
		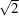 / 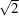 = 1.0		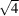 / 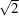 = 1.414	*I*	
		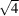 / 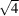 = 1.0		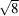 / 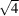 = 1.414	*F*	
		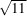 / 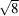 = 1.173	49.54	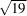 / 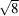 = 1.541	*F*	233*
		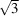 / 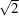 = 1.225	50.77	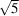 / 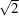 = 1.581	*P*	112
		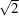 / 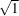 = 1.414	54.74	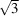 / 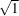 = 1.732	*P*	011
		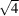 / 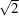 = 1.414		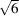 / 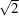 = 1.732	I	
		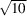 / 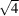 = 1.581	57.69	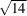 / 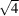 = 1.871	*I*	013
		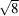 / 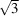 = 1.633	58.52	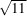 / 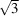 = 1.915	*F*	112*
		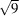 / 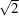 = 2.121	64.76	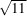 / 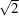 = 2.345	*P*	114
		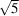 / 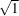 = 2.236	65.91	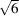 / 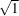 = 2.449	*P*	012
		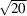 / 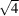 = 2.236		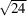 / 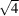 = 2.449	*F*	
		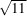 / 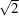 = 2.345	66.91	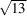 / 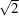 = 2.55	*P*	233
		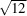 / 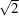 = 2.449	67.79	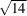 / 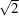 = 2.646	*I*	112
		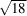 / 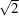 = 3.0	71.57	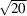 / 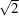 = 3.162	*I*	122*
		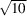 / 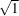 = 3.162	72.45	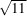 / 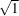 = 3.317	*P*	013
		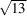 / 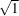 = 3.606	74.5	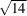 / 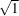 = 3.742	*P*	023
		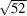 / 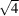 = 3.606		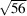 / 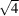 = 3.742	*F*	
		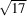 / 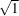 = 4.123	76.37	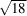 / 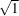 = 4.243	*P*	014
		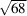 / 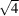 = 4.123		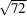 / 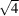 = 4.243	*I*	
		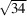 / 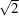 = 4.123		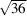 / 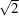 = 4.243	*F*	223*
		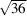 / 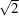 = 4.243	76.74	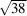 / 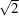 = 4.359	*I*	114
		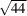 / 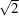 = 4.69	77.96	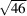 / 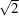 = 4.796	*I*	233

II	60.0	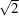 / 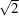 = 1.0	60.0	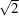 / 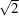 = 1.0	*P*	111*
		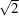 / 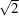 = 1.0		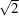 / 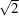 = 1.0	*I*	
		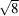 / 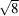 = 1.0		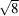 / 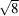 = 1.0	*F*	
	64.76	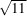 / 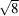 = 1.173	64.76	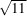 / 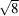 = 1.173	*F*	114*
	65.91	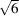 / 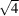 = 1.225	65.91	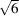 / 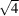 = 1.225	*I*	012*
	71.57	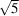 / 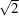 = 1.581	71.57	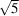 / 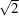 = 1.581	*P*	122
		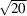 / 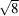 = 1.581		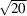 / 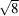 = 1.581	*F*	
	72.45	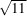 / 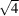 = 1.658	72.45	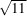 / 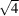 = 1.658	*F*	013*
	73.22	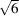 / 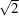 = 1.732	73.22	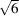 / 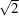 = 1.732	*P*	113*
		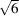 / 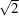 = 1.732		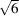 / 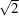 = 1.732	*I*	
		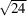 / 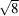 = 1.732		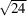 / 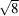 = 1.732	*F*	
	74.5	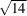 / 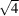 = 1.871	74.5	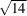 / 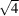 = 1.871	*I*	023*
	76.37	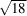 / 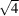 = 2.121	76.37	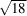 / 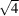 = 2.121	*I*	014*
		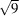 / 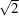 = 2.121		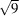 / 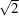 = 2.121	*P*	223
		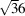 / 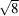 = 2.121		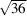 / 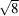 = 2.121	*F*	
	77.08	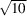 / 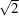 = 2.236	77.08	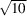 / 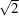 = 2.236	*P*	133*
		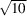 / 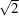 = 2.236		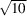 / 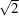 = 2.236	*I*	
		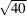 / 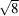 = 2.236		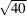 / 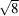 = 2.236	*F*	

III	61.87	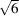 / 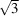 = 1.414	75.04	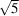 / 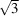 = 1.291	*P*	123
	75.04	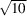 / 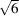 = 1.291	61.87	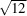 / 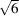 = 1.414	*I*	123
	70.53	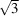 / 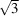 = 1.0	54.74	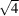 / 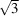 = 1.155	*F*	011*
	82.39	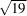 / 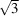 = 2.517	75.04	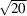 / 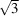 = 2.582	*F*	123*

**Table 2:** Laue indices *hkl* of the three shortest reciprocal-lattice vectors *A*, *B* and *C* for the zone axes [*uvw*] listed in Table 1 ▸

[*uvw*]	Lattice type	*hkl* *A*	*hkl* *B*	*hkl* *C*
001	*P*	100	010	110
	*I*	110	110	200
	*F*	200	020	220
011	*P*	100	011	111
	*I*	011	200	211
	*F*	111	111	200
111	*P*	011	110	101
	*I*	011	110	101
	*F*	202	022	220
012	*P*	100	021	121
	*I*	200	121	121
	*F*	200	042	242
112	*P*	110	111	021
	*I*	110	222	132
	*F*	111	220	311
122	*P*	011	210	201
	*I*	011	411	402
	*F*	022	420	402
013	*P*	100	031	131
	*I*	200	031	231
	*F*	200	131	131
113	*P*	110	211	121
	*I*	110	211	121
	*F*	220	422	242
023	*P*	100	032	132
	*I*	200	132	132
	*F*	200	064	264
123	*P*	111	121	210
	*I*	121	301	222
	*F*	111	331	420
014	*P*	100	041	141
	*I*	200	141	141
	*F*	200	082	282
223	*P*	110	212	122
	*I*	110	334	244
	*F*	220	424	244
114	*P*	110	221	131
	*I*	110	442	352
	*F*	220	311	131
133	*P*	011	310	301
	*I*	011	310	301
	*F*	022	620	602
233	*P*	011	311	302
	*I*	011	622	613
	*F*	022	311	313

**Table 3:** Bravais lattices and orientations uniquely determinable by their lattice geometry, sorted by pattern type

Pattern type I		Pattern type II		Pattern type III	
Bravais lattice	[*uvw*]	Bravais lattice	[*uvw*]	Bravais lattice	[*uvw*]
*P*	013	*F*	013	*F*	011
*I*	013	*F*	114	*F*	123
*P*	112				
*I*	112				
*F*	112				
*P*	114				
*I*	114				
*P*	233				
*F*	233				
*I*	233				
