# A simple protocol for determining the zone axis direction from selected-area electron diffraction spot patterns of cubic materials. Addendum. Comprehensive tables for pattern reindexing

**DOI:** 10.1107/S1600576726003031

**Published:** 2026-04-30

**Authors:** Thomas E. Weirich

**Affiliations:** a Gemeinschaftslabor für Elektronenmikroskopie (GFE) RWTH Aachen University Ahornstrasse 55 52074Aachen Germany; Universität Duisburg-Essen, Germany

**Keywords:** electron diffraction, zone axis spot patterns, cubic symmetry, reindexing

## Abstract

A set of 45 reindexing tables has been calculated for the 15 most common zone axis diffraction patterns of cubic lattices (*P*, *I* and *F* types). These tables cover all possible alternative lattice orientations, providing a systematic and efficient method of reindexing *hkl* reflections. They complement and extend the *Atlas of Zone Axis Spot Patterns for Cubic Lattices* (Weirich, 2024) and the table of standard settings provided by Weirich [*J. Appl. Cryst.* (2024), **57**, 1263–1269].

## Abstract 2

This article presents 45 symmetry-based tables which enable the reindexing of the 15 most common diffraction patterns of cubic materials. These tables are a supplement to the table of standard settings provided by Weirich [*J. Appl.Cryst.* (2024), **57**, 1263–1269].

## 1. Introduction

In a previous article (Weirich, 2024*a* ▸), a protocol for identifying lattice directions for the 15 most common cubic zone axis electron diffraction patterns was introduced. This was accomplished using the *R_n_* ratio method, which utilized the three shortest reciprocal-lattice vectors and the angles between them. As the *Atlas of Zone Axis Spot Patterns for Cubic Lattices* (Weirich, 2024*b* ▸ ▸) and the table in the recent work only provide one standard orientation as solution, there may be limitations for practical applications if a different orientation is required. To address this issue, tables were compiled for the 15 above-mentioned orientations, which list all alternative orientations together with the Laue indices for the three base vectors.

## 2. Method

The *hkl* Laue indices of the three basis vectors for the alternative orientations of the 15 zone axis directions shown in Fig. 1 ▸ were calculated by means of the 48 inverse matrices *M*^−1^ (see supplementary material, pp. 3–8) of the point symmetry operations for the cubic crystal system (Wondratschek & Aroyo, 2016 ▸; Borchardt-Ott & Sowa, 2018 ▸) using equation (1):
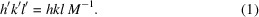
Each row in the obtained lists (see supplementary material, pp. 9–50) contains the alternative zone axis direction 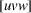, the Laue indices of the three base vectors *A*, *B* and *C*, and the reference to the matrix equation used for the calculation. As two symmetry operations always yield the same orientation for the 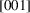 and 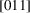 zone axes, these tables have been shortened accordingly to improve readability.

## 3. Example

Fig. 2 ▸ illustrates the procedure for reindexing and shows the six possible variants for indexing a 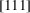 face-centered cubic (f.c.c.) lattice. The pattern labeled M1 corresponds to standard indexing (Weirich, 2024*a* ▸; Weirich, 2024*b* ▸), with *A* = 202 (pointing to north), *B* = 022 and *C* = 220 (see Table 9 on p. 14 of the supplementary material). For tracking the movement of the initial indices during reindexing, the diffraction spots *A* and *B* have been marked with a star. Note that the corresponding matrix equation (M1 on p. 3 of the supplementary material) is the identity matrix, which does not change the *hkl* indices and refers therefore always to the initial (standard) indices before the transformation. An alternative setting for the same 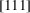 zone axis can now be obtained by searching the table for all entries with the same indices and replacing the initial indices of *A*, *B* and *C* of the standard setting with those from the corresponding row. The other five patterns in Fig. 2 ▸ were obtained in this way, with the condition that the spots relating to *A* were plotted so that they always point towards north. The matrix equations used to calculate the *hkl* Laue indices for a reindexed pattern are shown in the top-left corner of each pattern.

## 4. Summary

A comprehensive set of 45 tables for reindexing the 15 most common zone axis diffraction patterns of cubic lattices have been calculated for the *P*, *I* and *F* Bravais lattices by means of the inverse matrices of the point symmetry operations for the cubic crystal system. This new set of tables is a valuable supplementary resource to the *Atlas of Zone Axis Spot Patterns for Cubic Lattices* (Weirich, 2024*b* ▸) and the table presented by Weirich (2024*a* ▸), since they facilitate the reindexing of the *hkl* indices of standard patterns in a streamlined manner.

## Supplementary Material

Supporting Material. DOI: 10.1107/S1600576726003031/uz5031sup1.pdf

## Figures and Tables

**Figure 1:**
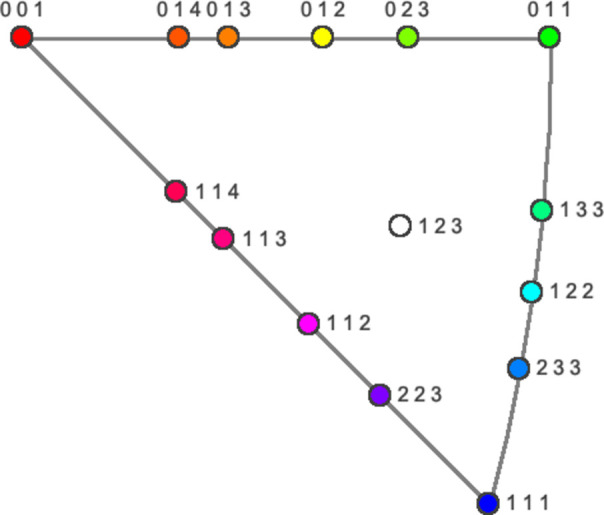
The 15 (standard) zone axis directions covered by the earlier paper (Weirich, 2024*a* ▸) and the *Atlas of Zone Axis Spot Patterns for Cubic Lattices* (Weirich, 2024*b* ▸) are shown within the standard stereographic triangle of the cubic system. All alternative orientations and corresponding *hkl* Laue indices for their pattern base vectors have been calculated for the cubic *P*, *I* and *F* lattices and compiled in tables (see supplementary material, pp. 9–50.

**Figure 2:**
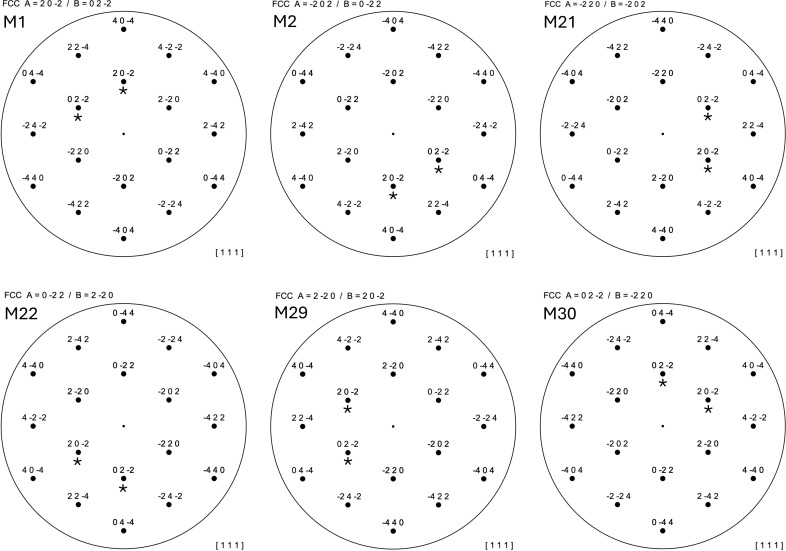
The six possible variants for indexing a 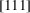 f.c.c. lattice are obtained from Table 9 on p. 14 of the supplementary material. The corresponding matrix equation *M* (see supplementary material, pp. 3–8) to obtain the indices in the reindexed pattern is shown in the top-left corner of each pattern. For comparison, each pattern has been drawn so that the reciprocal lattice vector *A* points to the north, and the spot indices *A* and *B* of the standard setting are labeled with stars.
