# Supplementary material for: A simple protocol for determining the zone axis direction from selected-area electron diffraction spot patterns of cubic materials. Addendum. Comprehensive tables for pattern reindexing
Source: J Appl Crystallogr. 2026 Apr 30;59(Pt 3):985–7. doi: 10.1107/S1600576726003031 (PMC13224787; doi:10.1107/S1600576726003031)
Supplement: Supplementary file 1 [file j-59-00985-sup1.pdf]

JOURNAL OF APPLIED CRYSTALLOGRAPHY

Volume 59 (2026)

Supporting information for article:

A simple protocol for determining the zone axis direction from selected-area electron diffraction spot patterns of cubic materials. Addendum. Comprehensive tables for pattern reindexing

Thomas E. Weirich

https://doi.org/10.1107/S1600576726003031

Supplementary Material for Manuscript

A simple protocol for determining the zone axis direction from selected-area electron diffraction spot patterns of cubic materials. Addendum. Comprehensive tables for pattern reindexing

Thomas E. Weirich *

Gemeinschaftslabor für Elektronenmikroskopie (GFE), RWTH Aachen University, Ahornstrasse 55, 52074 Aachen, Germany.

* Correspondence e-mail: weirich@gfe.rwth-aachen.de

IUCr No.: 5999
ORCID: 0000-0001-5539-0534

This compilation contains 45 tables that enable the reindexing of the *hkl* Laue indices of the standard zone axis spot patterns for the cubic P, I, and F lattices, which are given in the *Atlas of Zone Axis Spot Patterns for Cubic Lattices* [1], to any other possible lattice orientation. This compilation also supplements the tables given in [2] for determining the zone axis orientation using the ratio method. For each of the 15 orientations of the three Bravais lattices, a separate table is provided with the corresponding *hkl* Laue indices for the three shortest reciprocal lattice vectors *A*, *B*, and *C* for the alternate setting (for notation, see [2]).

The *hkl* indices for the different orientations were calculated by means of the 48 inverse matrices (see, pages 3 - 8) of the point symmetry operations for the cubic crystal system [3, 4]. The corresponding equation used to calculate the *hkl* indices is given at the end of each table entry. For the [001] and [011] zone axes, two symmetry operations always give the same orientation. These tables have been shortened accordingly to improve readability.

[1] Weirich, T. E. (2024a). *Atlas of Zone Axis Spot Patterns for Cubic Lattices.* RWTH Publications, RWTH Aachen University, Aachen, Germany. DOI: 10.18154/RWTH-2024-02030

[2] Weirich, T. E. (2024b). *J. Appl. Cryst.* 57, 1263 – 1269. DOI 10.1107/S1600576724004333

[3] Wondratschek, H., Aroyo, M. I. (2016). *Matrix description of symmetry operations.* In: International Tables for Crystallography Volume A (Space-group symmetry). Ed. M. I. Aroyo, International Union of Crystallography and Wiley, Section 1.2.2, 13 – 19.

[4] Borchardt-Ott, W., Sowa, H. (2018). *Kristallographie: Eine Einführung für Studierende der Naturwissenschaften.* Springer-Verlag Berlin Heidelberg. DOI 10.1007/978-3-662-56816-3

List of Tables

1 P - lattice [0 0 1] . . . 9
2 I - lattice [0 0 1] . . . 9
3 F - lattice [0 0 1] . . . 10
4 P - lattice [0 1 1] . . . 10
5 I - lattice [0 1 1] . . . 11
6 F - lattice [0 1 1] . . . 11
7 P - lattice [1 1 1] . . . 12
8 I - lattice [1 1 1] . . . 13
9 F - lattice [1 1 1] . . . 14
10 P - lattice [0 1 2] . . . 15
11 I - lattice [0 1 2] . . . 16
12 F - lattice [0 1 2] . . . 17
13 P - lattice [1 1 2] . . . 18
14 I - lattice [1 1 2] . . . 19
15 F - lattice [1 1 2] . . . 20
16 P - lattice [1 2 2] . . . 21
17 I - lattice [1 2 2] . . . 22
18 F - lattice [1 2 2] . . . 23
19 P - lattice [0 1 3] . . . 24
20 I - lattice [0 1 3] . . . 25
21 F - lattice [0 1 3] . . . 26
22 P - lattice [1 1 3] . . . 27
23 I - lattice [1 1 3] . . . 28
24 F - lattice [1 1 3] . . . 29
25 P - lattice [0 2 3] . . . 30
26 I - lattice [0 2 3] . . . 31
27 F - lattice [0 2 3] . . . 32
28 P - lattice [1 2 3] . . . 33
29 I - lattice [1 2 3] . . . 34
30 F - lattice [1 2 3] . . . 35
31 P - lattice [0 1 4] . . . 36
32 I - lattice [0 1 4] . . . 37
33 F - lattice [0 1 4] . . . 38
34 P - lattice [2 2 3] . . . 39
35 I - lattice [2 2 3] . . . 40
36 F - lattice [2 2 3] . . . 41
37 P - lattice [1 1 4] . . . 42
38 I - lattice [1 1 4] . . . 43
39 F - lattice [1 1 4] . . . 44
40 P - lattice [1 3 3] . . . 45
41 I - lattice [1 3 3] . . . 46
42 F - lattice [1 3 3] . . . 47
43 P - lattice [2 3 3] . . . 48
44 I - lattice [2 3 3] . . . 49
45 F - lattice [2 3 3] . . . 50

$$\begin{matrix} h & k & l \end{matrix} = \begin{matrix} h & k & l \end{matrix} \cdot \begin{pmatrix} 1 & 0 & 0 \\ 0 & 1 & 0 \\ 0 & 0 & 1 \end{pmatrix} \tag{M1}$$

$$\begin{matrix} -h & -k & -l \end{matrix} = \begin{matrix} h & k & l \end{matrix} \cdot \begin{pmatrix} -1 & 0 & 0 \\ 0 & -1 & 0 \\ 0 & 0 & -1 \end{pmatrix} \tag{M2}$$

$$\begin{matrix} h & -k & -l \end{matrix} = \begin{matrix} h & k & l \end{matrix} \cdot \begin{pmatrix} 1 & 0 & 0 \\ 0 & -1 & 0 \\ 0 & 0 & -1 \end{pmatrix} \tag{M3}$$

$$\begin{matrix} -h & k & -l \end{matrix} = \begin{matrix} h & k & l \end{matrix} \cdot \begin{pmatrix} -1 & 0 & 0 \\ 0 & 1 & 0 \\ 0 & 0 & -1 \end{pmatrix} \tag{M4}$$

$$\begin{matrix} -h & -k & l \end{matrix} = \begin{matrix} h & k & l \end{matrix} \cdot \begin{pmatrix} -1 & 0 & 0 \\ 0 & -1 & 0 \\ 0 & 0 & 1 \end{pmatrix} \tag{M5}$$

$$\begin{matrix} k & h & -l \end{matrix} = \begin{matrix} h & k & l \end{matrix} \cdot \begin{pmatrix} 0 & 1 & 0 \\ 1 & 0 & 0 \\ 0 & 0 & -1 \end{pmatrix} \tag{M6}$$

$$\begin{matrix} -k & -h & -l \end{matrix} = \begin{matrix} h & k & l \end{matrix} \cdot \begin{pmatrix} 0 & -1 & 0 \\ -1 & 0 & 0 \\ 0 & 0 & -1 \end{pmatrix} \tag{M7}$$

$$\begin{matrix} l & -k & h \end{matrix} = \begin{matrix} h & k & l \end{matrix} \cdot \begin{pmatrix} 0 & 0 & 1 \\ 0 & -1 & 0 \\ 1 & 0 & 0 \end{pmatrix} \tag{M8}$$

$$\begin{matrix} -l & -k & -h \end{matrix} = \begin{matrix} h & k & l \end{matrix} \cdot \begin{pmatrix} 0 & 0 & -1 \\ 0 & -1 & 0 \\ -1 & 0 & 0 \end{pmatrix} \tag{M9}$$

$$\begin{pmatrix} -h & l & k \end{pmatrix} = \begin{pmatrix} h & k & l \end{pmatrix} \cdot \begin{pmatrix} -1 & 0 & 0 \\ 0 & 0 & 1 \\ 0 & 1 & 0 \end{pmatrix} \tag{M10}$$

$$\begin{pmatrix} -h & -l & -k \end{pmatrix} = \begin{pmatrix} h & k & l \end{pmatrix} \cdot \begin{pmatrix} -1 & 0 & 0 \\ 0 & 0 & -1 \\ 0 & -1 & 0 \end{pmatrix} \tag{M11}$$

$$\begin{pmatrix} -h & k & l \end{pmatrix} = \begin{pmatrix} h & k & l \end{pmatrix} \cdot \begin{pmatrix} -1 & 0 & 0 \\ 0 & 1 & 0 \\ 0 & 0 & 1 \end{pmatrix} \tag{M12}$$

$$\begin{pmatrix} h & -k & l \end{pmatrix} = \begin{pmatrix} h & k & l \end{pmatrix} \cdot \begin{pmatrix} 1 & 0 & 0 \\ 0 & -1 & 0 \\ 0 & 0 & 1 \end{pmatrix} \tag{M13}$$

$$\begin{pmatrix} h & k & -l \end{pmatrix} = \begin{pmatrix} h & k & l \end{pmatrix} \cdot \begin{pmatrix} 1 & 0 & 0 \\ 0 & 1 & 0 \\ 0 & 0 & -1 \end{pmatrix} \tag{M14}$$

$$\begin{pmatrix} -k & -h & l \end{pmatrix} = \begin{pmatrix} h & k & l \end{pmatrix} \cdot \begin{pmatrix} 0 & -1 & 0 \\ -1 & 0 & 0 \\ 0 & 0 & 1 \end{pmatrix} \tag{M15}$$

$$\begin{pmatrix} k & h & l \end{pmatrix} = \begin{pmatrix} h & k & l \end{pmatrix} \cdot \begin{pmatrix} 0 & 1 & 0 \\ 1 & 0 & 0 \\ 0 & 0 & 1 \end{pmatrix} \tag{M16}$$

$$\begin{pmatrix} -l & k & -h \end{pmatrix} = \begin{pmatrix} h & k & l \end{pmatrix} \cdot \begin{pmatrix} 0 & 0 & -1 \\ 0 & 1 & 0 \\ -1 & 0 & 0 \end{pmatrix} \tag{M17}$$

$$\begin{pmatrix} l & k & h \end{pmatrix} = \begin{pmatrix} h & k & l \end{pmatrix} \cdot \begin{pmatrix} 0 & 0 & 1 \\ 0 & 1 & 0 \\ 1 & 0 & 0 \end{pmatrix} \tag{M18}$$

$$\begin{pmatrix} h & -l & -k \end{pmatrix} = \begin{pmatrix} h & k & l \end{pmatrix} \cdot \begin{pmatrix} 1 & 0 & 0 \\ 0 & 0 & -1 \\ 0 & -1 & 0 \end{pmatrix} \tag{M19}$$

$$\begin{pmatrix} h & l & k \end{pmatrix} = \begin{pmatrix} h & k & l \end{pmatrix} \cdot \begin{pmatrix} 1 & 0 & 0 \\ 0 & 0 & 1 \\ 0 & 1 & 0 \end{pmatrix} \tag{M20}$$

$$\begin{pmatrix} l & h & k \end{pmatrix} = \begin{pmatrix} h & k & l \end{pmatrix} \cdot \begin{pmatrix} 0 & 1 & 0 \\ 0 & 0 & 1 \\ 1 & 0 & 0 \end{pmatrix} \tag{M21}$$

$$\begin{pmatrix} k & l & h \end{pmatrix} = \begin{pmatrix} h & k & l \end{pmatrix} \cdot \begin{pmatrix} 0 & 0 & 1 \\ 1 & 0 & 0 \\ 0 & 1 & 0 \end{pmatrix} \tag{M22}$$

$$\begin{pmatrix} -l & -h & k \end{pmatrix} = \begin{pmatrix} h & k & l \end{pmatrix} \cdot \begin{pmatrix} 0 & -1 & 0 \\ 0 & 0 & 1 \\ -1 & 0 & 0 \end{pmatrix} \tag{M23}$$

$$\begin{pmatrix} -k & l & -h \end{pmatrix} = \begin{pmatrix} h & k & l \end{pmatrix} \cdot \begin{pmatrix} 0 & 0 & -1 \\ -1 & 0 & 0 \\ 0 & 1 & 0 \end{pmatrix} \tag{M24}$$

$$\begin{pmatrix} l & -h & -k \end{pmatrix} = \begin{pmatrix} h & k & l \end{pmatrix} \cdot \begin{pmatrix} 0 & -1 & 0 \\ 0 & 0 & -1 \\ 1 & 0 & 0 \end{pmatrix} \tag{M25}$$

$$\begin{pmatrix} -k & -l & h \end{pmatrix} = \begin{pmatrix} h & k & l \end{pmatrix} \cdot \begin{pmatrix} 0 & 0 & 1 \\ -1 & 0 & 0 \\ 0 & -1 & 0 \end{pmatrix} \tag{M26}$$

$$\begin{pmatrix} -l & h & -k \end{pmatrix} = \begin{pmatrix} h & k & l \end{pmatrix} \cdot \begin{pmatrix} 0 & 1 & 0 \\ 0 & 0 & -1 \\ -1 & 0 & 0 \end{pmatrix} \tag{M27}$$

$$\begin{pmatrix} k & -l & -h \end{pmatrix} = \begin{pmatrix} h & k & l \end{pmatrix} \cdot \begin{pmatrix} 0 & 0 & -1 \\ 1 & 0 & 0 \\ 0 & -1 & 0 \end{pmatrix} \tag{M28}$$

$$\begin{pmatrix} -l & -h & -k \end{pmatrix} = \begin{pmatrix} h & k & l \end{pmatrix} \cdot \begin{pmatrix} 0 & -1 & 0 \\ 0 & 0 & -1 \\ -1 & 0 & 0 \end{pmatrix} \tag{M29}$$

$$\begin{pmatrix} -k & -l & -h \end{pmatrix} = \begin{pmatrix} h & k & l \end{pmatrix} \cdot \begin{pmatrix} 0 & 0 & -1 \\ -1 & 0 & 0 \\ 0 & -1 & 0 \end{pmatrix} \tag{M30}$$

$$\begin{pmatrix} l & h & -k \end{pmatrix} = \begin{pmatrix} h & k & l \end{pmatrix} \cdot \begin{pmatrix} 0 & 1 & 0 \\ 0 & 0 & -1 \\ 1 & 0 & 0 \end{pmatrix} \tag{M31}$$

$$\begin{pmatrix} k & -l & h \end{pmatrix} = \begin{pmatrix} h & k & l \end{pmatrix} \cdot \begin{pmatrix} 0 & 0 & 1 \\ 1 & 0 & 0 \\ 0 & -1 & 0 \end{pmatrix} \tag{M32}$$

$$\begin{pmatrix} -l & h & k \end{pmatrix} = \begin{pmatrix} h & k & l \end{pmatrix} \cdot \begin{pmatrix} 0 & 1 & 0 \\ 0 & 0 & 1 \\ -1 & 0 & 0 \end{pmatrix} \tag{M33}$$

$$\begin{pmatrix} k & l & -h \end{pmatrix} = \begin{pmatrix} h & k & l \end{pmatrix} \cdot \begin{pmatrix} 0 & 0 & -1 \\ 1 & 0 & 0 \\ 0 & 1 & 0 \end{pmatrix} \tag{M34}$$

$$\begin{pmatrix} l & -h & k \end{pmatrix} = \begin{pmatrix} h & k & l \end{pmatrix} \cdot \begin{pmatrix} 0 & -1 & 0 \\ 0 & 0 & 1 \\ 1 & 0 & 0 \end{pmatrix} \tag{M35}$$

$$\begin{pmatrix} -k & l & h \end{pmatrix} = \begin{pmatrix} h & k & l \end{pmatrix} \cdot \begin{pmatrix} 0 & 0 & 1 \\ -1 & 0 & 0 \\ 0 & 1 & 0 \end{pmatrix} \tag{M36}$$

$$-k \quad h \quad l \quad = \quad h \quad k \quad l \quad \cdot \quad \begin{pmatrix} 0 & 1 & 0 \\ -1 & 0 & 0 \\ 0 & 0 & 1 \end{pmatrix} \tag{M37}$$

$$k \quad -h \quad l \quad = \quad h \quad k \quad l \quad \cdot \quad \begin{pmatrix} 0 & -1 & 0 \\ 1 & 0 & 0 \\ 0 & 0 & 1 \end{pmatrix} \tag{M38}$$

$$h \quad -l \quad k \quad = \quad h \quad k \quad l \quad \cdot \quad \begin{pmatrix} 1 & 0 & 0 \\ 0 & 0 & 1 \\ 0 & -1 & 0 \end{pmatrix} \tag{M39}$$

$$h \quad l \quad -k \quad = \quad h \quad k \quad l \quad \cdot \quad \begin{pmatrix} 1 & 0 & 0 \\ 0 & 0 & -1 \\ 0 & 1 & 0 \end{pmatrix} \tag{M40}$$

$$l \quad k \quad -h \quad = \quad h \quad k \quad l \quad \cdot \quad \begin{pmatrix} 0 & 0 & -1 \\ 0 & 1 & 0 \\ 1 & 0 & 0 \end{pmatrix} \tag{M41}$$

$$-l \quad k \quad h \quad = \quad h \quad k \quad l \quad \cdot \quad \begin{pmatrix} 0 & 0 & 1 \\ 0 & 1 & 0 \\ -1 & 0 & 0 \end{pmatrix} \tag{M42}$$

$$k \quad -h \quad -l \quad = \quad h \quad k \quad l \quad \cdot \quad \begin{pmatrix} 0 & -1 & 0 \\ 1 & 0 & 0 \\ 0 & 0 & -1 \end{pmatrix} \tag{M43}$$

$$-k \quad h \quad -l \quad = \quad h \quad k \quad l \quad \cdot \quad \begin{pmatrix} 0 & 1 & 0 \\ -1 & 0 & 0 \\ 0 & 0 & -1 \end{pmatrix} \tag{M44}$$

$$-h \quad l \quad -k \quad = \quad h \quad k \quad l \quad \cdot \quad \begin{pmatrix} -1 & 0 & 0 \\ 0 & 0 & -1 \\ 0 & 1 & 0 \end{pmatrix} \tag{M45}$$

$$\begin{pmatrix} -h & -l & k \end{pmatrix} = \begin{pmatrix} h & k & l \end{pmatrix} \cdot \begin{pmatrix} -1 & 0 & 0 \\ 0 & 0 & 1 \\ 0 & -1 & 0 \end{pmatrix} \tag{M46}$$

$$\begin{pmatrix} -l & -k & h \end{pmatrix} = \begin{pmatrix} h & k & l \end{pmatrix} \cdot \begin{pmatrix} 0 & 0 & 1 \\ 0 & -1 & 0 \\ -1 & 0 & 0 \end{pmatrix} \tag{M47}$$

$$\begin{pmatrix} l & -k & -h \end{pmatrix} = \begin{pmatrix} h & k & l \end{pmatrix} \cdot \begin{pmatrix} 0 & 0 & -1 \\ 0 & -1 & 0 \\ 1 & 0 & 0 \end{pmatrix} \tag{M48}$$

https://doi.org/10.1107/S1600576726003031

Table 1: P - lattice [0 0 1]

[*uvw*]	*hkl A*	*hkl B*	*hkl C*	M No.
0 0 1	1 0 0	0 1 0	1 -1 0	M1, M14
0 0 1	-1 0 0	0 -1 0	-1 1 0	M2, M5
0 0 -1	1 0 0	0 -1 0	1 1 0	M3, M13
0 0 -1	-1 0 0	0 1 0	-1 -1 0	M4, M12
0 0 -1	0 1 0	1 0 0	-1 1 0	M6, M16
0 0 -1	0 -1 0	-1 0 0	1 -1 0	M7, M15
1 0 0	0 0 1	0 -1 0	0 1 1	M8, M47
-1 0 0	0 0 -1	0 -1 0	0 1 -1	M9, M48
0 1 0	-1 0 0	0 0 1	-1 0 -1	M10, M46
0 -1 0	-1 0 0	0 0 -1	-1 0 1	M11, M45
1 0 0	0 0 -1	0 1 0	0 -1 -1	M17, M41
-1 0 0	0 0 1	0 1 0	0 -1 1	M18, M42
0 1 0	1 0 0	0 0 -1	1 0 1	M19, M40
0 -1 0	1 0 0	0 0 1	1 0 -1	M20, M39
1 0 0	0 1 0	0 0 1	0 1 -1	M21, M33
0 1 0	0 0 1	1 0 0	-1 0 1	M22, M32
-1 0 0	0 -1 0	0 0 1	0 -1 -1	M23, M35
0 1 0	0 0 -1	-1 0 0	1 0 -1	M24, M30
1 0 0	0 -1 0	0 0 -1	0 -1 1	M25, M29
0 -1 0	0 0 1	-1 0 0	1 0 1	M26, M36
-1 0 0	0 1 0	0 0 -1	0 1 1	M27, M31
0 -1 0	0 0 -1	1 0 0	-1 0 -1	M28, M34
0 0 1	0 1 0	-1 0 0	1 1 0	M37, M44
0 0 1	0 -1 0	1 0 0	-1 -1 0	M38, M43

Table 2: I - lattice [0 0 1]

[*uvw*]	*hkl A*	*hkl B*	*hkl C*	M No.
0 0 1	1 1 0	-1 1 0	2 0 0	M1, M14
0 0 1	-1 -1 0	1 -1 0	-2 0 0	M2, M5
0 0 -1	1 -1 0	-1 -1 0	2 0 0	M3, M13
0 0 -1	-1 1 0	1 1 0	-2 0 0	M4, M12
0 0 -1	1 1 0	1 -1 0	0 2 0	M6, M16
0 0 -1	-1 -1 0	-1 1 0	0 -2 0	M7, M15
1 0 0	0 -1 1	0 -1 -1	0 0 2	M8, M47
-1 0 0	0 -1 -1	0 -1 1	0 0 -2	M9, M48
0 1 0	-1 0 1	1 0 1	-2 0 0	M10, M46
0 -1 0	-1 0 -1	1 0 -1	-2 0 0	M11, M45
1 0 0	0 1 -1	0 1 1	0 0 -2	M17, M41
-1 0 0	0 1 1	0 1 -1	0 0 2	M18, M42
0 1 0	1 0 -1	-1 0 -1	2 0 0	M19, M40
0 -1 0	1 0 1	-1 0 1	2 0 0	M20, M39
1 0 0	0 1 1	0 -1 1	0 2 0	M21, M33
0 1 0	1 0 1	1 0 -1	0 0 2	M22, M32
-1 0 0	0 -1 1	0 1 1	0 -2 0	M23, M35
0 1 0	-1 0 -1	-1 0 1	0 0 -2	M24, M30
1 0 0	0 -1 -1	0 1 -1	0 -2 0	M25, M29
0 -1 0	-1 0 1	-1 0 -1	0 0 2	M26, M36
-1 0 0	0 1 -1	0 -1 -1	0 2 0	M27, M31
0 -1 0	1 0 -1	1 0 1	0 0 -2	M28, M34
0 0 1	1 -1 0	1 1 0	0 -2 0	M38, M43
0 0 1	-1 1 0	-1 -1 0	0 2 0	M37, M44

Table 3: F - lattice [0 0 1]

[uvw]	hkl A	hkl B	hkl C	M No.
0 0 1	2 0 0	0 2 0	2 -2 0	M1, M14
0 0 1	-2 0 0	0 -2 0	-2 2 0	M2, M5
0 0 -1	2 0 0	0 -2 0	2 2 0	M3, M13
0 0 -1	-2 0 0	0 2 0	-2 -2 0	M4, M12
0 0 -1	0 2 0	2 0 0	-2 2 0	M6, M16
0 0 -1	0 -2 0	-2 0 0	2 -2 0	M7, M15
1 0 0	0 0 2	0 -2 0	0 2 2	M8, M47
-1 0 0	0 0 -2	0 -2 0	0 2 -2	M9, M48
0 1 0	-2 0 0	0 0 2	-2 0 -2	M10, M46
0 -1 0	-2 0 0	0 0 -2	-2 0 2	M11, M45
1 0 0	0 0 -2	0 2 0	0 -2 -2	M17, M41
-1 0 0	0 0 2	0 2 0	0 -2 2	M18, M42
0 1 0	2 0 0	0 0 -2	2 0 2	M19, M40
0 -1 0	2 0 0	0 0 2	2 0 -2	M20, M39
1 0 0	0 2 0	0 0 2	0 2 -2	M21, M33
0 1 0	0 0 2	2 0 0	-2 0 2	M22, M32
-1 0 0	0 -2 0	0 0 2	0 -2 -2	M23, M35
0 1 0	0 0 -2	-2 0 0	2 0 -2	M24, M30
1 0 0	0 -2 0	0 0 -2	0 -2 2	M25, M29
0 -1 0	0 0 2	-2 0 0	2 0 2	M26, M36
-1 0 0	0 2 0	0 0 -2	0 2 2	M27, M31
0 -1 0	0 0 -2	2 0 0	-2 0 -2	M28, M34
0 0 1	0 2 0	-2 0 0	2 2 0	M37, M44
0 0 1	0 -2 0	2 0 0	-2 -2 0	M38, M43

Table 4: P - lattice [0 1 1]

[uvw]	hkl A	hkl B	hkl C	M No.
0 1 1	1 0 0	0 1 -1	1 -1 1	M1, M19
0 1 1	-1 0 0	0 -1 1	-1 1 -1	M2, M10
0 -1 -1	1 0 0	0 -1 1	1 1 -1	M3, M20
0 1 -1	-1 0 0	0 1 1	-1 -1 -1	M4, M46
0 -1 1	-1 0 0	0 -1 -1	-1 1 1	M5, M45
1 0 -1	0 1 0	1 0 1	-1 1 -1	M6, M33
-1 0 -1	0 -1 0	-1 0 1	1 -1 -1	M7, M35
1 -1 0	0 0 1	-1 -1 0	1 1 1	M8, M36
-1 -1 0	0 0 -1	1 -1 0	-1 1 -1	M9, M34
0 -1 -1	-1 0 0	0 1 -1	-1 -1 1	M11, M12
0 1 -1	1 0 0	0 -1 -1	1 1 1	M13, M40
0 -1 1	1 0 0	0 1 1	1 -1 -1	M14, M39
1 0 -1	0 -1 0	-1 0 -1	1 -1 1	M15, M25
-1 0 -1	0 1 0	1 0 -1	-1 1 1	M16, M27
1 -1 0	0 0 -1	1 1 0	-1 -1 -1	M17, M28
-1 -1 0	0 0 1	-1 1 0	1 -1 1	M18, M26
1 0 1	0 1 0	-1 0 1	1 1 -1	M21, M44
1 1 0	0 0 1	1 -1 0	-1 1 1	M22, M47
-1 0 1	0 -1 0	1 0 1	-1 -1 -1	M23, M43
-1 1 0	0 0 -1	-1 -1 0	1 1 -1	M24, M48
1 0 1	0 -1 0	1 0 -1	-1 -1 1	M29, M38
1 1 0	0 0 -1	-1 1 0	1 -1 -1	M30, M41
-1 0 1	0 1 0	-1 0 -1	1 1 1	M31, M37
-1 1 0	0 0 1	1 1 0	-1 -1 1	M32, M42

Table 5: I - lattice [0 1 1]

[*uvw*]	*hkl A*	*hkl B*	*hkl C*	M No.
0 1 1	0 1 -1	-2 0 0	2 1 -1	M1, M19
0 1 1	0 -1 1	2 0 0	-2 -1 1	M2, M10
0 -1 -1	0 -1 1	-2 0 0	2 -1 1	M3, M20
0 1 -1	0 1 1	2 0 0	-2 1 1	M4, M46
0 -1 1	0 -1 -1	2 0 0	-2 -1 -1	M5, M45
1 0 -1	1 0 1	0 -2 0	1 2 1	M6, M33
-1 0 -1	-1 0 1	0 2 0	-1 -2 1	M7, M35
1 -1 0	-1 -1 0	0 0 -2	-1 -1 2	M8, M36
-1 -1 0	1 -1 0	0 0 2	1 -1 -2	M9, M34
0 -1 -1	0 1 -1	2 0 0	-2 1 -1	M11, M12
0 1 -1	0 -1 -1	-2 0 0	2 -1 -1	M13, M40
0 -1 1	0 1 1	-2 0 0	2 1 1	M14, M39
1 0 -1	-1 0 -1	0 2 0	-1 -2 -1	M15, M25
-1 0 -1	1 0 -1	0 -2 0	1 2 -1	M16, M27
1 -1 0	1 1 0	0 0 2	1 1 -2	M17, M28
-1 -1 0	-1 1 0	0 0 -2	-1 1 2	M18, M26
1 0 1	-1 0 1	0 -2 0	-1 2 1	M21, M44
1 1 0	1 -1 0	0 0 -2	1 -1 2	M22, M47
-1 0 1	1 0 1	0 2 0	1 -2 1	M23, M43
-1 1 0	-1 -1 0	0 0 2	-1 -1 -2	M24, M48
1 0 1	1 0 -1	0 2 0	1 -2 -1	M29, M38
1 1 0	-1 1 0	0 0 2	-1 1 -2	M30, M41
-1 0 1	-1 0 -1	0 -2 0	-1 2 -1	M31, M37
-1 1 0	1 1 0	0 0 -2	1 1 2	M32, M42

Table 6: F - lattice [0 1 1]

[*uvw*]	*hkl A*	*hkl B*	*hkl C*	M No.
0 1 1	1 1 -1	-1 1 -1	2 0 0	M1, M19
0 1 1	-1 -1 1	1 -1 1	-2 0 0	M2, M10
0 -1 -1	1 -1 1	-1 -1 1	2 0 0	M3, M20
0 1 -1	-1 1 1	1 1 1	-2 0 0	M4, M46
0 -1 1	-1 -1 -1	1 -1 -1	-2 0 0	M5, M45
1 0 -1	1 1 1	1 -1 1	0 2 0	M6, M33
-1 0 -1	-1 -1 1	-1 1 1	0 -2 0	M7, M35
1 -1 0	-1 -1 1	-1 -1 -1	0 0 2	M8, M36
-1 -1 0	1 -1 -1	1 -1 1	0 0 -2	M9, M34
0 -1 -1	-1 1 -1	1 1 -1	-2 0 0	M11, M12
0 1 -1	1 -1 -1	-1 -1 -1	2 0 0	M13, M40
0 -1 1	1 1 1	-1 1 1	2 0 0	M14, M39
1 0 -1	-1 -1 -1	-1 1 -1	0 -2 0	M15, M25
-1 0 -1	1 1 -1	1 -1 -1	0 2 0	M16, M27
1 -1 0	1 1 -1	1 1 1	0 0 -2	M17, M28
-1 -1 0	-1 1 1	-1 1 -1	0 0 2	M18, M26
1 0 1	-1 1 1	-1 -1 1	0 2 0	M21, M44
1 1 0	1 -1 1	1 -1 -1	0 0 2	M22, M47
-1 0 1	1 -1 1	1 1 1	0 -2 0	M23, M43
-1 1 0	-1 -1 -1	-1 -1 1	0 0 -2	M24, M48
1 0 1	1 -1 -1	1 1 -1	0 -2 0	M29, M38
1 1 0	-1 1 -1	-1 1 1	0 0 -2	M30, M41
-1 0 1	-1 1 -1	-1 -1 -1	0 2 0	M31, M37
-1 1 0	1 1 1	1 1 -1	0 0 2	M32, M42

Table 7: P - lattice [1 1 1]

[*uvw*]	*hkl A*	*hkl B*	*hkl C*	M No.
1 1 1	1 0 -1	0 1 -1	1 -1 0	M1
1 1 1	-1 0 1	0 -1 1	-1 1 0	M2
1 -1 -1	1 0 1	0 -1 1	1 1 0	M3
-1 1 -1	-1 0 1	0 1 1	-1 -1 0	M4
-1 -1 1	-1 0 -1	0 -1 -1	-1 1 0	M5
1 1 -1	0 1 1	1 0 1	-1 1 0	M6
-1 -1 -1	0 -1 1	-1 0 1	1 -1 0	M7
1 -1 1	-1 0 1	-1 -1 0	0 1 1	M8
-1 -1 -1	1 0 -1	1 -1 0	0 1 -1	M9
-1 1 1	-1 -1 0	0 -1 1	-1 0 -1	M10
-1 -1 -1	-1 1 0	0 1 -1	-1 0 1	M11
1 -1 -1	-1 0 -1	0 1 -1	-1 -1 0	M12
-1 1 -1	1 0 -1	0 -1 -1	1 1 0	M13
-1 -1 1	1 0 1	0 1 1	1 -1 0	M14
1 1 -1	0 -1 -1	-1 0 -1	1 -1 0	M15
-1 -1 -1	0 1 -1	1 0 -1	-1 1 0	M16
1 -1 1	1 0 -1	1 1 0	0 -1 -1	M17
-1 -1 -1	-1 0 1	-1 1 0	0 -1 1	M18
-1 1 1	1 1 0	0 1 -1	1 0 1	M19
-1 -1 -1	1 -1 0	0 -1 1	1 0 -1	M20
1 1 1	-1 1 0	-1 0 1	0 1 -1	M21
1 1 1	0 -1 1	1 -1 0	-1 0 1	M22
-1 -1 1	1 -1 0	1 0 1	0 -1 -1	M23
-1 1 -1	0 -1 -1	-1 -1 0	1 0 -1	M24
1 -1 -1	-1 -1 0	-1 0 -1	0 -1 1	M25
-1 -1 1	0 1 1	-1 1 0	1 0 1	M26
-1 1 -1	1 1 0	1 0 -1	0 1 1	M27
1 -1 -1	0 1 -1	1 1 0	-1 0 -1	M28
1 1 1	1 -1 0	1 0 -1	0 -1 1	M29
1 1 1	0 1 -1	-1 1 0	1 0 -1	M30
-1 -1 1	-1 1 0	-1 0 -1	0 1 1	M31
-1 1 -1	0 1 1	1 1 0	-1 0 1	M32
1 -1 -1	1 1 0	1 0 1	0 1 -1	M33
-1 -1 1	0 -1 -1	1 -1 0	-1 0 -1	M34
-1 1 -1	-1 -1 0	-1 0 1	0 -1 -1	M35
1 -1 -1	0 -1 1	-1 -1 0	1 0 1	M36
-1 1 1	0 1 -1	-1 0 -1	1 1 0	M37
1 -1 1	0 -1 -1	1 0 -1	-1 -1 0	M38
1 -1 1	1 1 0	0 1 1	1 0 -1	M39
1 1 -1	1 -1 0	0 -1 -1	1 0 1	M40
1 1 -1	-1 0 -1	-1 1 0	0 -1 -1	M41
-1 1 1	1 0 1	1 1 0	0 -1 1	M42
-1 1 1	0 -1 1	1 0 1	-1 -1 0	M43
1 -1 1	0 1 1	-1 0 1	1 1 0	M44
1 -1 1	-1 -1 0	0 -1 -1	-1 0 1	M45
1 1 -1	-1 1 0	0 1 1	-1 0 -1	M46
1 1 -1	1 0 1	1 -1 0	0 1 1	M47
-1 1 1	-1 0 -1	-1 -1 0	0 1 -1	M48

Table 8: I - lattice [1 1 1]

[uvw]	hkl A	hkl B	hkl C	M No.
1 1 1	0 1 -1	-1 1 0	1 0 -1	M1
1 1 1	0 -1 1	1 -1 0	-1 0 1	M2
1 -1 -1	0 -1 1	-1 -1 0	1 0 1	M3
-1 1 -1	0 1 1	1 1 0	-1 0 1	M4
-1 -1 1	0 -1 -1	1 -1 0	-1 0 -1	M5
1 1 -1	1 0 1	1 -1 0	0 1 1	M6
-1 -1 -1	-1 0 1	-1 1 0	0 -1 1	M7
1 -1 1	-1 -1 0	0 -1 -1	-1 0 1	M8
-1 -1 -1	1 -1 0	0 -1 1	1 0 -1	M9
-1 1 1	0 -1 1	1 0 1	-1 -1 0	M10
-1 -1 -1	0 1 -1	1 0 -1	-1 1 0	M11
1 -1 -1	0 1 -1	1 1 0	-1 0 -1	M12
-1 1 -1	0 -1 -1	-1 -1 0	1 0 -1	M13
-1 -1 1	0 1 1	-1 1 0	1 0 1	M14
1 1 -1	-1 0 -1	-1 1 0	0 -1 -1	M15
-1 -1 -1	1 0 -1	1 -1 0	0 1 -1	M16
1 -1 1	1 1 0	0 1 1	1 0 -1	M17
-1 -1 -1	-1 1 0	0 1 -1	-1 0 1	M18
-1 1 1	0 1 -1	-1 0 -1	1 1 0	M19
-1 -1 -1	0 -1 1	-1 0 1	1 -1 0	M20
1 1 1	-1 0 1	0 -1 1	-1 1 0	M21
1 1 1	1 -1 0	1 0 -1	0 -1 1	M22
-1 -1 1	1 0 1	0 1 1	1 -1 0	M23
-1 1 -1	-1 -1 0	-1 0 1	0 -1 -1	M24
1 -1 -1	-1 0 -1	0 1 -1	-1 -1 0	M25
-1 -1 1	-1 1 0	-1 0 -1	0 1 1	M26
-1 1 -1	1 0 -1	0 -1 -1	1 1 0	M27
1 -1 -1	1 1 0	1 0 1	0 1 -1	M28
1 1 1	1 0 -1	0 1 -1	1 -1 0	M29
1 1 1	-1 1 0	-1 0 1	0 1 -1	M30
-1 -1 1	-1 0 -1	0 -1 -1	-1 1 0	M31
-1 1 -1	1 1 0	1 0 -1	0 1 1	M32
1 -1 -1	1 0 1	0 -1 1	1 1 0	M33
-1 -1 1	1 -1 0	1 0 1	0 -1 -1	M34
-1 1 -1	-1 0 1	0 1 1	-1 -1 0	M35
1 -1 -1	-1 -1 0	-1 0 -1	0 -1 1	M36
-1 1 1	-1 0 -1	-1 -1 0	0 1 -1	M37
1 -1 1	1 0 -1	1 1 0	0 -1 -1	M38
1 -1 1	0 1 1	-1 0 1	1 1 0	M39
1 1 -1	0 -1 -1	-1 0 -1	1 -1 0	M40
1 1 -1	-1 1 0	0 1 1	-1 0 -1	M41
-1 1 1	1 1 0	0 1 -1	1 0 1	M42
-1 1 1	1 0 1	1 1 0	0 -1 1	M43
1 -1 1	-1 0 1	-1 -1 0	0 1 1	M44
1 -1 1	0 -1 -1	1 0 -1	-1 -1 0	M45
1 1 -1	0 1 1	1 0 1	-1 1 0	M46
1 1 -1	1 -1 0	0 -1 -1	1 0 1	M47
-1 1 1	-1 -1 0	0 -1 1	-1 0 -1	M48

Table 9: F - lattice [1 1 1]

[*uvw*]	*hkl A*	*hkl B*	*hkl C*	M No.
1 1 1	2 0 -2	0 2 -2	2 -2 0	M1
1 1 1	-2 0 2	0 -2 2	-2 2 0	M2
1 -1 -1	2 0 2	0 -2 2	2 2 0	M3
-1 1 -1	-2 0 2	0 2 2	-2 -2 0	M4
-1 -1 1	-2 0 -2	0 -2 -2	-2 2 0	M5
1 1 -1	0 2 2	2 0 2	-2 2 0	M6
-1 -1 -1	0 -2 2	-2 0 2	2 -2 0	M7
1 -1 1	-2 0 2	-2 -2 0	0 2 2	M8
-1 -1 -1	2 0 -2	2 -2 0	0 2 -2	M9
-1 1 1	-2 -2 0	0 -2 2	-2 0 -2	M10
-1 -1 -1	-2 2 0	0 2 -2	-2 0 2	M11
1 -1 -1	-2 0 -2	0 2 -2	-2 -2 0	M12
-1 1 -1	2 0 -2	0 -2 -2	2 2 0	M13
-1 -1 1	2 0 2	0 2 2	2 -2 0	M14
1 1 -1	0 -2 -2	-2 0 -2	2 -2 0	M15
-1 -1 -1	0 2 -2	2 0 -2	-2 2 0	M16
1 -1 1	2 0 -2	2 2 0	0 -2 -2	M17
-1 -1 -1	-2 0 2	-2 2 0	0 -2 2	M18
-1 1 1	2 2 0	0 2 -2	2 0 2	M19
-1 -1 -1	2 -2 0	0 -2 2	2 0 -2	M20
1 1 1	-2 2 0	-2 0 2	0 2 -2	M21
1 1 1	0 -2 2	2 -2 0	-2 0 2	M22
-1 -1 1	2 -2 0	2 0 2	0 -2 -2	M23
-1 1 -1	0 -2 -2	-2 -2 0	2 0 -2	M24
1 -1 -1	-2 -2 0	-2 0 -2	0 -2 2	M25
-1 -1 1	0 2 2	-2 2 0	2 0 2	M26
-1 1 -1	2 2 0	2 0 -2	0 2 2	M27
1 -1 -1	0 2 -2	2 2 0	-2 0 -2	M28
1 1 1	2 -2 0	2 0 -2	0 -2 2	M29
1 1 1	0 2 -2	-2 2 0	2 0 -2	M30
-1 -1 1	-2 2 0	-2 0 -2	0 2 2	M31
-1 1 -1	0 2 2	2 2 0	-2 0 2	M32
1 -1 -1	2 2 0	2 0 2	0 2 -2	M33
-1 -1 1	0 -2 -2	2 -2 0	-2 0 -2	M34
-1 1 -1	-2 -2 0	-2 0 2	0 -2 -2	M35
1 -1 -1	0 -2 2	-2 -2 0	2 0 2	M36
-1 1 1	0 2 -2	-2 0 -2	2 2 0	M37
1 -1 1	0 -2 -2	2 0 -2	-2 -2 0	M38
1 -1 1	2 2 0	0 2 2	2 0 -2	M39
1 1 -1	2 -2 0	0 -2 -2	2 0 2	M40
1 1 -1	-2 0 -2	-2 2 0	0 -2 -2	M41
-1 1 1	2 0 2	2 2 0	0 -2 2	M42
-1 1 1	0 -2 2	2 0 2	-2 -2 0	M43
1 -1 1	0 2 2	-2 0 2	2 2 0	M44
1 -1 1	-2 -2 0	0 -2 -2	-2 0 2	M45
1 1 -1	-2 2 0	0 2 2	-2 0 -2	M46
1 1 -1	2 0 2	2 -2 0	0 2 2	M47
-1 1 1	-2 0 -2	-2 -2 0	0 2 -2	M48

Table 10: P - lattice [0 1 2]

[*uvw*]	*hkl A*	*hkl B*	*hkl C*	M No.
0 1 2	1 0 0	0 2 -1	1 -2 1	M1
0 1 2	-1 0 0	0 -2 1	-1 2 -1	M2
0 -1 -2	1 0 0	0 -2 1	1 2 -1	M3
0 1 -2	-1 0 0	0 2 1	-1 -2 -1	M4
0 -1 2	-1 0 0	0 -2 -1	-1 2 1	M5
1 0 -2	0 1 0	2 0 1	-2 1 -1	M6
-1 0 -2	0 -1 0	-2 0 1	2 -1 -1	M7
2 -1 0	0 0 1	-1 -2 0	1 2 1	M8
-2 -1 0	0 0 -1	1 -2 0	-1 2 -1	M9
0 2 1	-1 0 0	0 -1 2	-1 1 -2	M10
0 -2 -1	-1 0 0	0 1 -2	-1 -1 2	M11
0 -1 -2	-1 0 0	0 2 -1	-1 -2 1	M12
0 1 -2	1 0 0	0 -2 -1	1 2 1	M13
0 -1 2	1 0 0	0 2 1	1 -2 -1	M14
1 0 -2	0 -1 0	-2 0 -1	2 -1 1	M15
-1 0 -2	0 1 0	2 0 -1	-2 1 1	M16
2 -1 0	0 0 -1	1 2 0	-1 -2 -1	M17
-2 -1 0	0 0 1	-1 2 0	1 -2 1	M18
0 2 1	1 0 0	0 1 -2	1 -1 2	M19
0 -2 -1	1 0 0	0 -1 2	1 1 -2	M20
2 0 1	0 1 0	-1 0 2	1 1 -2	M21
1 2 0	0 0 1	2 -1 0	-2 1 1	M22
-2 0 1	0 -1 0	1 0 2	-1 -1 -2	M23
-1 2 0	0 0 -1	-2 -1 0	2 1 -1	M24
2 0 -1	0 -1 0	-1 0 -2	1 -1 2	M25
-1 -2 0	0 0 1	-2 1 0	2 -1 1	M26
-2 0 -1	0 1 0	1 0 -2	-1 1 2	M27
1 -2 0	0 0 -1	2 1 0	-2 -1 -1	M28
2 0 1	0 -1 0	1 0 -2	-1 -1 2	M29
1 2 0	0 0 -1	-2 1 0	2 -1 -1	M30
-2 0 1	0 1 0	-1 0 -2	1 1 2	M31
-1 2 0	0 0 1	2 1 0	-2 -1 1	M32
2 0 -1	0 1 0	1 0 2	-1 1 -2	M33
-1 -2 0	0 0 -1	2 -1 0	-2 1 -1	M34
-2 0 -1	0 -1 0	-1 0 2	1 -1 -2	M35
1 -2 0	0 0 1	-2 -1 0	2 1 1	M36
-1 0 2	0 1 0	-2 0 -1	2 1 1	M37
1 0 2	0 -1 0	2 0 -1	-2 -1 1	M38
0 -2 1	1 0 0	0 1 2	1 -1 -2	M39
0 2 -1	1 0 0	0 -1 -2	1 1 2	M40
2 1 0	0 0 -1	-1 2 0	1 -2 -1	M41
-2 1 0	0 0 1	1 2 0	-1 -2 1	M42
-1 0 2	0 -1 0	2 0 1	-2 -1 -1	M43
1 0 2	0 1 0	-2 0 1	2 1 -1	M44
0 -2 1	-1 0 0	0 -1 -2	-1 1 2	M45
0 2 -1	-1 0 0	0 1 2	-1 -1 -2	M46
2 1 0	0 0 1	1 -2 0	-1 2 1	M47
-2 1 0	0 0 -1	-1 -2 0	1 2 -1	M48

Table 11: I - lattice [0 1 2]

[uvw]	hkl A	hkl B	hkl C	M No.
0 1 2	2 0 0	1 2 -1	1 -2 1	M1
0 1 2	-2 0 0	-1 -2 1	-1 2 -1	M2
0 -1 -2	2 0 0	1 -2 1	1 2 -1	M3
0 1 -2	-2 0 0	-1 2 1	-1 -2 -1	M4
0 -1 2	-2 0 0	-1 -2 -1	-1 2 1	M5
1 0 -2	0 2 0	2 1 1	-2 1 -1	M6
-1 0 -2	0 -2 0	-2 -1 1	2 -1 -1	M7
2 -1 0	0 0 2	-1 -2 1	1 2 1	M8
-2 -1 0	0 0 -2	1 -2 -1	-1 2 -1	M9
0 2 1	-2 0 0	-1 -1 2	-1 1 -2	M10
0 -2 -1	-2 0 0	-1 1 -2	-1 -1 2	M11
0 -1 -2	-2 0 0	-1 2 -1	-1 -2 1	M12
0 1 -2	2 0 0	1 -2 -1	1 2 1	M13
0 -1 2	2 0 0	1 2 1	1 -2 -1	M14
1 0 -2	0 -2 0	-2 -1 -1	2 -1 1	M15
-1 0 -2	0 2 0	2 1 -1	-2 1 1	M16
2 -1 0	0 0 -2	1 2 -1	-1 -2 -1	M17
-2 -1 0	0 0 2	-1 2 1	1 -2 1	M18
0 2 1	2 0 0	1 1 -2	1 -1 2	M19
0 -2 -1	2 0 0	1 -1 2	1 1 -2	M20
2 0 1	0 2 0	-1 1 2	1 1 -2	M21
1 2 0	0 0 2	2 -1 1	-2 1 1	M22
-2 0 1	0 -2 0	1 -1 2	-1 -1 -2	M23
-1 2 0	0 0 -2	-2 -1 -1	2 1 -1	M24
2 0 -1	0 -2 0	-1 -1 -2	1 -1 2	M25
-1 -2 0	0 0 2	-2 1 1	2 -1 1	M26
-2 0 -1	0 2 0	1 1 -2	-1 1 2	M27
1 -2 0	0 0 -2	2 1 -1	-2 -1 -1	M28
2 0 1	0 -2 0	1 -1 -2	-1 -1 2	M29
1 2 0	0 0 -2	-2 1 -1	2 -1 -1	M30
-2 0 1	0 2 0	-1 1 -2	1 1 2	M31
-1 2 0	0 0 2	2 1 1	-2 -1 1	M32
2 0 -1	0 2 0	1 1 2	-1 1 -2	M33
-1 -2 0	0 0 -2	2 -1 -1	-2 1 -1	M34
-2 0 -1	0 -2 0	-1 -1 2	1 -1 -2	M35
1 -2 0	0 0 2	-2 -1 1	2 1 1	M36
-1 0 2	0 2 0	-2 1 -1	2 1 1	M37
1 0 2	0 -2 0	2 -1 -1	-2 -1 1	M38
0 -2 1	2 0 0	1 1 2	1 -1 -2	M39
0 2 -1	2 0 0	1 -1 -2	1 1 2	M40
2 1 0	0 0 -2	-1 2 -1	1 -2 -1	M41
-2 1 0	0 0 2	1 2 1	-1 -2 1	M42
-1 0 2	0 -2 0	2 -1 1	-2 -1 -1	M43
1 0 2	0 2 0	-2 1 1	2 1 -1	M44
0 -2 1	-2 0 0	-1 -1 -2	-1 1 2	M45
0 2 -1	-2 0 0	-1 1 2	-1 -1 -2	M46
2 1 0	0 0 2	1 -2 1	-1 2 1	M47
-2 1 0	0 0 -2	-1 -2 -1	1 2 -1	M48

Table 12: F - lattice [0 1 2]

[*uvw*]	*hkl A*	*hkl B*	*hkl C*	M No.
0 1 2	2 0 0	0 4 -2	2 -4 2	M1
0 1 2	-2 0 0	0 -4 2	-2 4 -2	M2
0 -1 -2	2 0 0	0 -4 2	2 4 -2	M3
0 1 -2	-2 0 0	0 4 2	-2 -4 -2	M4
0 -1 2	-2 0 0	0 -4 -2	-2 4 2	M5
1 0 -2	0 2 0	4 0 2	-4 2 -2	M6
-1 0 -2	0 -2 0	-4 0 2	4 -2 -2	M7
2 -1 0	0 0 2	-2 -4 0	2 4 2	M8
-2 -1 0	0 0 -2	2 -4 0	-2 4 -2	M9
0 2 1	-2 0 0	0 -2 4	-2 2 -4	M10
0 -2 -1	-2 0 0	0 2 -4	-2 -2 4	M11
0 -1 -2	-2 0 0	0 4 -2	-2 -4 2	M12
0 1 -2	2 0 0	0 -4 -2	2 4 2	M13
0 -1 2	2 0 0	0 4 2	2 -4 -2	M14
1 0 -2	0 -2 0	-4 0 -2	4 -2 2	M15
-1 0 -2	0 2 0	4 0 -2	-4 2 2	M16
2 -1 0	0 0 -2	2 4 0	-2 -4 -2	M17
-2 -1 0	0 0 2	-2 4 0	2 -4 2	M18
0 2 1	2 0 0	0 2 -4	2 -2 4	M19
0 -2 -1	2 0 0	0 -2 4	2 2 -4	M20
2 0 1	0 2 0	-2 0 4	2 2 -4	M21
1 2 0	0 0 2	4 -2 0	-4 2 2	M22
-2 0 1	0 -2 0	2 0 4	-2 -2 -4	M23
-1 2 0	0 0 -2	-4 -2 0	4 2 -2	M24
2 0 -1	0 -2 0	-2 0 -4	2 -2 4	M25
-1 -2 0	0 0 2	-4 2 0	4 -2 2	M26
-2 0 -1	0 2 0	2 0 -4	-2 2 4	M27
1 -2 0	0 0 -2	4 2 0	-4 -2 -2	M28
2 0 1	0 -2 0	2 0 -4	-2 -2 4	M29
1 2 0	0 0 -2	-4 2 0	4 -2 -2	M30
-2 0 1	0 2 0	-2 0 -4	2 2 4	M31
-1 2 0	0 0 2	4 2 0	-4 -2 2	M32
2 0 -1	0 2 0	2 0 4	-2 2 -4	M33
-1 -2 0	0 0 -2	4 -2 0	-4 2 -2	M34
-2 0 -1	0 -2 0	-2 0 4	2 -2 -4	M35
1 -2 0	0 0 2	-4 -2 0	4 2 2	M36
-1 0 2	0 2 0	-4 0 -2	4 2 2	M37
1 0 2	0 -2 0	4 0 -2	-4 -2 2	M38
0 -2 1	2 0 0	0 2 4	2 -2 -4	M39
0 2 -1	2 0 0	0 -2 -4	2 2 4	M40
2 1 0	0 0 -2	-2 4 0	2 -4 -2	M41
-2 1 0	0 0 2	2 4 0	-2 -4 2	M42
-1 0 2	0 -2 0	4 0 2	-4 -2 -2	M43
1 0 2	0 2 0	-4 0 2	4 2 -2	M44
0 -2 1	-2 0 0	0 -2 -4	-2 2 4	M45
0 2 -1	-2 0 0	0 2 4	-2 -2 -4	M46
2 1 0	0 0 2	2 -4 0	-2 4 2	M47
-2 1 0	0 0 -2	-2 -4 0	2 4 -2	M48

Table 13: P - lattice [1 1 2]

[*uvw*]	*hkl A*	*hkl B*	*hkl C*	M No.
1 1 2	1 -1 0	1 1 -1	0 -2 1	M1
1 1 2	-1 1 0	-1 -1 1	0 2 -1	M2
1 -1 -2	1 1 0	1 -1 1	0 2 -1	M3
-1 1 -2	-1 -1 0	-1 1 1	0 -2 -1	M4
-1 -1 2	-1 1 0	-1 -1 -1	0 2 1	M5
1 1 -2	-1 1 0	1 1 1	-2 0 -1	M6
-1 -1 -2	1 -1 0	-1 -1 1	2 0 -1	M7
2 -1 1	0 1 1	-1 -1 1	1 2 0	M8
-2 -1 -1	0 1 -1	1 -1 -1	-1 2 0	M9
-1 2 1	-1 0 -1	-1 -1 1	0 1 -2	M10
-1 -2 -1	-1 0 1	-1 1 -1	0 -1 2	M11
1 -1 -2	-1 -1 0	-1 1 -1	0 -2 1	M12
-1 1 -2	1 1 0	1 -1 -1	0 2 1	M13
-1 -1 2	1 -1 0	1 1 1	0 -2 -1	M14
1 1 -2	1 -1 0	-1 -1 -1	2 0 1	M15
-1 -1 -2	-1 1 0	1 1 -1	-2 0 1	M16
2 -1 1	0 -1 -1	1 1 -1	-1 -2 0	M17
-2 -1 -1	0 -1 1	-1 1 1	1 -2 0	M18
-1 2 1	1 0 1	1 1 -1	0 -1 2	M19
-1 -2 -1	1 0 -1	1 -1 1	0 1 -2	M20
2 1 1	0 1 -1	-1 1 1	1 0 -2	M21
1 2 1	-1 0 1	1 -1 1	-2 1 0	M22
-2 -1 1	0 -1 -1	1 -1 1	-1 0 -2	M23
-1 2 -1	1 0 -1	-1 -1 -1	2 1 0	M24
2 -1 -1	0 -1 1	-1 -1 -1	1 0 2	M25
-1 -2 1	1 0 1	-1 1 1	2 -1 0	M26
-2 1 -1	0 1 1	1 1 -1	-1 0 2	M27
1 -2 -1	-1 0 -1	1 1 -1	-2 -1 0	M28
2 1 1	0 -1 1	1 -1 -1	-1 0 2	M29
1 2 1	1 0 -1	-1 1 -1	2 -1 0	M30
-2 -1 1	0 1 1	-1 1 -1	1 0 2	M31
-1 2 -1	-1 0 1	1 1 1	-2 -1 0	M32
2 -1 -1	0 1 -1	1 1 1	-1 0 -2	M33
-1 -2 1	-1 0 -1	1 -1 -1	-2 1 0	M34
-2 1 -1	0 -1 -1	-1 -1 1	1 0 -2	M35
1 -2 -1	1 0 1	-1 -1 1	2 1 0	M36
-1 1 2	1 1 0	-1 1 -1	2 0 1	M37
1 -1 2	-1 -1 0	1 -1 -1	-2 0 1	M38
1 -2 1	1 0 -1	1 1 1	0 -1 -2	M39
1 2 -1	1 0 1	1 -1 -1	0 1 2	M40
2 1 -1	0 -1 -1	-1 1 -1	1 -2 0	M41
-2 1 1	0 -1 1	1 1 1	-1 -2 0	M42
-1 1 2	-1 -1 0	1 -1 1	-2 0 -1	M43
1 -1 2	1 1 0	-1 1 1	2 0 -1	M44
1 -2 1	-1 0 1	-1 -1 -1	0 1 2	M45
1 2 -1	-1 0 -1	-1 1 1	0 -1 -2	M46
2 1 -1	0 1 1	1 -1 1	-1 2 0	M47
-2 1 1	0 1 -1	-1 -1 -1	1 2 0	M48

Table 14: I - lattice [1 1 2]

[*uvw*]	*hkl A*	*hkl B*	*hkl C*	M No.
1 1 2	1 -1 0	2 2 -2	-1 -3 2	M1
1 1 2	-1 1 0	-2 -2 2	1 3 -2	M2
1 -1 -2	1 1 0	2 -2 2	-1 3 -2	M3
-1 1 -2	-1 -1 0	-2 2 2	1 -3 -2	M4
-1 -1 2	-1 1 0	-2 -2 -2	1 3 2	M5
1 1 -2	-1 1 0	2 2 2	-3 -1 -2	M6
-1 -1 -2	1 -1 0	-2 -2 2	3 1 -2	M7
2 -1 1	0 1 1	-2 -2 2	2 3 -1	M8
-2 -1 -1	0 1 -1	2 -2 -2	-2 3 1	M9
-1 2 1	-1 0 -1	-2 -2 2	1 2 -3	M10
-1 -2 -1	-1 0 1	-2 2 -2	1 -2 3	M11
1 -1 -2	-1 -1 0	-2 2 -2	1 -3 2	M12
-1 1 -2	1 1 0	2 -2 -2	-1 3 2	M13
-1 -1 2	1 -1 0	2 2 2	-1 -3 -2	M14
1 1 -2	1 -1 0	-2 -2 -2	3 1 2	M15
-1 -1 -2	-1 1 0	2 2 -2	-3 -1 2	M16
2 -1 1	0 -1 -1	2 2 -2	-2 -3 1	M17
-2 -1 -1	0 -1 1	-2 2 2	2 -3 -1	M18
-1 2 1	1 0 1	2 2 -2	-1 -2 3	M19
-1 -2 -1	1 0 -1	2 -2 2	-1 2 -3	M20
2 1 1	0 1 -1	-2 2 2	2 -1 -3	M21
1 2 1	-1 0 1	2 -2 2	-3 2 -1	M22
-2 -1 1	0 -1 -1	2 -2 2	-2 1 -3	M23
-1 2 -1	1 0 -1	-2 -2 -2	3 2 1	M24
2 -1 -1	0 -1 1	-2 -2 -2	2 1 3	M25
-1 -2 1	1 0 1	-2 2 2	3 -2 -1	M26
-2 1 -1	0 1 1	2 2 -2	-2 -1 3	M27
1 -2 -1	-1 0 -1	2 2 -2	-3 -2 1	M28
2 1 1	0 -1 1	2 -2 -2	-2 1 3	M29
1 2 1	1 0 -1	-2 2 -2	3 -2 1	M30
-2 -1 1	0 1 1	-2 2 -2	2 -1 3	M31
-1 2 -1	-1 0 1	2 2 2	-3 -2 -1	M32
2 -1 -1	0 1 -1	2 2 2	-2 -1 -3	M33
-1 -2 1	-1 0 -1	2 -2 -2	-3 2 1	M34
-2 1 -1	0 -1 -1	-2 -2 2	2 1 -3	M35
1 -2 -1	1 0 1	-2 -2 2	3 2 -1	M36
-1 1 2	1 1 0	-2 2 -2	3 -1 2	M37
1 -1 2	-1 -1 0	2 -2 -2	-3 1 2	M38
1 -2 1	1 0 -1	2 2 2	-1 -2 -3	M39
1 2 -1	1 0 1	2 -2 -2	-1 2 3	M40
2 1 -1	0 -1 -1	-2 2 -2	2 -3 1	M41
-2 1 1	0 -1 1	2 2 2	-2 -3 -1	M42
-1 1 2	-1 -1 0	2 -2 2	-3 1 -2	M43
1 -1 2	1 1 0	-2 2 2	3 -1 -2	M44
1 -2 1	-1 0 1	-2 -2 -2	1 2 3	M45
1 2 -1	-1 0 -1	-2 2 2	1 -2 -3	M46
2 1 -1	0 1 1	2 -2 2	-2 3 -1	M47
-2 1 1	0 1 -1	-2 -2 -2	2 3 1	M48

Table 15: F - lattice [1 1 2]

[uvw]	hkl A	hkl B	hkl C	M No.
1 1 2	1 1 -1	-2 2 0	3 -1 -1	M1
1 1 2	-1 -1 1	2 -2 0	-3 1 1	M2
1 -1 -2	1 -1 1	-2 -2 0	3 1 1	M3
-1 1 -2	-1 1 1	2 2 0	-3 -1 1	M4
-1 -1 2	-1 -1 -1	2 -2 0	-3 1 -1	M5
1 1 -2	1 1 1	2 -2 0	-1 3 1	M6
-1 -1 -2	-1 -1 1	-2 2 0	1 -3 1	M7
2 -1 1	-1 -1 1	0 -2 -2	-1 1 3	M8
-2 -1 -1	1 -1 -1	0 -2 2	1 1 -3	M9
-1 2 1	-1 -1 1	2 0 2	-3 -1 -1	M10
-1 -2 -1	-1 1 -1	2 0 -2	-3 1 1	M11
1 -1 -2	-1 1 -1	2 2 0	-3 -1 -1	M12
-1 1 -2	1 -1 -1	-2 -2 0	3 1 -1	M13
-1 -1 2	1 1 1	-2 2 0	3 -1 1	M14
1 1 -2	-1 -1 -1	-2 2 0	1 -3 -1	M15
-1 -1 -2	1 1 -1	2 -2 0	-1 3 -1	M16
2 -1 1	1 1 -1	0 2 2	1 -1 -3	M17
-2 -1 -1	-1 1 1	0 2 -2	-1 -1 3	M18
-1 2 1	1 1 -1	-2 0 -2	3 1 1	M19
-1 -2 -1	1 -1 1	-2 0 2	3 -1 -1	M20
2 1 1	-1 1 1	0 -2 2	-1 3 -1	M21
1 2 1	1 -1 1	2 0 -2	-1 -1 3	M22
-2 -1 1	1 -1 1	0 2 2	1 -3 -1	M23
-1 2 -1	-1 -1 -1	-2 0 2	1 -1 -3	M24
2 -1 -1	-1 -1 -1	0 2 -2	-1 -3 1	M25
-1 -2 1	-1 1 1	-2 0 -2	1 1 3	M26
-2 1 -1	1 1 -1	0 -2 -2	1 3 1	M27
1 -2 -1	1 1 -1	2 0 2	-1 1 -3	M28
2 1 1	1 -1 -1	0 2 -2	1 -3 1	M29
1 2 1	-1 1 -1	-2 0 2	1 1 -3	M30
-2 -1 1	-1 1 -1	0 -2 -2	-1 3 1	M31
-1 2 -1	1 1 1	2 0 -2	-1 1 3	M32
2 -1 -1	1 1 1	0 -2 2	1 3 -1	M33
-1 -2 1	1 -1 -1	2 0 2	-1 -1 -3	M34
-2 1 -1	-1 -1 1	0 2 2	-1 -3 -1	M35
1 -2 -1	-1 -1 1	-2 0 -2	1 -1 3	M36
-1 1 2	-1 1 -1	-2 -2 0	1 3 -1	M37
1 -1 2	1 -1 -1	2 2 0	-1 -3 -1	M38
1 -2 1	1 1 1	-2 0 2	3 1 -1	M39
1 2 -1	1 -1 -1	-2 0 -2	3 -1 1	M40
2 1 -1	-1 1 -1	0 2 2	-1 -1 -3	M41
-2 1 1	1 1 1	0 2 -2	1 -1 3	M42
-1 1 2	1 -1 1	2 2 0	-1 -3 1	M43
1 -1 2	-1 1 1	-2 -2 0	1 3 1	M44
1 -2 1	-1 -1 -1	2 0 -2	-3 -1 1	M45
1 2 -1	-1 1 1	2 0 2	-3 1 -1	M46
2 1 -1	1 -1 1	0 -2 -2	1 1 3	M47
-2 1 1	-1 -1 -1	0 -2 2	-1 1 -3	M48

Table 16: P - lattice [1 2 2]

[*uvw*]	*hkl A*	*hkl B*	*hkl C*	M No.
1 2 2	0 -1 1	2 -1 0	-2 0 1	M1
1 2 2	0 1 -1	-2 1 0	2 0 -1	M2
1 -2 -2	0 1 -1	2 1 0	-2 0 -1	M3
-1 2 -2	0 -1 -1	-2 -1 0	2 0 -1	M4
-1 -2 2	0 1 1	-2 1 0	2 0 1	M5
2 1 -2	-1 0 -1	-1 2 0	0 -2 -1	M6
-2 -1 -2	1 0 -1	1 -2 0	0 2 -1	M7
2 -2 1	1 1 0	0 1 2	1 0 -2	M8
-2 -2 -1	-1 1 0	0 1 -2	-1 0 2	M9
-1 2 2	0 1 -1	-2 0 -1	2 1 0	M10
-1 -2 -2	0 -1 1	-2 0 1	2 -1 0	M11
1 -2 -2	0 -1 1	-2 -1 0	2 0 1	M12
-1 2 -2	0 1 1	2 1 0	-2 0 1	M13
-1 -2 2	0 -1 -1	2 -1 0	-2 0 -1	M14
2 1 -2	1 0 1	1 -2 0	0 2 1	M15
-2 -1 -2	-1 0 1	-1 2 0	0 -2 1	M16
2 -2 1	-1 -1 0	0 -1 -2	-1 0 2	M17
-2 -2 -1	1 -1 0	0 -1 2	1 0 -2	M18
-1 2 2	0 -1 1	2 0 1	-2 -1 0	M19
-1 -2 -2	0 1 -1	2 0 -1	-2 1 0	M20
2 1 2	1 0 -1	0 2 -1	1 -2 0	M21
2 2 1	-1 1 0	-1 0 2	0 1 -2	M22
-2 -1 2	-1 0 -1	0 -2 -1	-1 2 0	M23
-2 2 -1	1 1 0	1 0 -2	0 1 2	M24
2 -1 -2	1 0 1	0 -2 1	1 2 0	M25
-2 -2 1	1 -1 0	1 0 2	0 -1 -2	M26
-2 1 -2	-1 0 1	0 2 1	-1 -2 0	M27
2 -2 -1	-1 -1 0	-1 0 -2	0 -1 2	M28
2 1 2	-1 0 1	0 -2 1	-1 2 0	M29
2 2 1	1 -1 0	1 0 -2	0 -1 2	M30
-2 -1 2	1 0 1	0 2 1	1 -2 0	M31
-2 2 -1	-1 -1 0	-1 0 2	0 -1 -2	M32
2 -1 -2	-1 0 -1	0 2 -1	-1 -2 0	M33
-2 -2 1	-1 1 0	-1 0 -2	0 1 2	M34
-2 1 -2	1 0 -1	0 -2 -1	1 2 0	M35
2 -2 -1	1 1 0	1 0 2	0 1 -2	M36
-2 1 2	1 0 1	1 2 0	0 -2 1	M37
2 -1 2	-1 0 1	-1 -2 0	0 2 1	M38
1 -2 2	0 -1 -1	2 0 -1	-2 -1 0	M39
1 2 -2	0 1 1	2 0 1	-2 1 0	M40
2 2 -1	1 -1 0	0 -1 -2	1 0 2	M41
-2 2 1	-1 -1 0	0 -1 2	-1 0 -2	M42
-2 1 2	-1 0 -1	-1 -2 0	0 2 -1	M43
2 -1 2	1 0 -1	1 2 0	0 -2 -1	M44
1 -2 2	0 1 1	-2 0 1	2 1 0	M45
1 2 -2	0 -1 -1	-2 0 -1	2 -1 0	M46
2 2 -1	-1 1 0	0 1 2	-1 0 -2	M47
-2 2 1	1 1 0	0 1 -2	1 0 2	M48

Table 17: I - lattice [1 2 2]

[uvw]	hkl A	hkl B	hkl C	M No.
1 2 2	0 1 -1	-4 1 1	4 0 -2	M1
1 2 2	0 -1 1	4 -1 -1	-4 0 2	M2
1 -2 -2	0 -1 1	-4 -1 -1	4 0 2	M3
-1 2 -2	0 1 1	4 1 -1	-4 0 2	M4
-1 -2 2	0 -1 -1	4 -1 1	-4 0 -2	M5
2 1 -2	1 0 1	1 -4 -1	0 4 2	M6
-2 -1 -2	-1 0 1	-1 4 -1	0 -4 2	M7
2 -2 1	-1 -1 0	1 -1 -4	-2 0 4	M8
-2 -2 -1	1 -1 0	-1 -1 4	2 0 -4	M9
-1 2 2	0 -1 1	4 1 1	-4 -2 0	M10
-1 -2 -2	0 1 -1	4 -1 -1	-4 2 0	M11
1 -2 -2	0 1 -1	4 1 1	-4 0 -2	M12
-1 2 -2	0 -1 -1	-4 -1 1	4 0 -2	M13
-1 -2 2	0 1 1	-4 1 -1	4 0 2	M14
2 1 -2	-1 0 -1	-1 4 1	0 -4 -2	M15
-2 -1 -2	1 0 -1	1 -4 1	0 4 -2	M16
2 -2 1	1 1 0	-1 1 4	2 0 -4	M17
-2 -2 -1	-1 1 0	1 1 -4	-2 0 4	M18
-1 2 2	0 1 -1	-4 -1 -1	4 2 0	M19
-1 -2 -2	0 -1 1	-4 1 1	4 -2 0	M20
2 1 2	-1 0 1	1 -4 1	-2 4 0	M21
2 2 1	1 -1 0	1 1 -4	0 -2 4	M22
-2 -1 2	1 0 1	-1 4 1	2 -4 0	M23
-2 2 -1	-1 -1 0	-1 1 4	0 -2 -4	M24
2 -1 -2	-1 0 -1	1 4 -1	-2 -4 0	M25
-2 -2 1	-1 1 0	-1 -1 -4	0 2 4	M26
-2 1 -2	1 0 -1	-1 -4 -1	2 4 0	M27
2 -2 -1	1 1 0	1 -1 4	0 2 -4	M28
2 1 2	1 0 -1	-1 4 -1	2 -4 0	M29
2 2 1	-1 1 0	-1 -1 4	0 2 -4	M30
-2 -1 2	-1 0 -1	1 -4 -1	-2 4 0	M31
-2 2 -1	1 1 0	1 -1 -4	0 2 4	M32
2 -1 -2	1 0 1	-1 -4 1	2 4 0	M33
-2 -2 1	1 -1 0	1 1 4	0 -2 -4	M34
-2 1 -2	-1 0 1	1 4 1	-2 -4 0	M35
2 -2 -1	-1 -1 0	-1 1 -4	0 -2 4	M36
-2 1 2	-1 0 -1	-1 -4 1	0 4 -2	M37
2 -1 2	1 0 -1	1 4 1	0 -4 -2	M38
1 -2 2	0 1 1	-4 -1 1	4 2 0	M39
1 2 -2	0 -1 -1	-4 1 -1	4 -2 0	M40
2 2 -1	-1 1 0	1 1 4	-2 0 -4	M41
-2 2 1	1 1 0	-1 1 -4	2 0 4	M42
-2 1 2	1 0 1	1 4 -1	0 -4 2	M43
2 -1 2	-1 0 1	-1 -4 -1	0 4 2	M44
1 -2 2	0 -1 -1	4 1 -1	-4 -2 0	M45
1 2 -2	0 1 1	4 -1 1	-4 2 0	M46
2 2 -1	1 -1 0	-1 -1 -4	2 0 4	M47
-2 2 1	-1 -1 0	1 -1 4	-2 0 -4	M48

Table 18: F - lattice [1 2 2]

[*uvw*]	*hkl A*	*hkl B*	*hkl C*	M No.
1 2 2	0 -2 2	4 -2 0	-4 0 2	M1
1 2 2	0 2 -2	-4 2 0	4 0 -2	M2
1 -2 -2	0 2 -2	4 2 0	-4 0 -2	M3
-1 2 -2	0 -2 -2	-4 -2 0	4 0 -2	M4
-1 -2 2	0 2 2	-4 2 0	4 0 2	M5
2 1 -2	-2 0 -2	-2 4 0	0 -4 -2	M6
-2 -1 -2	2 0 -2	2 -4 0	0 4 -2	M7
2 -2 1	2 2 0	0 2 4	2 0 -4	M8
-2 -2 -1	-2 2 0	0 2 -4	-2 0 4	M9
-1 2 2	0 2 -2	-4 0 -2	4 2 0	M10
-1 -2 -2	0 -2 2	-4 0 2	4 -2 0	M11
1 -2 -2	0 -2 2	-4 -2 0	4 0 2	M12
-1 2 -2	0 2 2	4 2 0	-4 0 2	M13
-1 -2 2	0 -2 -2	4 -2 0	-4 0 -2	M14
2 1 -2	2 0 2	2 -4 0	0 4 2	M15
-2 -1 -2	-2 0 2	-2 4 0	0 -4 2	M16
2 -2 1	-2 -2 0	0 -2 -4	-2 0 4	M17
-2 -2 -1	2 -2 0	0 -2 4	2 0 -4	M18
-1 2 2	0 -2 2	4 0 2	-4 -2 0	M19
-1 -2 -2	0 2 -2	4 0 -2	-4 2 0	M20
2 1 2	2 0 -2	0 4 -2	2 -4 0	M21
2 2 1	-2 2 0	-2 0 4	0 2 -4	M22
-2 -1 2	-2 0 -2	0 -4 -2	-2 4 0	M23
-2 2 -1	2 2 0	2 0 -4	0 2 4	M24
2 -1 -2	2 0 2	0 -4 2	2 4 0	M25
-2 -2 1	2 -2 0	2 0 4	0 -2 -4	M26
-2 1 -2	-2 0 2	0 4 2	-2 -4 0	M27
2 -2 -1	-2 -2 0	-2 0 -4	0 -2 4	M28
2 1 2	-2 0 2	0 -4 2	-2 4 0	M29
2 2 1	2 -2 0	2 0 -4	0 -2 4	M30
-2 -1 2	2 0 2	0 4 2	2 -4 0	M31
-2 2 -1	-2 -2 0	-2 0 4	0 -2 -4	M32
2 -1 -2	-2 0 -2	0 4 -2	-2 -4 0	M33
-2 -2 1	-2 2 0	-2 0 -4	0 2 4	M34
-2 1 -2	2 0 -2	0 -4 -2	2 4 0	M35
2 -2 -1	2 2 0	2 0 4	0 2 -4	M36
-2 1 2	2 0 2	2 4 0	0 -4 2	M37
2 -1 2	-2 0 2	-2 -4 0	0 4 2	M38
1 -2 2	0 -2 -2	4 0 -2	-4 -2 0	M39
1 2 -2	0 2 2	4 0 2	-4 2 0	M40
2 2 -1	2 -2 0	0 -2 -4	2 0 4	M41
-2 2 1	-2 -2 0	0 -2 4	-2 0 -4	M42
-2 1 2	-2 0 -2	-2 -4 0	0 4 -2	M43
2 -1 2	2 0 -2	2 4 0	0 -4 -2	M44
1 -2 2	0 2 2	-4 0 2	4 2 0	M45
1 2 -2	0 -2 -2	-4 0 -2	4 -2 0	M46
2 2 -1	-2 2 0	0 2 4	-2 0 -4	M47
-2 2 1	2 2 0	0 2 -4	2 0 4	M48

Table 19: P - lattice [0 1 3]

[*uvw*]	*hkl A*	*hkl B*	*hkl C*	M No.
0 1 3	1 0 0	0 3 -1	1 -3 1	M1
0 1 3	-1 0 0	0 -3 1	-1 3 -1	M2
0 -1 -3	1 0 0	0 -3 1	1 3 -1	M3
0 1 -3	-1 0 0	0 3 1	-1 -3 -1	M4
0 -1 3	-1 0 0	0 -3 -1	-1 3 1	M5
1 0 -3	0 1 0	3 0 1	-3 1 -1	M6
-1 0 -3	0 -1 0	-3 0 1	3 -1 -1	M7
3 -1 0	0 0 1	-1 -3 0	1 3 1	M8
-3 -1 0	0 0 -1	1 -3 0	-1 3 -1	M9
0 3 1	-1 0 0	0 -1 3	-1 1 -3	M10
0 -3 -1	-1 0 0	0 1 -3	-1 -1 3	M11
0 -1 -3	-1 0 0	0 3 -1	-1 -3 1	M12
0 1 -3	1 0 0	0 -3 -1	1 3 1	M13
0 -1 3	1 0 0	0 3 1	1 -3 -1	M14
1 0 -3	0 -1 0	-3 0 -1	3 -1 1	M15
-1 0 -3	0 1 0	3 0 -1	-3 1 1	M16
3 -1 0	0 0 -1	1 3 0	-1 -3 -1	M17
-3 -1 0	0 0 1	-1 3 0	1 -3 1	M18
0 3 1	1 0 0	0 1 -3	1 -1 3	M19
0 -3 -1	1 0 0	0 -1 3	1 1 -3	M20
3 0 1	0 1 0	-1 0 3	1 1 -3	M21
1 3 0	0 0 1	3 -1 0	-3 1 1	M22
-3 0 1	0 -1 0	1 0 3	-1 -1 -3	M23
-1 3 0	0 0 -1	-3 -1 0	3 1 -1	M24
3 0 -1	0 -1 0	-1 0 -3	1 -1 3	M25
-1 -3 0	0 0 1	-3 1 0	3 -1 1	M26
-3 0 -1	0 1 0	1 0 -3	-1 1 3	M27
1 -3 0	0 0 -1	3 1 0	-3 -1 -1	M28
3 0 1	0 -1 0	1 0 -3	-1 -1 3	M29
1 3 0	0 0 -1	-3 1 0	3 -1 -1	M30
-3 0 1	0 1 0	-1 0 -3	1 1 3	M31
-1 3 0	0 0 1	3 1 0	-3 -1 1	M32
3 0 -1	0 1 0	1 0 3	-1 1 -3	M33
-1 -3 0	0 0 -1	3 -1 0	-3 1 -1	M34
-3 0 -1	0 -1 0	-1 0 3	1 -1 -3	M35
1 -3 0	0 0 1	-3 -1 0	3 1 1	M36
-1 0 3	0 1 0	-3 0 -1	3 1 1	M37
1 0 3	0 -1 0	3 0 -1	-3 -1 1	M38
0 -3 1	1 0 0	0 1 3	1 -1 -3	M39
0 3 -1	1 0 0	0 -1 -3	1 1 3	M40
3 1 0	0 0 -1	-1 3 0	1 -3 -1	M41
-3 1 0	0 0 1	1 3 0	-1 -3 1	M42
-1 0 3	0 -1 0	3 0 1	-3 -1 -1	M43
1 0 3	0 1 0	-3 0 1	3 1 -1	M44
0 -3 1	-1 0 0	0 -1 -3	-1 1 3	M45
0 3 -1	-1 0 0	0 1 3	-1 -1 -3	M46
3 1 0	0 0 1	1 -3 0	-1 3 1	M47
-3 1 0	0 0 -1	-1 -3 0	1 3 -1	M48

Table 20: I - lattice [0 1 3]

[*uvw*]	*hkl A*	*hkl B*	*hkl C*	M No.
0 1 3	2 0 0	0 3 -1	2 -3 1	M1
0 1 3	-2 0 0	0 -3 1	-2 3 -1	M2
0 -1 -3	2 0 0	0 -3 1	2 3 -1	M3
0 1 -3	-2 0 0	0 3 1	-2 -3 -1	M4
0 -1 3	-2 0 0	0 -3 -1	-2 3 1	M5
1 0 -3	0 2 0	3 0 1	-3 2 -1	M6
-1 0 -3	0 -2 0	-3 0 1	3 -2 -1	M7
3 -1 0	0 0 2	-1 -3 0	1 3 2	M8
-3 -1 0	0 0 -2	1 -3 0	-1 3 -2	M9
0 3 1	-2 0 0	0 -1 3	-2 1 -3	M10
0 -3 -1	-2 0 0	0 1 -3	-2 -1 3	M11
0 -1 -3	-2 0 0	0 3 -1	-2 -3 1	M12
0 1 -3	2 0 0	0 -3 -1	2 3 1	M13
0 -1 3	2 0 0	0 3 1	2 -3 -1	M14
1 0 -3	0 -2 0	-3 0 -1	3 -2 1	M15
-1 0 -3	0 2 0	3 0 -1	-3 2 1	M16
3 -1 0	0 0 -2	1 3 0	-1 -3 -2	M17
-3 -1 0	0 0 2	-1 3 0	1 -3 2	M18
0 3 1	2 0 0	0 1 -3	2 -1 3	M19
0 -3 -1	2 0 0	0 -1 3	2 1 -3	M20
3 0 1	0 2 0	-1 0 3	1 2 -3	M21
1 3 0	0 0 2	3 -1 0	-3 1 2	M22
-3 0 1	0 -2 0	1 0 3	-1 -2 -3	M23
-1 3 0	0 0 -2	-3 -1 0	3 1 -2	M24
3 0 -1	0 -2 0	-1 0 -3	1 -2 3	M25
-1 -3 0	0 0 2	-3 1 0	3 -1 2	M26
-3 0 -1	0 2 0	1 0 -3	-1 2 3	M27
1 -3 0	0 0 -2	3 1 0	-3 -1 -2	M28
3 0 1	0 -2 0	1 0 -3	-1 -2 3	M29
1 3 0	0 0 -2	-3 1 0	3 -1 -2	M30
-3 0 1	0 2 0	-1 0 -3	1 2 3	M31
-1 3 0	0 0 2	3 1 0	-3 -1 2	M32
3 0 -1	0 2 0	1 0 3	-1 2 -3	M33
-1 -3 0	0 0 -2	3 -1 0	-3 1 -2	M34
-3 0 -1	0 -2 0	-1 0 3	1 -2 -3	M35
1 -3 0	0 0 2	-3 -1 0	3 1 2	M36
-1 0 3	0 2 0	-3 0 -1	3 2 1	M37
1 0 3	0 -2 0	3 0 -1	-3 -2 1	M38
0 -3 1	2 0 0	0 1 3	2 -1 -3	M39
0 3 -1	2 0 0	0 -1 -3	2 1 3	M40
3 1 0	0 0 -2	-1 3 0	1 -3 -2	M41
-3 1 0	0 0 2	1 3 0	-1 -3 2	M42
-1 0 3	0 -2 0	3 0 1	-3 -2 -1	M43
1 0 3	0 2 0	-3 0 1	3 2 -1	M44
0 -3 1	-2 0 0	0 -1 -3	-2 1 3	M45
0 3 -1	-2 0 0	0 1 3	-2 -1 -3	M46
3 1 0	0 0 2	1 -3 0	-1 3 2	M47
-3 1 0	0 0 -2	-1 -3 0	1 3 -2	M48

Table 21: F - lattice [0 1 3]

[*uvw*]	*hkl A*	*hkl B*	*hkl C*	M No.
0 1 3	2 0 0	1 3 -1	1 -3 1	M1
0 1 3	-2 0 0	-1 -3 1	-1 3 -1	M2
0 -1 -3	2 0 0	1 -3 1	1 3 -1	M3
0 1 -3	-2 0 0	-1 3 1	-1 -3 -1	M4
0 -1 3	-2 0 0	-1 -3 -1	-1 3 1	M5
1 0 -3	0 2 0	3 1 1	-3 1 -1	M6
-1 0 -3	0 -2 0	-3 -1 1	3 -1 -1	M7
3 -1 0	0 0 2	-1 -3 1	1 3 1	M8
-3 -1 0	0 0 -2	1 -3 -1	-1 3 -1	M9
0 3 1	-2 0 0	-1 -1 3	-1 1 -3	M10
0 -3 -1	-2 0 0	-1 1 -3	-1 -1 3	M11
0 -1 -3	-2 0 0	-1 3 -1	-1 -3 1	M12
0 1 -3	2 0 0	1 -3 -1	1 3 1	M13
0 -1 3	2 0 0	1 3 1	1 -3 -1	M14
1 0 -3	0 -2 0	-3 -1 -1	3 -1 1	M15
-1 0 -3	0 2 0	3 1 -1	-3 1 1	M16
3 -1 0	0 0 -2	1 3 -1	-1 -3 -1	M17
-3 -1 0	0 0 2	-1 3 1	1 -3 1	M18
0 3 1	2 0 0	1 1 -3	1 -1 3	M19
0 -3 -1	2 0 0	1 -1 3	1 1 -3	M20
3 0 1	0 2 0	-1 1 3	1 1 -3	M21
1 3 0	0 0 2	3 -1 1	-3 1 1	M22
-3 0 1	0 -2 0	1 -1 3	-1 -1 -3	M23
-1 3 0	0 0 -2	-3 -1 -1	3 1 -1	M24
3 0 -1	0 -2 0	-1 -1 -3	1 -1 3	M25
-1 -3 0	0 0 2	-3 1 1	3 -1 1	M26
-3 0 -1	0 2 0	1 1 -3	-1 1 3	M27
1 -3 0	0 0 -2	3 1 -1	-3 -1 -1	M28
3 0 1	0 -2 0	1 -1 -3	-1 -1 3	M29
1 3 0	0 0 -2	-3 1 -1	3 -1 -1	M30
-3 0 1	0 2 0	-1 1 -3	1 1 3	M31
-1 3 0	0 0 2	3 1 1	-3 -1 1	M32
3 0 -1	0 2 0	1 1 3	-1 1 -3	M33
-1 -3 0	0 0 -2	3 -1 -1	-3 1 -1	M34
-3 0 -1	0 -2 0	-1 -1 3	1 -1 -3	M35
1 -3 0	0 0 2	-3 -1 1	3 1 1	M36
-1 0 3	0 2 0	-3 1 -1	3 1 1	M37
1 0 3	0 -2 0	3 -1 -1	-3 -1 1	M38
0 -3 1	2 0 0	1 1 3	1 -1 -3	M39
0 3 -1	2 0 0	1 -1 -3	1 1 3	M40
3 1 0	0 0 -2	-1 3 -1	1 -3 -1	M41
-3 1 0	0 0 2	1 3 1	-1 -3 1	M42
-1 0 3	0 -2 0	3 -1 1	-3 -1 -1	M43
1 0 3	0 2 0	-3 1 1	3 1 -1	M44
0 -3 1	-2 0 0	-1 -1 -3	-1 1 3	M45
0 3 -1	-2 0 0	-1 1 3	-1 -1 -3	M46
3 1 0	0 0 2	1 -3 1	-1 3 1	M47
-3 1 0	0 0 -2	-1 -3 -1	1 3 -1	M48

Table 22: P - lattice [1 1 3]

[*uvw*]	*hkl A*	*hkl B*	*hkl C*	M No.
1 1 3	1 -1 0	2 1 -1	-1 -2 1	M1
1 1 3	-1 1 0	-2 -1 1	1 2 -1	M2
1 -1 -3	1 1 0	2 -1 1	-1 2 -1	M3
-1 1 -3	-1 -1 0	-2 1 1	1 -2 -1	M4
-1 -1 3	-1 1 0	-2 -1 -1	1 2 1	M5
1 1 -3	-1 1 0	1 2 1	-2 -1 -1	M6
-1 -1 -3	1 -1 0	-1 -2 1	2 1 -1	M7
3 -1 1	0 1 1	-1 -1 2	1 2 -1	M8
-3 -1 -1	0 1 -1	1 -1 -2	-1 2 1	M9
-1 3 1	-1 0 -1	-2 -1 1	1 1 -2	M10
-1 -3 -1	-1 0 1	-2 1 -1	1 -1 2	M11
1 -1 -3	-1 -1 0	-2 1 -1	1 -2 1	M12
-1 1 -3	1 1 0	2 -1 -1	-1 2 1	M13
-1 -1 3	1 -1 0	2 1 1	-1 -2 -1	M14
1 1 -3	1 -1 0	-1 -2 -1	2 1 1	M15
-1 -1 -3	-1 1 0	1 2 -1	-2 -1 1	M16
3 -1 1	0 -1 -1	1 1 -2	-1 -2 1	M17
-3 -1 -1	0 -1 1	-1 1 2	1 -2 -1	M18
-1 3 1	1 0 1	2 1 -1	-1 -1 2	M19
-1 -3 -1	1 0 -1	2 -1 1	-1 1 -2	M20
3 1 1	0 1 -1	-1 2 1	1 -1 -2	M21
1 3 1	-1 0 1	1 -1 2	-2 1 -1	M22
-3 -1 1	0 -1 -1	1 -2 1	-1 1 -2	M23
-1 3 -1	1 0 -1	-1 -1 -2	2 1 1	M24
3 -1 -1	0 -1 1	-1 -2 -1	1 1 2	M25
-1 -3 1	1 0 1	-1 1 2	2 -1 -1	M26
-3 1 -1	0 1 1	1 2 -1	-1 -1 2	M27
1 -3 -1	-1 0 -1	1 1 -2	-2 -1 1	M28
3 1 1	0 -1 1	1 -2 -1	-1 1 2	M29
1 3 1	1 0 -1	-1 1 -2	2 -1 1	M30
-3 -1 1	0 1 1	-1 2 -1	1 -1 2	M31
-1 3 -1	-1 0 1	1 1 2	-2 -1 -1	M32
3 -1 -1	0 1 -1	1 2 1	-1 -1 -2	M33
-1 -3 1	-1 0 -1	1 -1 -2	-2 1 1	M34
-3 1 -1	0 -1 -1	-1 -2 1	1 1 -2	M35
1 -3 -1	1 0 1	-1 -1 2	2 1 -1	M36
-1 1 3	1 1 0	-1 2 -1	2 -1 1	M37
1 -1 3	-1 -1 0	1 -2 -1	-2 1 1	M38
1 -3 1	1 0 -1	2 1 1	-1 -1 -2	M39
1 3 -1	1 0 1	2 -1 -1	-1 1 2	M40
3 1 -1	0 -1 -1	-1 1 -2	1 -2 1	M41
-3 1 1	0 -1 1	1 1 2	-1 -2 -1	M42
-1 1 3	-1 -1 0	1 -2 1	-2 1 -1	M43
1 -1 3	1 1 0	-1 2 1	2 -1 -1	M44
1 -3 1	-1 0 1	-2 -1 -1	1 1 2	M45
1 3 -1	-1 0 -1	-2 1 1	1 -1 -2	M46
3 1 -1	0 1 1	1 -1 2	-1 2 -1	M47
-3 1 1	0 1 -1	-1 -1 -2	1 2 1	M48

Table 23: I - lattice [1 1 3]

[*uvw*]	*hkl A*	*hkl B*	*hkl C*	M No.
1 1 3	1 -1 0	2 1 -1	-1 -2 1	M1
1 1 3	-1 1 0	-2 -1 1	1 2 -1	M2
1 -1 -3	1 1 0	2 -1 1	-1 2 -1	M3
-1 1 -3	-1 -1 0	-2 1 1	1 -2 -1	M4
-1 -1 3	-1 1 0	-2 -1 -1	1 2 1	M5
1 1 -3	-1 1 0	1 2 1	-2 -1 -1	M6
-1 -1 -3	1 -1 0	-1 -2 1	2 1 -1	M7
3 -1 1	0 1 1	-1 -1 2	1 2 -1	M8
-3 -1 -1	0 1 -1	1 -1 -2	-1 2 1	M9
-1 3 1	-1 0 -1	-2 -1 1	1 1 -2	M10
-1 -3 -1	-1 0 1	-2 1 -1	1 -1 2	M11
1 -1 -3	-1 -1 0	-2 1 -1	1 -2 1	M12
-1 1 -3	1 1 0	2 -1 -1	-1 2 1	M13
-1 -1 3	1 -1 0	2 1 1	-1 -2 -1	M14
1 1 -3	1 -1 0	-1 -2 -1	2 1 1	M15
-1 -1 -3	-1 1 0	1 2 -1	-2 -1 1	M16
3 -1 1	0 -1 -1	1 1 -2	-1 -2 1	M17
-3 -1 -1	0 -1 1	-1 1 2	1 -2 -1	M18
-1 3 1	1 0 1	2 1 -1	-1 -1 2	M19
-1 -3 -1	1 0 -1	2 -1 1	-1 1 -2	M20
3 1 1	0 1 -1	-1 2 1	1 -1 -2	M21
1 3 1	-1 0 1	1 -1 2	-2 1 -1	M22
-3 -1 1	0 -1 -1	1 -2 1	-1 1 -2	M23
-1 3 -1	1 0 -1	-1 -1 -2	2 1 1	M24
3 -1 -1	0 -1 1	-1 -2 -1	1 1 2	M25
-1 -3 1	1 0 1	-1 1 2	2 -1 -1	M26
-3 1 -1	0 1 1	1 2 -1	-1 -1 2	M27
1 -3 -1	-1 0 -1	1 1 -2	-2 -1 1	M28
3 1 1	0 -1 1	1 -2 -1	-1 1 2	M29
1 3 1	1 0 -1	-1 1 -2	2 -1 1	M30
-3 -1 1	0 1 1	-1 2 -1	1 -1 2	M31
-1 3 -1	-1 0 1	1 1 2	-2 -1 -1	M32
3 -1 -1	0 1 -1	1 2 1	-1 -1 -2	M33
-1 -3 1	-1 0 -1	1 -1 -2	-2 1 1	M34
-3 1 -1	0 -1 -1	-1 -2 1	1 1 -2	M35
1 -3 -1	1 0 1	-1 -1 2	2 1 -1	M36
-1 1 3	1 1 0	-1 2 -1	2 -1 1	M37
1 -1 3	-1 -1 0	1 -2 -1	-2 1 1	M38
1 -3 1	1 0 -1	2 1 1	-1 -1 -2	M39
1 3 -1	1 0 1	2 -1 -1	-1 1 2	M40
3 1 -1	0 -1 -1	-1 1 -2	1 -2 1	M41
-3 1 1	0 -1 1	1 1 2	-1 -2 -1	M42
-1 1 3	-1 -1 0	1 -2 1	-2 1 -1	M43
1 -1 3	1 1 0	-1 2 1	2 -1 -1	M44
1 -3 1	-1 0 1	-2 -1 -1	1 1 2	M45
1 3 -1	-1 0 -1	-2 1 1	1 -1 -2	M46
3 1 -1	0 1 1	1 -1 2	-1 2 -1	M47
-3 1 1	0 1 -1	-1 -1 -2	1 2 1	M48

https://doi.org/10.1107/S1600576726003031

Table 24: F - lattice [1 1 3]

[*uvw*]	*hkl A*	*hkl B*	*hkl C*	M No.
1 1 3	2 -2 0	4 2 -2	-2 -4 2	M1
1 1 3	-2 2 0	-4 -2 2	2 4 -2	M2
1 -1 -3	2 2 0	4 -2 2	-2 4 -2	M3
-1 1 -3	-2 -2 0	-4 2 2	2 -4 -2	M4
-1 -1 3	-2 2 0	-4 -2 -2	2 4 2	M5
1 1 -3	-2 2 0	2 4 2	-4 -2 -2	M6
-1 -1 -3	2 -2 0	-2 -4 2	4 2 -2	M7
3 -1 1	0 2 2	-2 -2 4	2 4 -2	M8
-3 -1 -1	0 2 -2	2 -2 -4	-2 4 2	M9
-1 3 1	-2 0 -2	-4 -2 2	2 2 -4	M10
-1 -3 -1	-2 0 2	-4 2 -2	2 -2 4	M11
1 -1 -3	-2 -2 0	-4 2 -2	2 -4 2	M12
-1 1 -3	2 2 0	4 -2 -2	-2 4 2	M13
-1 -1 3	2 -2 0	4 2 2	-2 -4 -2	M14
1 1 -3	2 -2 0	-2 -4 -2	4 2 2	M15
-1 -1 -3	-2 2 0	2 4 -2	-4 -2 2	M16
3 -1 1	0 -2 -2	2 2 -4	-2 -4 2	M17
-3 -1 -1	0 -2 2	-2 2 4	2 -4 -2	M18
-1 3 1	2 0 2	4 2 -2	-2 -2 4	M19
-1 -3 -1	2 0 -2	4 -2 2	-2 2 -4	M20
3 1 1	0 2 -2	-2 4 2	2 -2 -4	M21
1 3 1	-2 0 2	2 -2 4	-4 2 -2	M22
-3 -1 1	0 -2 -2	2 -4 2	-2 2 -4	M23
-1 3 -1	2 0 -2	-2 -2 -4	4 2 2	M24
3 -1 -1	0 -2 2	-2 -4 -2	2 2 4	M25
-1 -3 1	2 0 2	-2 2 4	4 -2 -2	M26
-3 1 -1	0 2 2	2 4 -2	-2 -2 4	M27
1 -3 -1	-2 0 -2	2 2 -4	-4 -2 2	M28
3 1 1	0 -2 2	2 -4 -2	-2 2 4	M29
1 3 1	2 0 -2	-2 2 -4	4 -2 2	M30
-3 -1 1	0 2 2	-2 4 -2	2 -2 4	M31
-1 3 -1	-2 0 2	2 2 4	-4 -2 -2	M32
3 -1 -1	0 2 -2	2 4 2	-2 -2 -4	M33
-1 -3 1	-2 0 -2	2 -2 -4	-4 2 2	M34
-3 1 -1	0 -2 -2	-2 -4 2	2 2 -4	M35
1 -3 -1	2 0 2	-2 -2 4	4 2 -2	M36
-1 1 3	2 2 0	-2 4 -2	4 -2 2	M37
1 -1 3	-2 -2 0	2 -4 -2	-4 2 2	M38
1 -3 1	2 0 -2	4 2 2	-2 -2 -4	M39
1 3 -1	2 0 2	4 -2 -2	-2 2 4	M40
3 1 -1	0 -2 -2	-2 2 -4	2 -4 2	M41
-3 1 1	0 -2 2	2 2 4	-2 -4 -2	M42
-1 1 3	-2 -2 0	2 -4 2	-4 2 -2	M43
1 -1 3	2 2 0	-2 4 2	4 -2 -2	M44
1 -3 1	-2 0 2	-4 -2 -2	2 2 4	M45
1 3 -1	-2 0 -2	-4 2 2	2 -2 -4	M46
3 1 -1	0 2 2	2 -2 4	-2 4 -2	M47
-3 1 1	0 2 -2	-2 -2 -4	2 4 2	M48

Table 25: P - lattice [0 2 3]

[*uvw*]	*hkl A*	*hkl B*	*hkl C*	M No.
0 2 3	1 0 0	0 3 -2	1 -3 2	M1
0 2 3	-1 0 0	0 -3 2	-1 3 -2	M2
0 -2 -3	1 0 0	0 -3 2	1 3 -2	M3
0 2 -3	-1 0 0	0 3 2	-1 -3 -2	M4
0 -2 3	-1 0 0	0 -3 -2	-1 3 2	M5
2 0 -3	0 1 0	3 0 2	-3 1 -2	M6
-2 0 -3	0 -1 0	-3 0 2	3 -1 -2	M7
3 -2 0	0 0 1	-2 -3 0	2 3 1	M8
-3 -2 0	0 0 -1	2 -3 0	-2 3 -1	M9
0 3 2	-1 0 0	0 -2 3	-1 2 -3	M10
0 -3 -2	-1 0 0	0 2 -3	-1 -2 3	M11
0 -2 -3	-1 0 0	0 3 -2	-1 -3 2	M12
0 2 -3	1 0 0	0 -3 -2	1 3 2	M13
0 -2 3	1 0 0	0 3 2	1 -3 -2	M14
2 0 -3	0 -1 0	-3 0 -2	3 -1 2	M15
-2 0 -3	0 1 0	3 0 -2	-3 1 2	M16
3 -2 0	0 0 -1	2 3 0	-2 -3 -1	M17
-3 -2 0	0 0 1	-2 3 0	2 -3 1	M18
0 3 2	1 0 0	0 2 -3	1 -2 3	M19
0 -3 -2	1 0 0	0 -2 3	1 2 -3	M20
3 0 2	0 1 0	-2 0 3	2 1 -3	M21
2 3 0	0 0 1	3 -2 0	-3 2 1	M22
-3 0 2	0 -1 0	2 0 3	-2 -1 -3	M23
-2 3 0	0 0 -1	-3 -2 0	3 2 -1	M24
3 0 -2	0 -1 0	-2 0 -3	2 -1 3	M25
-2 -3 0	0 0 1	-3 2 0	3 -2 1	M26
-3 0 -2	0 1 0	2 0 -3	-2 1 3	M27
2 -3 0	0 0 -1	3 2 0	-3 -2 -1	M28
3 0 2	0 -1 0	2 0 -3	-2 -1 3	M29
2 3 0	0 0 -1	-3 2 0	3 -2 -1	M30
-3 0 2	0 1 0	-2 0 -3	2 1 3	M31
-2 3 0	0 0 1	3 2 0	-3 -2 1	M32
3 0 -2	0 1 0	2 0 3	-2 1 -3	M33
-2 -3 0	0 0 -1	3 -2 0	-3 2 -1	M34
-3 0 -2	0 -1 0	-2 0 3	2 -1 -3	M35
2 -3 0	0 0 1	-3 -2 0	3 2 1	M36
-2 0 3	0 1 0	-3 0 -2	3 1 2	M37
2 0 3	0 -1 0	3 0 -2	-3 -1 2	M38
0 -3 2	1 0 0	0 2 3	1 -2 -3	M39
0 3 -2	1 0 0	0 -2 -3	1 2 3	M40
3 2 0	0 0 -1	-2 3 0	2 -3 -1	M41
-3 2 0	0 0 1	2 3 0	-2 -3 1	M42
-2 0 3	0 -1 0	3 0 2	-3 -1 -2	M43
2 0 3	0 1 0	-3 0 2	3 1 -2	M44
0 -3 2	-1 0 0	0 -2 -3	-1 2 3	M45
0 3 -2	-1 0 0	0 2 3	-1 -2 -3	M46
3 2 0	0 0 1	2 -3 0	-2 3 1	M47
-3 2 0	0 0 -1	-2 -3 0	2 3 -1	M48

Table 26: I - lattice [0 2 3]

[*uvw*]	*hkl A*	*hkl B*	*hkl C*	M No.
0 2 3	2 0 0	1 3 -2	1 -3 2	M1
0 2 3	-2 0 0	-1 -3 2	-1 3 -2	M2
0 -2 -3	2 0 0	1 -3 2	1 3 -2	M3
0 2 -3	-2 0 0	-1 3 2	-1 -3 -2	M4
0 -2 3	-2 0 0	-1 -3 -2	-1 3 2	M5
2 0 -3	0 2 0	3 1 2	-3 1 -2	M6
-2 0 -3	0 -2 0	-3 -1 2	3 -1 -2	M7
3 -2 0	0 0 2	-2 -3 1	2 3 1	M8
-3 -2 0	0 0 -2	2 -3 -1	-2 3 -1	M9
0 3 2	-2 0 0	-1 -2 3	-1 2 -3	M10
0 -3 -2	-2 0 0	-1 2 -3	-1 -2 3	M11
0 -2 -3	-2 0 0	-1 3 -2	-1 -3 2	M12
0 2 -3	2 0 0	1 -3 -2	1 3 2	M13
0 -2 3	2 0 0	1 3 2	1 -3 -2	M14
2 0 -3	0 -2 0	-3 -1 -2	3 -1 2	M15
-2 0 -3	0 2 0	3 1 -2	-3 1 2	M16
3 -2 0	0 0 -2	2 3 -1	-2 -3 -1	M17
-3 -2 0	0 0 2	-2 3 1	2 -3 1	M18
0 3 2	2 0 0	1 2 -3	1 -2 3	M19
0 -3 -2	2 0 0	1 -2 3	1 2 -3	M20
3 0 2	0 2 0	-2 1 3	2 1 -3	M21
2 3 0	0 0 2	3 -2 1	-3 2 1	M22
-3 0 2	0 -2 0	2 -1 3	-2 -1 -3	M23
-2 3 0	0 0 -2	-3 -2 -1	3 2 -1	M24
3 0 -2	0 -2 0	-2 -1 -3	2 -1 3	M25
-2 -3 0	0 0 2	-3 2 1	3 -2 1	M26
-3 0 -2	0 2 0	2 1 -3	-2 1 3	M27
2 -3 0	0 0 -2	3 2 -1	-3 -2 -1	M28
3 0 2	0 -2 0	2 -1 -3	-2 -1 3	M29
2 3 0	0 0 -2	-3 2 -1	3 -2 -1	M30
-3 0 2	0 2 0	-2 1 -3	2 1 3	M31
-2 3 0	0 0 2	3 2 1	-3 -2 1	M32
3 0 -2	0 2 0	2 1 3	-2 1 -3	M33
-2 -3 0	0 0 -2	3 -2 -1	-3 2 -1	M34
-3 0 -2	0 -2 0	-2 -1 3	2 -1 -3	M35
2 -3 0	0 0 2	-3 -2 1	3 2 1	M36
-2 0 3	0 2 0	-3 1 -2	3 1 2	M37
2 0 3	0 -2 0	3 -1 -2	-3 -1 2	M38
0 -3 2	2 0 0	1 2 3	1 -2 -3	M39
0 3 -2	2 0 0	1 -2 -3	1 2 3	M40
3 2 0	0 0 -2	-2 3 -1	2 -3 -1	M41
-3 2 0	0 0 2	2 3 1	-2 -3 1	M42
-2 0 3	0 -2 0	3 -1 2	-3 -1 -2	M43
2 0 3	0 2 0	-3 1 2	3 1 -2	M44
0 -3 2	-2 0 0	-1 -2 -3	-1 2 3	M45
0 3 -2	-2 0 0	-1 2 3	-1 -2 -3	M46
3 2 0	0 0 2	2 -3 1	-2 3 1	M47
-3 2 0	0 0 -2	-2 -3 -1	2 3 -1	M48

Table 27: F - lattice [0 2 3]

[*uvw*]	*hkl A*	*hkl B*	*hkl C*	M No.
0 2 3	2 0 0	0 6 -4	2 -6 4	M1
0 2 3	-2 0 0	0 -6 4	-2 6 -4	M2
0 -2 -3	2 0 0	0 -6 4	2 6 -4	M3
0 2 -3	-2 0 0	0 6 4	-2 -6 -4	M4
0 -2 3	-2 0 0	0 -6 -4	-2 6 4	M5
2 0 -3	0 2 0	6 0 4	-6 2 -4	M6
-2 0 -3	0 -2 0	-6 0 4	6 -2 -4	M7
3 -2 0	0 0 2	-4 -6 0	4 6 2	M8
-3 -2 0	0 0 -2	4 -6 0	-4 6 -2	M9
0 3 2	-2 0 0	0 -4 6	-2 4 -6	M10
0 -3 -2	-2 0 0	0 4 -6	-2 -4 6	M11
0 -2 -3	-2 0 0	0 6 -4	-2 -6 4	M12
0 2 -3	2 0 0	0 -6 -4	2 6 4	M13
0 -2 3	2 0 0	0 6 4	2 -6 -4	M14
2 0 -3	0 -2 0	-6 0 -4	6 -2 4	M15
-2 0 -3	0 2 0	6 0 -4	-6 2 4	M16
3 -2 0	0 0 -2	4 6 0	-4 -6 -2	M17
-3 -2 0	0 0 2	-4 6 0	4 -6 2	M18
0 3 2	2 0 0	0 4 -6	2 -4 6	M19
0 -3 -2	2 0 0	0 -4 6	2 4 -6	M20
3 0 2	0 2 0	-4 0 6	4 2 -6	M21
2 3 0	0 0 2	6 -4 0	-6 4 2	M22
-3 0 2	0 -2 0	4 0 6	-4 -2 -6	M23
-2 3 0	0 0 -2	-6 -4 0	6 4 -2	M24
3 0 -2	0 -2 0	-4 0 -6	4 -2 6	M25
-2 -3 0	0 0 2	-6 4 0	6 -4 2	M26
-3 0 -2	0 2 0	4 0 -6	-4 2 6	M27
2 -3 0	0 0 -2	6 4 0	-6 -4 -2	M28
3 0 2	0 -2 0	4 0 -6	-4 -2 6	M29
2 3 0	0 0 -2	-6 4 0	6 -4 -2	M30
-3 0 2	0 2 0	-4 0 -6	4 2 6	M31
-2 3 0	0 0 2	6 4 0	-6 -4 2	M32
3 0 -2	0 2 0	4 0 6	-4 2 -6	M33
-2 -3 0	0 0 -2	6 -4 0	-6 4 -2	M34
-3 0 -2	0 -2 0	-4 0 6	4 -2 -6	M35
2 -3 0	0 0 2	-6 -4 0	6 4 2	M36
-2 0 3	0 2 0	-6 0 -4	6 2 4	M37
2 0 3	0 -2 0	6 0 -4	-6 -2 4	M38
0 -3 2	2 0 0	0 4 6	2 -4 -6	M39
0 3 -2	2 0 0	0 -4 -6	2 4 6	M40
3 2 0	0 0 -2	-4 6 0	4 -6 -2	M41
-3 2 0	0 0 2	4 6 0	-4 -6 2	M42
-2 0 3	0 -2 0	6 0 4	-6 -2 -4	M43
2 0 3	0 2 0	-6 0 4	6 2 -4	M44
0 -3 2	-2 0 0	0 -4 -6	-2 4 6	M45
0 3 -2	-2 0 0	0 4 6	-2 -4 -6	M46
3 2 0	0 0 2	4 -6 0	-4 6 2	M47
-3 2 0	0 0 -2	-4 -6 0	4 6 -2	M48

Table 28: P - lattice [1 2 3]

[*uvw*]	*hkl A*	*hkl B*	*hkl C*	M No.
1 2 3	1 1 -1	-1 2 -1	2 -1 0	M1
1 2 3	-1 -1 1	1 -2 1	-2 1 0	M2
1 -2 -3	1 -1 1	-1 -2 1	2 1 0	M3
-1 2 -3	-1 1 1	1 2 1	-2 -1 0	M4
-1 -2 3	-1 -1 -1	1 -2 -1	-2 1 0	M5
2 1 -3	1 1 1	2 -1 1	-1 2 0	M6
-2 -1 -3	-1 -1 1	-2 1 1	1 -2 0	M7
3 -2 1	-1 -1 1	-1 -2 -1	0 1 2	M8
-3 -2 -1	1 -1 -1	1 -2 1	0 1 -2	M9
-1 3 2	-1 -1 1	1 -1 2	-2 0 -1	M10
-1 -3 -2	-1 1 -1	1 1 -2	-2 0 1	M11
1 -2 -3	-1 1 -1	1 2 -1	-2 -1 0	M12
-1 2 -3	1 -1 -1	-1 -2 -1	2 1 0	M13
-1 -2 3	1 1 1	-1 2 1	2 -1 0	M14
2 1 -3	-1 -1 -1	-2 1 -1	1 -2 0	M15
-2 -1 -3	1 1 -1	2 -1 -1	-1 2 0	M16
3 -2 1	1 1 -1	1 2 1	0 -1 -2	M17
-3 -2 -1	-1 1 1	-1 2 -1	0 -1 2	M18
-1 3 2	1 1 -1	-1 1 -2	2 0 1	M19
-1 -3 -2	1 -1 1	-1 -1 2	2 0 -1	M20
3 1 2	-1 1 1	-1 -1 2	0 2 -1	M21
2 3 1	1 -1 1	2 -1 -1	-1 0 2	M22
-3 -1 2	1 -1 1	1 1 2	0 -2 -1	M23
-2 3 -1	-1 -1 -1	-2 -1 1	1 0 -2	M24
3 -1 -2	-1 -1 -1	-1 1 -2	0 -2 1	M25
-2 -3 1	-1 1 1	-2 1 -1	1 0 2	M26
-3 1 -2	1 1 -1	1 -1 -2	0 2 1	M27
2 -3 -1	1 1 -1	2 1 1	-1 0 -2	M28
3 1 2	1 -1 -1	1 1 -2	0 -2 1	M29
2 3 1	-1 1 -1	-2 1 1	1 0 -2	M30
-3 -1 2	-1 1 -1	-1 -1 -2	0 2 1	M31
-2 3 -1	1 1 1	2 1 -1	-1 0 2	M32
3 -1 -2	1 1 1	1 -1 2	0 2 -1	M33
-2 -3 1	1 -1 -1	2 -1 1	-1 0 -2	M34
-3 1 -2	-1 -1 1	-1 1 2	0 -2 -1	M35
2 -3 -1	-1 -1 1	-2 -1 -1	1 0 2	M36
-2 1 3	-1 1 -1	-2 -1 -1	1 2 0	M37
2 -1 3	1 -1 -1	2 1 -1	-1 -2 0	M38
1 -3 2	1 1 1	-1 1 2	2 0 -1	M39
1 3 -2	1 -1 -1	-1 -1 -2	2 0 1	M40
3 2 -1	-1 1 -1	-1 2 1	0 -1 -2	M41
-3 2 1	1 1 1	1 2 -1	0 -1 2	M42
-2 1 3	1 -1 1	2 1 1	-1 -2 0	M43
2 -1 3	-1 1 1	-2 -1 1	1 2 0	M44
1 -3 2	-1 -1 -1	1 -1 -2	-2 0 1	M45
1 3 -2	-1 1 1	1 1 2	-2 0 -1	M46
3 2 -1	1 -1 1	1 -2 -1	0 1 2	M47
-3 2 1	-1 -1 -1	-1 -2 1	0 1 -2	M48

Table 29: I - lattice [1 2 3]

[*uvw*]	*hkl A*	*hkl B*	*hkl C*	M No.
1 2 3	1 -2 1	3 0 -1	-2 -2 2	M1
1 2 3	-1 2 -1	-3 0 1	2 2 -2	M2
1 -2 -3	1 2 -1	3 0 1	-2 2 -2	M3
-1 2 -3	-1 -2 -1	-3 0 1	2 -2 -2	M4
-1 -2 3	-1 2 1	-3 0 -1	2 2 2	M5
2 1 -3	-2 1 -1	0 3 1	-2 -2 -2	M6
-2 -1 -3	2 -1 -1	0 -3 1	2 2 -2	M7
3 -2 1	1 2 1	-1 0 3	2 2 -2	M8
-3 -2 -1	-1 2 -1	1 0 -3	-2 2 2	M9
-1 3 2	-1 1 -2	-3 -1 0	2 2 -2	M10
-1 -3 -2	-1 -1 2	-3 1 0	2 -2 2	M11
1 -2 -3	-1 -2 1	-3 0 -1	2 -2 2	M12
-1 2 -3	1 2 1	3 0 -1	-2 2 2	M13
-1 -2 3	1 -2 -1	3 0 1	-2 -2 -2	M14
2 1 -3	2 -1 1	0 -3 -1	2 2 2	M15
-2 -1 -3	-2 1 1	0 3 -1	-2 -2 2	M16
3 -2 1	-1 -2 -1	1 0 -3	-2 -2 2	M17
-3 -2 -1	1 -2 1	-1 0 3	2 -2 -2	M18
-1 3 2	1 -1 2	3 1 0	-2 -2 2	M19
-1 -3 -2	1 1 -2	3 -1 0	-2 2 -2	M20
3 1 2	1 1 -2	-1 3 0	2 -2 -2	M21
2 3 1	-2 1 1	0 -1 3	-2 2 -2	M22
-3 -1 2	-1 -1 -2	1 -3 0	-2 2 -2	M23
-2 3 -1	2 1 -1	0 -1 -3	2 2 2	M24
3 -1 -2	1 -1 2	-1 -3 0	2 2 2	M25
-2 -3 1	2 -1 1	0 1 3	2 -2 -2	M26
-3 1 -2	-1 1 2	1 3 0	-2 -2 2	M27
2 -3 -1	-2 -1 -1	0 1 -3	-2 -2 2	M28
3 1 2	-1 -1 2	1 -3 0	-2 2 2	M29
2 3 1	2 -1 -1	0 1 -3	2 -2 2	M30
-3 -1 2	1 1 2	-1 3 0	2 -2 2	M31
-2 3 -1	-2 -1 1	0 1 3	-2 -2 -2	M32
3 -1 -2	-1 1 -2	1 3 0	-2 -2 -2	M33
-2 -3 1	-2 1 -1	0 -1 -3	-2 2 2	M34
-3 1 -2	1 -1 -2	-1 -3 0	2 2 -2	M35
2 -3 -1	2 1 1	0 -1 3	2 2 -2	M36
-2 1 3	2 1 1	0 3 -1	2 -2 2	M37
2 -1 3	-2 -1 1	0 -3 -1	-2 2 2	M38
1 -3 2	1 -1 -2	3 1 0	-2 -2 -2	M39
1 3 -2	1 1 2	3 -1 0	-2 2 2	M40
3 2 -1	1 -2 -1	-1 0 -3	2 -2 2	M41
-3 2 1	-1 -2 1	1 0 3	-2 -2 -2	M42
-2 1 3	-2 -1 -1	0 -3 1	-2 2 -2	M43
2 -1 3	2 1 -1	0 3 1	2 -2 -2	M44
1 -3 2	-1 1 2	-3 -1 0	2 2 2	M45
1 3 -2	-1 -1 -2	-3 1 0	2 -2 -2	M46
3 2 -1	-1 2 1	1 0 3	-2 2 -2	M47
-3 2 1	1 2 -1	-1 0 -3	2 2 2	M48

Table 30: F - lattice [1 2 3]

[*uvw*]	*hkl A*	*hkl B*	*hkl C*	M No.
1 2 3	1 1 -1	-3 3 -1	4 -2 0	M1
1 2 3	-1 -1 1	3 -3 1	-4 2 0	M2
1 -2 -3	1 -1 1	-3 -3 1	4 2 0	M3
-1 2 -3	-1 1 1	3 3 1	-4 -2 0	M4
-1 -2 3	-1 -1 -1	3 -3 -1	-4 2 0	M5
2 1 -3	1 1 1	3 -3 1	-2 4 0	M6
-2 -1 -3	-1 -1 1	-3 3 1	2 -4 0	M7
3 -2 1	-1 -1 1	-1 -3 -3	0 2 4	M8
-3 -2 -1	1 -1 -1	1 -3 3	0 2 -4	M9
-1 3 2	-1 -1 1	3 -1 3	-4 0 -2	M10
-1 -3 -2	-1 1 -1	3 1 -3	-4 0 2	M11
1 -2 -3	-1 1 -1	3 3 -1	-4 -2 0	M12
-1 2 -3	1 -1 -1	-3 -3 -1	4 2 0	M13
-1 -2 3	1 1 1	-3 3 1	4 -2 0	M14
2 1 -3	-1 -1 -1	-3 3 -1	2 -4 0	M15
-2 -1 -3	1 1 -1	3 -3 -1	-2 4 0	M16
3 -2 1	1 1 -1	1 3 3	0 -2 -4	M17
-3 -2 -1	-1 1 1	-1 3 -3	0 -2 4	M18
-1 3 2	1 1 -1	-3 1 -3	4 0 2	M19
-1 -3 -2	1 -1 1	-3 -1 3	4 0 -2	M20
3 1 2	-1 1 1	-1 -3 3	0 4 -2	M21
2 3 1	1 -1 1	3 -1 -3	-2 0 4	M22
-3 -1 2	1 -1 1	1 3 3	0 -4 -2	M23
-2 3 -1	-1 -1 -1	-3 -1 3	2 0 -4	M24
3 -1 -2	-1 -1 -1	-1 3 -3	0 -4 2	M25
-2 -3 1	-1 1 1	-3 1 -3	2 0 4	M26
-3 1 -2	1 1 -1	1 -3 -3	0 4 2	M27
2 -3 -1	1 1 -1	3 1 3	-2 0 -4	M28
3 1 2	1 -1 -1	1 3 -3	0 -4 2	M29
2 3 1	-1 1 -1	-3 1 3	2 0 -4	M30
-3 -1 2	-1 1 -1	-1 -3 -3	0 4 2	M31
-2 3 -1	1 1 1	3 1 -3	-2 0 4	M32
3 -1 -2	1 1 1	1 -3 3	0 4 -2	M33
-2 -3 1	1 -1 -1	3 -1 3	-2 0 -4	M34
-3 1 -2	-1 -1 1	-1 3 3	0 -4 -2	M35
2 -3 -1	-1 -1 1	-3 -1 -3	2 0 4	M36
-2 1 3	-1 1 -1	-3 -3 -1	2 4 0	M37
2 -1 3	1 -1 -1	3 3 -1	-2 -4 0	M38
1 -3 2	1 1 1	-3 1 3	4 0 -2	M39
1 3 -2	1 -1 -1	-3 -1 -3	4 0 2	M40
3 2 -1	-1 1 -1	-1 3 3	0 -2 -4	M41
-3 2 1	1 1 1	1 3 -3	0 -2 4	M42
-2 1 3	1 -1 1	3 3 1	-2 -4 0	M43
2 -1 3	-1 1 1	-3 -3 1	2 4 0	M44
1 -3 2	-1 -1 -1	3 -1 -3	-4 0 2	M45
1 3 -2	-1 1 1	3 1 3	-4 0 -2	M46
3 2 -1	1 -1 1	1 -3 -3	0 2 4	M47
-3 2 1	-1 -1 -1	-1 -3 3	0 2 -4	M48

Table 31: P - lattice [0 1 4]

[uvw]	hkl A	hkl B	hkl C	M No.
0 1 4	1 0 0	0 4 -1	1 -4 1	M1
0 1 4	-1 0 0	0 -4 1	-1 4 -1	M2
0 -1 -4	1 0 0	0 -4 1	1 4 -1	M3
0 1 -4	-1 0 0	0 4 1	-1 -4 -1	M4
0 -1 4	-1 0 0	0 -4 -1	-1 4 1	M5
1 0 -4	0 1 0	4 0 1	-4 1 -1	M6
-1 0 -4	0 -1 0	-4 0 1	4 -1 -1	M7
4 -1 0	0 0 1	-1 -4 0	1 4 1	M8
-4 -1 0	0 0 -1	1 -4 0	-1 4 -1	M9
0 4 1	-1 0 0	0 -1 4	-1 1 -4	M10
0 -4 -1	-1 0 0	0 1 -4	-1 -1 4	M11
0 -1 -4	-1 0 0	0 4 -1	-1 -4 1	M12
0 1 -4	1 0 0	0 -4 -1	1 4 1	M13
0 -1 4	1 0 0	0 4 1	1 -4 -1	M14
1 0 -4	0 -1 0	-4 0 -1	4 -1 1	M15
-1 0 -4	0 1 0	4 0 -1	-4 1 1	M16
4 -1 0	0 0 -1	1 4 0	-1 -4 -1	M17
-4 -1 0	0 0 1	-1 4 0	1 -4 1	M18
0 4 1	1 0 0	0 1 -4	1 -1 4	M19
0 -4 -1	1 0 0	0 -1 4	1 1 -4	M20
4 0 1	0 1 0	-1 0 4	1 1 -4	M21
1 4 0	0 0 1	4 -1 0	-4 1 1	M22
-4 0 1	0 -1 0	1 0 4	-1 -1 -4	M23
-1 4 0	0 0 -1	-4 -1 0	4 1 -1	M24
4 0 -1	0 -1 0	-1 0 -4	1 -1 4	M25
-1 -4 0	0 0 1	-4 1 0	4 -1 1	M26
-4 0 -1	0 1 0	1 0 -4	-1 1 4	M27
1 -4 0	0 0 -1	4 1 0	-4 -1 -1	M28
4 0 1	0 -1 0	1 0 -4	-1 -1 4	M29
1 4 0	0 0 -1	-4 1 0	4 -1 -1	M30
-4 0 1	0 1 0	-1 0 -4	1 1 4	M31
-1 4 0	0 0 1	4 1 0	-4 -1 1	M32
4 0 -1	0 1 0	1 0 4	-1 1 -4	M33
-1 -4 0	0 0 -1	4 -1 0	-4 1 -1	M34
-4 0 -1	0 -1 0	-1 0 4	1 -1 -4	M35
1 -4 0	0 0 1	-4 -1 0	4 1 1	M36
-1 0 4	0 1 0	-4 0 -1	4 1 1	M37
1 0 4	0 -1 0	4 0 -1	-4 -1 1	M38
0 -4 1	1 0 0	0 1 4	1 -1 -4	M39
0 4 -1	1 0 0	0 -1 -4	1 1 4	M40
4 1 0	0 0 -1	-1 4 0	1 -4 -1	M41
-4 1 0	0 0 1	1 4 0	-1 -4 1	M42
-1 0 4	0 -1 0	4 0 1	-4 -1 -1	M43
1 0 4	0 1 0	-4 0 1	4 1 -1	M44
0 -4 1	-1 0 0	0 -1 -4	-1 1 4	M45
0 4 -1	-1 0 0	0 1 4	-1 -1 -4	M46
4 1 0	0 0 1	1 -4 0	-1 4 1	M47
-4 1 0	0 0 -1	-1 -4 0	1 4 -1	M48

Table 32: I - lattice [0 1 4]

[uvw]	hkl A	hkl B	hkl C	M No.
0 1 4	2 0 0	1 4 -1	1 -4 1	M1
0 1 4	-2 0 0	-1 -4 1	-1 4 -1	M2
0 -1 -4	2 0 0	1 -4 1	1 4 -1	M3
0 1 -4	-2 0 0	-1 4 1	-1 -4 -1	M4
0 -1 4	-2 0 0	-1 -4 -1	-1 4 1	M5
1 0 -4	0 2 0	4 1 1	-4 1 -1	M6
-1 0 -4	0 -2 0	-4 -1 1	4 -1 -1	M7
4 -1 0	0 0 2	-1 -4 1	1 4 1	M8
-4 -1 0	0 0 -2	1 -4 -1	-1 4 -1	M9
0 4 1	-2 0 0	-1 -1 4	-1 1 -4	M10
0 -4 -1	-2 0 0	-1 1 -4	-1 -1 4	M11
0 -1 -4	-2 0 0	-1 4 -1	-1 -4 1	M12
0 1 -4	2 0 0	1 -4 -1	1 4 1	M13
0 -1 4	2 0 0	1 4 1	1 -4 -1	M14
1 0 -4	0 -2 0	-4 -1 -1	4 -1 1	M15
-1 0 -4	0 2 0	4 1 -1	-4 1 1	M16
4 -1 0	0 0 -2	1 4 -1	-1 -4 -1	M17
-4 -1 0	0 0 2	-1 4 1	1 -4 1	M18
0 4 1	2 0 0	1 1 -4	1 -1 4	M19
0 -4 -1	2 0 0	1 -1 4	1 1 -4	M20
4 0 1	0 2 0	-1 1 4	1 1 -4	M21
1 4 0	0 0 2	4 -1 1	-4 1 1	M22
-4 0 1	0 -2 0	1 -1 4	-1 -1 -4	M23
-1 4 0	0 0 -2	-4 -1 -1	4 1 -1	M24
4 0 -1	0 -2 0	-1 -1 -4	1 -1 4	M25
-1 -4 0	0 0 2	-4 1 1	4 -1 1	M26
-4 0 -1	0 2 0	1 1 -4	-1 1 4	M27
1 -4 0	0 0 -2	4 1 -1	-4 -1 -1	M28
4 0 1	0 -2 0	1 -1 -4	-1 -1 4	M29
1 4 0	0 0 -2	-4 1 -1	4 -1 -1	M30
-4 0 1	0 2 0	-1 1 -4	1 1 4	M31
-1 4 0	0 0 2	4 1 1	-4 -1 1	M32
4 0 -1	0 2 0	1 1 4	-1 1 -4	M33
-1 -4 0	0 0 -2	4 -1 -1	-4 1 -1	M34
-4 0 -1	0 -2 0	-1 -1 4	1 -1 -4	M35
1 -4 0	0 0 2	-4 -1 1	4 1 1	M36
-1 0 4	0 2 0	-4 1 -1	4 1 1	M37
1 0 4	0 -2 0	4 -1 -1	-4 -1 1	M38
0 -4 1	2 0 0	1 1 4	1 -1 -4	M39
0 4 -1	2 0 0	1 -1 -4	1 1 4	M40
4 1 0	0 0 -2	-1 4 -1	1 -4 -1	M41
-4 1 0	0 0 2	1 4 1	-1 -4 1	M42
-1 0 4	0 -2 0	4 -1 1	-4 -1 -1	M43
1 0 4	0 2 0	-4 1 1	4 1 -1	M44
0 -4 1	-2 0 0	-1 -1 -4	-1 1 4	M45
0 4 -1	-2 0 0	-1 1 4	-1 -1 -4	M46
4 1 0	0 0 2	1 -4 1	-1 4 1	M47
-4 1 0	0 0 -2	-1 -4 -1	1 4 -1	M48

Table 33: F - lattice [0 1 4]

[*uvw*]	*hkl A*	*hkl B*	*hkl C*	M No.
0 1 4	2 0 0	0 8 -2	2 -8 2	M1
0 1 4	-2 0 0	0 -8 2	-2 8 -2	M2
0 -1 -4	2 0 0	0 -8 2	2 8 -2	M3
0 1 -4	-2 0 0	0 8 2	-2 -8 -2	M4
0 -1 4	-2 0 0	0 -8 -2	-2 8 2	M5
1 0 -4	0 2 0	8 0 2	-8 2 -2	M6
-1 0 -4	0 -2 0	-8 0 2	8 -2 -2	M7
4 -1 0	0 0 2	-2 -8 0	2 8 2	M8
-4 -1 0	0 0 -2	2 -8 0	-2 8 -2	M9
0 4 1	-2 0 0	0 -2 8	-2 2 -8	M10
0 -4 -1	-2 0 0	0 2 -8	-2 -2 8	M11
0 -1 -4	-2 0 0	0 8 -2	-2 -8 2	M12
0 1 -4	2 0 0	0 -8 -2	2 8 2	M13
0 -1 4	2 0 0	0 8 2	2 -8 -2	M14
1 0 -4	0 -2 0	-8 0 -2	8 -2 2	M15
-1 0 -4	0 2 0	8 0 -2	-8 2 2	M16
4 -1 0	0 0 -2	2 8 0	-2 -8 -2	M17
-4 -1 0	0 0 2	-2 8 0	2 -8 2	M18
0 4 1	2 0 0	0 2 -8	2 -2 8	M19
0 -4 -1	2 0 0	0 -2 8	2 2 -8	M20
4 0 1	0 2 0	-2 0 8	2 2 -8	M21
1 4 0	0 0 2	8 -2 0	-8 2 2	M22
-4 0 1	0 -2 0	2 0 8	-2 -2 -8	M23
-1 4 0	0 0 -2	-8 -2 0	8 2 -2	M24
4 0 -1	0 -2 0	-2 0 -8	2 -2 8	M25
-1 -4 0	0 0 2	-8 2 0	8 -2 2	M26
-4 0 -1	0 2 0	2 0 -8	-2 2 8	M27
1 -4 0	0 0 -2	8 2 0	-8 -2 -2	M28
4 0 1	0 -2 0	2 0 -8	-2 -2 8	M29
1 4 0	0 0 -2	-8 2 0	8 -2 -2	M30
-4 0 1	0 2 0	-2 0 -8	2 2 8	M31
-1 4 0	0 0 2	8 2 0	-8 -2 2	M32
4 0 -1	0 2 0	2 0 8	-2 2 -8	M33
-1 -4 0	0 0 -2	8 -2 0	-8 2 -2	M34
-4 0 -1	0 -2 0	-2 0 8	2 -2 -8	M35
1 -4 0	0 0 2	-8 -2 0	8 2 2	M36
-1 0 4	0 2 0	-8 0 -2	8 2 2	M37
1 0 4	0 -2 0	8 0 -2	-8 -2 2	M38
0 -4 1	2 0 0	0 2 8	2 -2 -8	M39
0 4 -1	2 0 0	0 -2 -8	2 2 8	M40
4 1 0	0 0 -2	-2 8 0	2 -8 -2	M41
-4 1 0	0 0 2	2 8 0	-2 -8 2	M42
-1 0 4	0 -2 0	8 0 2	-8 -2 -2	M43
1 0 4	0 2 0	-8 0 2	8 2 -2	M44
0 -4 1	-2 0 0	0 -2 -8	-2 2 8	M45
0 4 -1	-2 0 0	0 2 8	-2 -2 -8	M46
4 1 0	0 0 2	2 -8 0	-2 8 2	M47
-4 1 0	0 0 -2	-2 -8 0	2 8 -2	M48

Table 34: P - lattice [2 2 3]

[uvw]	hkl A	hkl B	hkl C	M No.
2 2 3	1 -1 0	2 1 -2	-1 -2 2	M1
2 2 3	-1 1 0	-2 -1 2	1 2 -2	M2
2 -2 -3	1 1 0	2 -1 2	-1 2 -2	M3
-2 2 -3	-1 -1 0	-2 1 2	1 -2 -2	M4
-2 -2 3	-1 1 0	-2 -1 -2	1 2 2	M5
2 2 -3	-1 1 0	1 2 2	-2 -1 -2	M6
-2 -2 -3	1 -1 0	-1 -2 2	2 1 -2	M7
3 -2 2	0 1 1	-2 -1 2	2 2 -1	M8
-3 -2 -2	0 1 -1	2 -1 -2	-2 2 1	M9
-2 3 2	-1 0 -1	-2 -2 1	1 2 -2	M10
-2 -3 -2	-1 0 1	-2 2 -1	1 -2 2	M11
2 -2 -3	-1 -1 0	-2 1 -2	1 -2 2	M12
-2 2 -3	1 1 0	2 -1 -2	-1 2 2	M13
-2 -2 3	1 -1 0	2 1 2	-1 -2 -2	M14
2 2 -3	1 -1 0	-1 -2 -2	2 1 2	M15
-2 -2 -3	-1 1 0	1 2 -2	-2 -1 2	M16
3 -2 2	0 -1 -1	2 1 -2	-2 -2 1	M17
-3 -2 -2	0 -1 1	-2 1 2	2 -2 -1	M18
-2 3 2	1 0 1	2 2 -1	-1 -2 2	M19
-2 -3 -2	1 0 -1	2 -2 1	-1 2 -2	M20
3 2 2	0 1 -1	-2 2 1	2 -1 -2	M21
2 3 2	-1 0 1	1 -2 2	-2 2 -1	M22
-3 -2 2	0 -1 -1	2 -2 1	-2 1 -2	M23
-2 3 -2	1 0 -1	-1 -2 -2	2 2 1	M24
3 -2 -2	0 -1 1	-2 -2 -1	2 1 2	M25
-2 -3 2	1 0 1	-1 2 2	2 -2 -1	M26
-3 2 -2	0 1 1	2 2 -1	-2 -1 2	M27
2 -3 -2	-1 0 -1	1 2 -2	-2 -2 1	M28
3 2 2	0 -1 1	2 -2 -1	-2 1 2	M29
2 3 2	1 0 -1	-1 2 -2	2 -2 1	M30
-3 -2 2	0 1 1	-2 2 -1	2 -1 2	M31
-2 3 -2	-1 0 1	1 2 2	-2 -2 -1	M32
3 -2 -2	0 1 -1	2 2 1	-2 -1 -2	M33
-2 -3 2	-1 0 -1	1 -2 -2	-2 2 1	M34
-3 2 -2	0 -1 -1	-2 -2 1	2 1 -2	M35
2 -3 -2	1 0 1	-1 -2 2	2 2 -1	M36
-2 2 3	1 1 0	-1 2 -2	2 -1 2	M37
2 -2 3	-1 -1 0	1 -2 -2	-2 1 2	M38
2 -3 2	1 0 -1	2 2 1	-1 -2 -2	M39
2 3 -2	1 0 1	2 -2 -1	-1 2 2	M40
3 2 -2	0 -1 -1	-2 1 -2	2 -2 1	M41
-3 2 2	0 -1 1	2 1 2	-2 -2 -1	M42
-2 2 3	-1 -1 0	1 -2 2	-2 1 -2	M43
2 -2 3	1 1 0	-1 2 2	2 -1 -2	M44
2 -3 2	-1 0 1	-2 -2 -1	1 2 2	M45
2 3 -2	-1 0 -1	-2 2 1	1 -2 -2	M46
3 2 -2	0 1 1	2 -1 2	-2 2 -1	M47
-3 2 2	0 1 -1	-2 -1 -2	2 2 1	M48

Table 35: I - lattice [2 2 3]

[*uvw*]	*hkl A*	*hkl B*	*hkl C*	M No.
2 2 3	1 -1 0	3 3 -4	-2 -4 4	M1
2 2 3	-1 1 0	-3 -3 4	2 4 -4	M2
2 -2 -3	1 1 0	3 -3 4	-2 4 -4	M3
-2 2 -3	-1 -1 0	-3 3 4	2 -4 -4	M4
-2 -2 3	-1 1 0	-3 -3 -4	2 4 4	M5
2 2 -3	-1 1 0	3 3 4	-4 -2 -4	M6
-2 -2 -3	1 -1 0	-3 -3 4	4 2 -4	M7
3 -2 2	0 1 1	-4 -3 3	4 4 -2	M8
-3 -2 -2	0 1 -1	4 -3 -3	-4 4 2	M9
-2 3 2	-1 0 -1	-3 -4 3	2 4 -4	M10
-2 -3 -2	-1 0 1	-3 4 -3	2 -4 4	M11
2 -2 -3	-1 -1 0	-3 3 -4	2 -4 4	M12
-2 2 -3	1 1 0	3 -3 -4	-2 4 4	M13
-2 -2 3	1 -1 0	3 3 4	-2 -4 -4	M14
2 2 -3	1 -1 0	-3 -3 -4	4 2 4	M15
-2 -2 -3	-1 1 0	3 3 -4	-4 -2 4	M16
3 -2 2	0 -1 -1	4 3 -3	-4 -4 2	M17
-3 -2 -2	0 -1 1	-4 3 3	4 -4 -2	M18
-2 3 2	1 0 1	3 4 -3	-2 -4 4	M19
-2 -3 -2	1 0 -1	3 -4 3	-2 4 -4	M20
3 2 2	0 1 -1	-4 3 3	4 -2 -4	M21
2 3 2	-1 0 1	3 -4 3	-4 4 -2	M22
-3 -2 2	0 -1 -1	4 -3 3	-4 2 -4	M23
-2 3 -2	1 0 -1	-3 -4 -3	4 4 2	M24
3 -2 -2	0 -1 1	-4 -3 -3	4 2 4	M25
-2 -3 2	1 0 1	-3 4 3	4 -4 -2	M26
-3 2 -2	0 1 1	4 3 -3	-4 -2 4	M27
2 -3 -2	-1 0 -1	3 4 -3	-4 -4 2	M28
3 2 2	0 -1 1	4 -3 -3	-4 2 4	M29
2 3 2	1 0 -1	-3 4 -3	4 -4 2	M30
-3 -2 2	0 1 1	-4 3 -3	4 -2 4	M31
-2 3 -2	-1 0 1	3 4 3	-4 -4 -2	M32
3 -2 -2	0 1 -1	4 3 3	-4 -2 -4	M33
-2 -3 2	-1 0 -1	3 -4 -3	-4 4 2	M34
-3 2 -2	0 -1 -1	-4 -3 3	4 2 -4	M35
2 -3 -2	1 0 1	-3 -4 3	4 4 -2	M36
-2 2 3	1 1 0	-3 3 -4	4 -2 4	M37
2 -2 3	-1 -1 0	3 -3 -4	-4 2 4	M38
2 -3 2	1 0 -1	3 4 3	-2 -4 -4	M39
2 3 -2	1 0 1	3 -4 -3	-2 4 4	M40
3 2 -2	0 -1 -1	-4 3 -3	4 -4 2	M41
-3 2 2	0 -1 1	4 3 3	-4 -4 -2	M42
-2 2 3	-1 -1 0	3 -3 4	-4 2 -4	M43
2 -2 3	1 1 0	-3 3 4	4 -2 -4	M44
2 -3 2	-1 0 1	-3 -4 -3	2 4 4	M45
2 3 -2	-1 0 -1	-3 4 3	2 -4 -4	M46
3 2 -2	0 1 1	4 -3 3	-4 4 -2	M47
-3 2 2	0 1 -1	-4 -3 -3	4 4 2	M48

Table 36: F - lattice [2 2 3]

[*uvw*]	*hkl A*	*hkl B*	*hkl C*	M No.
2 2 3	-2 2 0	-4 -2 4	2 4 -4	M1
2 2 3	2 -2 0	4 2 -4	-2 -4 4	M2
2 -2 -3	-2 -2 0	-4 2 -4	2 -4 4	M3
-2 2 -3	2 2 0	4 -2 -4	-2 4 4	M4
-2 -2 3	2 -2 0	4 2 4	-2 -4 -4	M5
2 2 -3	2 -2 0	-2 -4 -4	4 2 4	M6
-2 -2 -3	-2 2 0	2 4 -4	-4 -2 4	M7
3 -2 2	0 -2 -2	4 2 -4	-4 -4 2	M8
-3 -2 -2	0 -2 2	-4 2 4	4 -4 -2	M9
-2 3 2	2 0 2	4 4 -2	-2 -4 4	M10
-2 -3 -2	2 0 -2	4 -4 2	-2 4 -4	M11
2 -2 -3	2 2 0	4 -2 4	-2 4 -4	M12
-2 2 -3	-2 -2 0	-4 2 4	2 -4 -4	M13
-2 -2 3	-2 2 0	-4 -2 -4	2 4 4	M14
2 2 -3	-2 2 0	2 4 4	-4 -2 -4	M15
-2 -2 -3	2 -2 0	-2 -4 4	4 2 -4	M16
3 -2 2	0 2 2	-4 -2 4	4 4 -2	M17
-3 -2 -2	0 2 -2	4 -2 -4	-4 4 2	M18
-2 3 2	-2 0 -2	-4 -4 2	2 4 -4	M19
-2 -3 -2	-2 0 2	-4 4 -2	2 -4 4	M20
3 2 2	0 -2 2	4 -4 -2	-4 2 4	M21
2 3 2	2 0 -2	-2 4 -4	4 -4 2	M22
-3 -2 2	0 2 2	-4 4 -2	4 -2 4	M23
-2 3 -2	-2 0 2	2 4 4	-4 -4 -2	M24
3 -2 -2	0 2 -2	4 4 2	-4 -2 -4	M25
-2 -3 2	-2 0 -2	2 -4 -4	-4 4 2	M26
-3 2 -2	0 -2 -2	-4 -4 2	4 2 -4	M27
2 -3 -2	2 0 2	-2 -4 4	4 4 -2	M28
3 2 2	0 2 -2	-4 4 2	4 -2 -4	M29
2 3 2	-2 0 2	2 -4 4	-4 4 -2	M30
-3 -2 2	0 -2 -2	4 -4 2	-4 2 -4	M31
-2 3 -2	2 0 -2	-2 -4 -4	4 4 2	M32
3 -2 -2	0 -2 2	-4 -4 -2	4 2 4	M33
-2 -3 2	2 0 2	-2 4 4	4 -4 -2	M34
-3 2 -2	0 2 2	4 4 -2	-4 -2 4	M35
2 -3 -2	-2 0 -2	2 4 -4	-4 -4 2	M36
-2 2 3	-2 -2 0	2 -4 4	-4 2 -4	M37
2 -2 3	2 2 0	-2 4 4	4 -2 -4	M38
2 -3 2	-2 0 2	-4 -4 -2	2 4 4	M39
2 3 -2	-2 0 -2	-4 4 2	2 -4 -4	M40
3 2 -2	0 2 2	4 -2 4	-4 4 -2	M41
-3 2 2	0 2 -2	-4 -2 -4	4 4 2	M42
-2 2 3	2 2 0	-2 4 -4	4 -2 4	M43
2 -2 3	-2 -2 0	2 -4 -4	-4 2 4	M44
2 -3 2	2 0 -2	4 4 2	-2 -4 -4	M45
2 3 -2	2 0 2	4 -4 -2	-2 4 4	M46
3 2 -2	0 -2 -2	-4 2 -4	4 -4 2	M47
-3 2 2	0 -2 2	4 2 4	-4 -4 -2	M48

Table 37: P - lattice [1 1 4]

[*uvw*]	*hkl A*	*hkl B*	*hkl C*	M No.
1 1 4	1 -1 0	2 2 -1	-1 -3 1	M1
1 1 4	-1 1 0	-2 -2 1	1 3 -1	M2
1 -1 -4	1 1 0	2 -2 1	-1 3 -1	M3
-1 1 -4	-1 -1 0	-2 2 1	1 -3 -1	M4
-1 -1 4	-1 1 0	-2 -2 -1	1 3 1	M5
1 1 -4	-1 1 0	2 2 1	-3 -1 -1	M6
-1 -1 -4	1 -1 0	-2 -2 1	3 1 -1	M7
4 -1 1	0 1 1	-1 -2 2	1 3 -1	M8
-4 -1 -1	0 1 -1	1 -2 -2	-1 3 1	M9
-1 4 1	-1 0 -1	-2 -1 2	1 1 -3	M10
-1 -4 -1	-1 0 1	-2 1 -2	1 -1 3	M11
1 -1 -4	-1 -1 0	-2 2 -1	1 -3 1	M12
-1 1 -4	1 1 0	2 -2 -1	-1 3 1	M13
-1 -1 4	1 -1 0	2 2 1	-1 -3 -1	M14
1 1 -4	1 -1 0	-2 -2 -1	3 1 1	M15
-1 -1 -4	-1 1 0	2 2 -1	-3 -1 1	M16
4 -1 1	0 -1 -1	1 2 -2	-1 -3 1	M17
-4 -1 -1	0 -1 1	-1 2 2	1 -3 -1	M18
-1 4 1	1 0 1	2 1 -2	-1 -1 3	M19
-1 -4 -1	1 0 -1	2 -1 2	-1 1 -3	M20
4 1 1	0 1 -1	-1 2 2	1 -1 -3	M21
1 4 1	-1 0 1	2 -1 2	-3 1 -1	M22
-4 -1 1	0 -1 -1	1 -2 2	-1 1 -3	M23
-1 4 -1	1 0 -1	-2 -1 -2	3 1 1	M24
4 -1 -1	0 -1 1	-1 -2 -2	1 1 3	M25
-1 -4 1	1 0 1	-2 1 2	3 -1 -1	M26
-4 1 -1	0 1 1	1 2 -2	-1 -1 3	M27
1 -4 -1	-1 0 -1	2 1 -2	-3 -1 1	M28
4 1 1	0 -1 1	1 -2 -2	-1 1 3	M29
1 4 1	1 0 -1	-2 1 -2	3 -1 1	M30
-4 -1 1	0 1 1	-1 2 -2	1 -1 3	M31
-1 4 -1	-1 0 1	2 1 2	-3 -1 -1	M32
4 -1 -1	0 1 -1	1 2 2	-1 -1 -3	M33
-1 -4 1	-1 0 -1	2 -1 -2	-3 1 1	M34
-4 1 -1	0 -1 -1	-1 -2 2	1 1 -3	M35
1 -4 -1	1 0 1	-2 -1 2	3 1 -1	M36
-1 1 4	1 1 0	-2 2 -1	3 -1 1	M37
1 -1 4	-1 -1 0	2 -2 -1	-3 1 1	M38
1 -4 1	1 0 -1	2 1 2	-1 -1 -3	M39
1 4 -1	1 0 1	2 -1 -2	-1 1 3	M40
4 1 -1	0 -1 -1	-1 2 -2	1 -3 1	M41
-4 1 1	0 -1 1	1 2 2	-1 -3 -1	M42
-1 1 4	-1 -1 0	2 -2 1	-3 1 -1	M43
1 -1 4	1 1 0	-2 2 1	3 -1 -1	M44
1 -4 1	-1 0 1	-2 -1 -2	1 1 3	M45
1 4 -1	-1 0 -1	-2 1 2	1 -1 -3	M46
4 1 -1	0 1 1	1 -2 2	-1 3 -1	M47
-4 1 1	0 1 -1	-1 -2 -2	1 3 1	M48

Table 38: I - lattice [1 1 4]

[*uvw*]	*hkl A*	*hkl B*	*hkl C*	M No.
1 1 4	1 -1 0	4 4 -2	-3 -5 2	M1
1 1 4	-1 1 0	-4 -4 2	3 5 -2	M2
1 -1 -4	1 1 0	4 -4 2	-3 5 -2	M3
-1 1 -4	-1 -1 0	-4 4 2	3 -5 -2	M4
-1 -1 4	-1 1 0	-4 -4 -2	3 5 2	M5
1 1 -4	-1 1 0	4 4 2	-5 -3 -2	M6
-1 -1 -4	1 -1 0	-4 -4 2	5 3 -2	M7
4 -1 1	0 1 1	-2 -4 4	2 5 -3	M8
-4 -1 -1	0 1 -1	2 -4 -4	-2 5 3	M9
-1 4 1	-1 0 -1	-4 -2 4	3 2 -5	M10
-1 -4 -1	-1 0 1	-4 2 -4	3 -2 5	M11
1 -1 -4	-1 -1 0	-4 4 -2	3 -5 2	M12
-1 1 -4	1 1 0	4 -4 -2	-3 5 2	M13
-1 -1 4	1 -1 0	4 4 2	-3 -5 -2	M14
1 1 -4	1 -1 0	-4 -4 -2	5 3 2	M15
-1 -1 -4	-1 1 0	4 4 -2	-5 -3 2	M16
4 -1 1	0 -1 -1	2 4 -4	-2 -5 3	M17
-4 -1 -1	0 -1 1	-2 4 4	2 -5 -3	M18
-1 4 1	1 0 1	4 2 -4	-3 -2 5	M19
-1 -4 -1	1 0 -1	4 -2 4	-3 2 -5	M20
4 1 1	0 1 -1	-2 4 4	2 -3 -5	M21
1 4 1	-1 0 1	4 -2 4	-5 2 -3	M22
-4 -1 1	0 -1 -1	2 -4 4	-2 3 -5	M23
-1 4 -1	1 0 -1	-4 -2 -4	5 2 3	M24
4 -1 -1	0 -1 1	-2 -4 -4	2 3 5	M25
-1 -4 1	1 0 1	-4 2 4	5 -2 -3	M26
-4 1 -1	0 1 1	2 4 -4	-2 -3 5	M27
1 -4 -1	-1 0 -1	4 2 -4	-5 -2 3	M28
4 1 1	0 -1 1	2 -4 -4	-2 3 5	M29
1 4 1	1 0 -1	-4 2 -4	5 -2 3	M30
-4 -1 1	0 1 1	-2 4 -4	2 -3 5	M31
-1 4 -1	-1 0 1	4 2 4	-5 -2 -3	M32
4 -1 -1	0 1 -1	2 4 4	-2 -3 -5	M33
-1 -4 1	-1 0 -1	4 -2 -4	-5 2 3	M34
-4 1 -1	0 -1 -1	-2 -4 4	2 3 -5	M35
1 -4 -1	1 0 1	-4 -2 4	5 2 -3	M36
-1 1 4	1 1 0	-4 4 -2	5 -3 2	M37
1 -1 4	-1 -1 0	4 -4 -2	-5 3 2	M38
1 -4 1	1 0 -1	4 2 4	-3 -2 -5	M39
1 4 -1	1 0 1	4 -2 -4	-3 2 5	M40
4 1 -1	0 -1 -1	-2 4 -4	2 -5 3	M41
-4 1 1	0 -1 1	2 4 4	-2 -5 -3	M42
-1 1 4	-1 -1 0	4 -4 2	-5 3 -2	M43
1 -1 4	1 1 0	-4 4 2	5 -3 -2	M44
1 -4 1	-1 0 1	-4 -2 -4	3 2 5	M45
1 4 -1	-1 0 -1	-4 2 4	3 -2 -5	M46
4 1 -1	0 1 1	2 -4 4	-2 5 -3	M47
-4 1 1	0 1 -1	-2 -4 -4	2 5 3	M48

Table 39: F - lattice [1 1 4]

[*uvw*]	*hkl A*	*hkl B*	*hkl C*	M No.
1 1 4	-2 2 0	-3 -1 1	1 3 -1	M1
1 1 4	2 -2 0	3 1 -1	-1 -3 1	M2
1 -1 -4	-2 -2 0	-3 1 -1	1 -3 1	M3
-1 1 -4	2 2 0	3 -1 -1	-1 3 1	M4
-1 -1 4	2 -2 0	3 1 1	-1 -3 -1	M5
1 1 -4	2 -2 0	-1 -3 -1	3 1 1	M6
-1 -1 -4	-2 2 0	1 3 -1	-3 -1 1	M7
4 -1 1	0 -2 -2	1 1 -3	-1 -3 1	M8
-4 -1 -1	0 -2 2	-1 1 3	1 -3 -1	M9
-1 4 1	2 0 2	3 1 -1	-1 -1 3	M10
-1 -4 -1	2 0 -2	3 -1 1	-1 1 -3	M11
1 -1 -4	2 2 0	3 -1 1	-1 3 -1	M12
-1 1 -4	-2 -2 0	-3 1 1	1 -3 -1	M13
-1 -1 4	-2 2 0	-3 -1 -1	1 3 1	M14
1 1 -4	-2 2 0	1 3 1	-3 -1 -1	M15
-1 -1 -4	2 -2 0	-1 -3 1	3 1 -1	M16
4 -1 1	0 2 2	-1 -1 3	1 3 -1	M17
-4 -1 -1	0 2 -2	1 -1 -3	-1 3 1	M18
-1 4 1	-2 0 -2	-3 -1 1	1 1 -3	M19
-1 -4 -1	-2 0 2	-3 1 -1	1 -1 3	M20
4 1 1	0 -2 2	1 -3 -1	-1 1 3	M21
1 4 1	2 0 -2	-1 1 -3	3 -1 1	M22
-4 -1 1	0 2 2	-1 3 -1	1 -1 3	M23
-1 4 -1	-2 0 2	1 1 3	-3 -1 -1	M24
4 -1 -1	0 2 -2	1 3 1	-1 -1 -3	M25
-1 -4 1	-2 0 -2	1 -1 -3	-3 1 1	M26
-4 1 -1	0 -2 -2	-1 -3 1	1 1 -3	M27
1 -4 -1	2 0 2	-1 -1 3	3 1 -1	M28
4 1 1	0 2 -2	-1 3 1	1 -1 -3	M29
1 4 1	-2 0 2	1 -1 3	-3 1 -1	M30
-4 -1 1	0 -2 -2	1 -3 1	-1 1 -3	M31
-1 4 -1	2 0 -2	-1 -1 -3	3 1 1	M32
4 -1 -1	0 -2 2	-1 -3 -1	1 1 3	M33
-1 -4 1	2 0 2	-1 1 3	3 -1 -1	M34
-4 1 -1	0 2 2	1 3 -1	-1 -1 3	M35
1 -4 -1	-2 0 -2	1 1 -3	-3 -1 1	M36
-1 1 4	-2 -2 0	1 -3 1	-3 1 -1	M37
1 -1 4	2 2 0	-1 3 1	3 -1 -1	M38
1 -4 1	-2 0 2	-3 -1 -1	1 1 3	M39
1 4 -1	-2 0 -2	-3 1 1	1 -1 -3	M40
4 1 -1	0 2 2	1 -1 3	-1 3 -1	M41
-4 1 1	0 2 -2	-1 -1 -3	1 3 1	M42
-1 1 4	2 2 0	-1 3 -1	3 -1 1	M43
1 -1 4	-2 -2 0	1 -3 -1	-3 1 1	M44
1 -4 1	2 0 -2	3 1 1	-1 -1 -3	M45
1 4 -1	2 0 2	3 -1 -1	-1 1 3	M46
4 1 -1	0 -2 -2	-1 1 -3	1 -3 1	M47
-4 1 1	0 -2 2	1 1 3	-1 -3 -1	M48

Table 40: P - lattice [1 3 3]

[*uvw*]	*hkl A*	*hkl B*	*hkl C*	M No.
1 3 3	0 1 -1	-3 1 0	3 0 -1	M1
1 3 3	0 -1 1	3 -1 0	-3 0 1	M2
1 -3 -3	0 -1 1	-3 -1 0	3 0 1	M3
-1 3 -3	0 1 1	3 1 0	-3 0 1	M4
-1 -3 3	0 -1 -1	3 -1 0	-3 0 -1	M5
3 1 -3	1 0 1	1 -3 0	0 3 1	M6
-3 -1 -3	-1 0 1	-1 3 0	0 -3 1	M7
3 -3 1	-1 -1 0	0 -1 -3	-1 0 3	M8
-3 -3 -1	1 -1 0	0 -1 3	1 0 -3	M9
-1 3 3	0 -1 1	3 0 1	-3 -1 0	M10
-1 -3 -3	0 1 -1	3 0 -1	-3 1 0	M11
1 -3 -3	0 1 -1	3 1 0	-3 0 -1	M12
-1 3 -3	0 -1 -1	-3 -1 0	3 0 -1	M13
-1 -3 3	0 1 1	-3 1 0	3 0 1	M14
3 1 -3	-1 0 -1	-1 3 0	0 -3 -1	M15
-3 -1 -3	1 0 -1	1 -3 0	0 3 -1	M16
3 -3 1	1 1 0	0 1 3	1 0 -3	M17
-3 -3 -1	-1 1 0	0 1 -3	-1 0 3	M18
-1 3 3	0 1 -1	-3 0 -1	3 1 0	M19
-1 -3 -3	0 -1 1	-3 0 1	3 -1 0	M20
3 1 3	-1 0 1	0 -3 1	-1 3 0	M21
3 3 1	1 -1 0	1 0 -3	0 -1 3	M22
-3 -1 3	1 0 1	0 3 1	1 -3 0	M23
-3 3 -1	-1 -1 0	-1 0 3	0 -1 -3	M24
3 -1 -3	-1 0 -1	0 3 -1	-1 -3 0	M25
-3 -3 1	-1 1 0	-1 0 -3	0 1 3	M26
-3 1 -3	1 0 -1	0 -3 -1	1 3 0	M27
3 -3 -1	1 1 0	1 0 3	0 1 -3	M28
3 1 3	1 0 -1	0 3 -1	1 -3 0	M29
3 3 1	-1 1 0	-1 0 3	0 1 -3	M30
-3 -1 3	-1 0 -1	0 -3 -1	-1 3 0	M31
-3 3 -1	1 1 0	1 0 -3	0 1 3	M32
3 -1 -3	1 0 1	0 -3 1	1 3 0	M33
-3 -3 1	1 -1 0	1 0 3	0 -1 -3	M34
-3 1 -3	-1 0 1	0 3 1	-1 -3 0	M35
3 -3 -1	-1 -1 0	-1 0 -3	0 -1 3	M36
-3 1 3	-1 0 -1	-1 -3 0	0 3 -1	M37
3 -1 3	1 0 -1	1 3 0	0 -3 -1	M38
1 -3 3	0 1 1	-3 0 1	3 1 0	M39
1 3 -3	0 -1 -1	-3 0 -1	3 -1 0	M40
3 3 -1	-1 1 0	0 1 3	-1 0 -3	M41
-3 3 1	1 1 0	0 1 -3	1 0 3	M42
-3 1 3	1 0 1	1 3 0	0 -3 1	M43
3 -1 3	-1 0 1	-1 -3 0	0 3 1	M44
1 -3 3	0 -1 -1	3 0 -1	-3 -1 0	M45
1 3 -3	0 1 1	3 0 1	-3 1 0	M46
3 3 -1	1 -1 0	0 -1 -3	1 0 3	M47
-3 3 1	-1 -1 0	0 -1 3	-1 0 -3	M48

Table 41: I - lattice [1 3 3]

[uvw]	hkl A	hkl B	hkl C	M No.
1 3 3	0 1 -1	-3 1 0	3 0 -1	M1
1 3 3	0 -1 1	3 -1 0	-3 0 1	M2
1 -3 -3	0 -1 1	-3 -1 0	3 0 1	M3
-1 3 -3	0 1 1	3 1 0	-3 0 1	M4
-1 -3 3	0 -1 -1	3 -1 0	-3 0 -1	M5
3 1 -3	1 0 1	1 -3 0	0 3 1	M6
-3 -1 -3	-1 0 1	-1 3 0	0 -3 1	M7
3 -3 1	-1 -1 0	0 -1 -3	-1 0 3	M8
-3 -3 -1	1 -1 0	0 -1 3	1 0 -3	M9
-1 3 3	0 -1 1	3 0 1	-3 -1 0	M10
-1 -3 -3	0 1 -1	3 0 -1	-3 1 0	M11
1 -3 -3	0 1 -1	3 1 0	-3 0 -1	M12
-1 3 -3	0 -1 -1	-3 -1 0	3 0 -1	M13
-1 -3 3	0 1 1	-3 1 0	3 0 1	M14
3 1 -3	-1 0 -1	-1 3 0	0 -3 -1	M15
-3 -1 -3	1 0 -1	1 -3 0	0 3 -1	M16
3 -3 1	1 1 0	0 1 3	1 0 -3	M17
-3 -3 -1	-1 1 0	0 1 -3	-1 0 3	M18
-1 3 3	0 1 -1	-3 0 -1	3 1 0	M19
-1 -3 -3	0 -1 1	-3 0 1	3 -1 0	M20
3 1 3	-1 0 1	0 -3 1	-1 3 0	M21
3 3 1	1 -1 0	1 0 -3	0 -1 3	M22
-3 -1 3	1 0 1	0 3 1	1 -3 0	M23
-3 3 -1	-1 -1 0	-1 0 3	0 -1 -3	M24
3 -1 -3	-1 0 -1	0 3 -1	-1 -3 0	M25
-3 -3 1	-1 1 0	-1 0 -3	0 1 3	M26
-3 1 -3	1 0 -1	0 -3 -1	1 3 0	M27
3 -3 -1	1 1 0	1 0 3	0 1 -3	M28
3 1 3	1 0 -1	0 3 -1	1 -3 0	M29
3 3 1	-1 1 0	-1 0 3	0 1 -3	M30
-3 -1 3	-1 0 -1	0 -3 -1	-1 3 0	M31
-3 3 -1	1 1 0	1 0 -3	0 1 3	M32
3 -1 -3	1 0 1	0 -3 1	1 3 0	M33
-3 -3 1	1 -1 0	1 0 3	0 -1 -3	M34
-3 1 -3	-1 0 1	0 3 1	-1 -3 0	M35
3 -3 -1	-1 -1 0	-1 0 -3	0 -1 3	M36
-3 1 3	-1 0 -1	-1 -3 0	0 3 -1	M37
3 -1 3	1 0 -1	1 3 0	0 -3 -1	M38
1 -3 3	0 1 1	-3 0 1	3 1 0	M39
1 3 -3	0 -1 -1	-3 0 -1	3 -1 0	M40
3 3 -1	-1 1 0	0 1 3	-1 0 -3	M41
-3 3 1	1 1 0	0 1 -3	1 0 3	M42
-3 1 3	1 0 1	1 3 0	0 -3 1	M43
3 -1 3	-1 0 1	-1 -3 0	0 3 1	M44
1 -3 3	0 -1 -1	3 0 -1	-3 -1 0	M45
1 3 -3	0 1 1	3 0 1	-3 1 0	M46
3 3 -1	1 -1 0	0 -1 -3	1 0 3	M47
-3 3 1	-1 -1 0	0 -1 3	-1 0 -3	M48

Table 42: F - lattice [1 3 3]

[*uvw*]	*hkl A*	*hkl B*	*hkl C*	M No.
1 3 3	0 2 -2	-6 2 0	6 0 -2	M1
1 3 3	0 -2 2	6 -2 0	-6 0 2	M2
1 -3 -3	0 -2 2	-6 -2 0	6 0 2	M3
-1 3 -3	0 2 2	6 2 0	-6 0 2	M4
-1 -3 3	0 -2 -2	6 -2 0	-6 0 -2	M5
3 1 -3	2 0 2	2 -6 0	0 6 2	M6
-3 -1 -3	-2 0 2	-2 6 0	0 -6 2	M7
3 -3 1	-2 -2 0	0 -2 -6	-2 0 6	M8
-3 -3 -1	2 -2 0	0 -2 6	2 0 -6	M9
-1 3 3	0 -2 2	6 0 2	-6 -2 0	M10
-1 -3 -3	0 2 -2	6 0 -2	-6 2 0	M11
1 -3 -3	0 2 -2	6 2 0	-6 0 -2	M12
-1 3 -3	0 -2 -2	-6 -2 0	6 0 -2	M13
-1 -3 3	0 2 2	-6 2 0	6 0 2	M14
3 1 -3	-2 0 -2	-2 6 0	0 -6 -2	M15
-3 -1 -3	2 0 -2	2 -6 0	0 6 -2	M16
3 -3 1	2 2 0	0 2 6	2 0 -6	M17
-3 -3 -1	-2 2 0	0 2 -6	-2 0 6	M18
-1 3 3	0 2 -2	-6 0 -2	6 2 0	M19
-1 -3 -3	0 -2 2	-6 0 2	6 -2 0	M20
3 1 3	-2 0 2	0 -6 2	-2 6 0	M21
3 3 1	2 -2 0	2 0 -6	0 -2 6	M22
-3 -1 3	2 0 2	0 6 2	2 -6 0	M23
-3 3 -1	-2 -2 0	-2 0 6	0 -2 -6	M24
3 -1 -3	-2 0 -2	0 6 -2	-2 -6 0	M25
-3 -3 1	-2 2 0	-2 0 -6	0 2 6	M26
-3 1 -3	2 0 -2	0 -6 -2	2 6 0	M27
3 -3 -1	2 2 0	2 0 6	0 2 -6	M28
3 1 3	2 0 -2	0 6 -2	2 -6 0	M29
3 3 1	-2 2 0	-2 0 6	0 2 -6	M30
-3 -1 3	-2 0 -2	0 -6 -2	-2 6 0	M31
-3 3 -1	2 2 0	2 0 -6	0 2 6	M32
3 -1 -3	2 0 2	0 -6 2	2 6 0	M33
-3 -3 1	2 -2 0	2 0 6	0 -2 -6	M34
-3 1 -3	-2 0 2	0 6 2	-2 -6 0	M35
3 -3 -1	-2 -2 0	-2 0 -6	0 -2 6	M36
-3 1 3	-2 0 -2	-2 -6 0	0 6 -2	M37
3 -1 3	2 0 -2	2 6 0	0 -6 -2	M38
1 -3 3	0 2 2	-6 0 2	6 2 0	M39
1 3 -3	0 -2 -2	-6 0 -2	6 -2 0	M40
3 3 -1	-2 2 0	0 2 6	-2 0 -6	M41
-3 3 1	2 2 0	0 2 -6	2 0 6	M42
-3 1 3	2 0 2	2 6 0	0 -6 2	M43
3 -1 3	-2 0 2	-2 -6 0	0 6 2	M44
1 -3 3	0 -2 -2	6 0 -2	-6 -2 0	M45
1 3 -3	0 2 2	6 0 2	-6 2 0	M46
3 3 -1	2 -2 0	0 -2 -6	2 0 6	M47
-3 3 1	-2 -2 0	0 -2 6	-2 0 -6	M48

Table 43: P - lattice [2 3 3]

[*uvw*]	*hkl A*	*hkl B*	*hkl C*	M No.
2 3 3	0 1 -1	-3 1 1	3 0 -2	M1
2 3 3	0 -1 1	3 -1 -1	-3 0 2	M2
2 -3 -3	0 -1 1	-3 -1 -1	3 0 2	M3
-2 3 -3	0 1 1	3 1 -1	-3 0 2	M4
-2 -3 3	0 -1 -1	3 -1 1	-3 0 -2	M5
3 2 -3	1 0 1	1 -3 -1	0 3 2	M6
-3 -2 -3	-1 0 1	-1 3 -1	0 -3 2	M7
3 -3 2	-1 -1 0	1 -1 -3	-2 0 3	M8
-3 -3 -2	1 -1 0	-1 -1 3	2 0 -3	M9
-2 3 3	0 -1 1	3 1 1	-3 -2 0	M10
-2 -3 -3	0 1 -1	3 -1 -1	-3 2 0	M11
2 -3 -3	0 1 -1	3 1 1	-3 0 -2	M12
-2 3 -3	0 -1 -1	-3 -1 1	3 0 -2	M13
-2 -3 3	0 1 1	-3 1 -1	3 0 2	M14
3 2 -3	-1 0 -1	-1 3 1	0 -3 -2	M15
-3 -2 -3	1 0 -1	1 -3 1	0 3 -2	M16
3 -3 2	1 1 0	-1 1 3	2 0 -3	M17
-3 -3 -2	-1 1 0	1 1 -3	-2 0 3	M18
-2 3 3	0 1 -1	-3 -1 -1	3 2 0	M19
-2 -3 -3	0 -1 1	-3 1 1	3 -2 0	M20
3 2 3	-1 0 1	1 -3 1	-2 3 0	M21
3 3 2	1 -1 0	1 1 -3	0 -2 3	M22
-3 -2 3	1 0 1	-1 3 1	2 -3 0	M23
-3 3 -2	-1 -1 0	-1 1 3	0 -2 -3	M24
3 -2 -3	-1 0 -1	1 3 -1	-2 -3 0	M25
-3 -3 2	-1 1 0	-1 -1 -3	0 2 3	M26
-3 2 -3	1 0 -1	-1 -3 -1	2 3 0	M27
3 -3 -2	1 1 0	1 -1 3	0 2 -3	M28
3 2 3	1 0 -1	-1 3 -1	2 -3 0	M29
3 3 2	-1 1 0	-1 -1 3	0 2 -3	M30
-3 -2 3	-1 0 -1	1 -3 -1	-2 3 0	M31
-3 3 -2	1 1 0	1 -1 -3	0 2 3	M32
3 -2 -3	1 0 1	-1 -3 1	2 3 0	M33
-3 -3 2	1 -1 0	1 1 3	0 -2 -3	M34
-3 2 -3	-1 0 1	1 3 1	-2 -3 0	M35
3 -3 -2	-1 -1 0	-1 1 -3	0 -2 3	M36
-3 2 3	-1 0 -1	-1 -3 1	0 3 -2	M37
3 -2 3	1 0 -1	1 3 1	0 -3 -2	M38
2 -3 3	0 1 1	-3 -1 1	3 2 0	M39
2 3 -3	0 -1 -1	-3 1 -1	3 -2 0	M40
3 3 -2	-1 1 0	1 1 3	-2 0 -3	M41
-3 3 2	1 1 0	-1 1 -3	2 0 3	M42
-3 2 3	1 0 1	1 3 -1	0 -3 2	M43
3 -2 3	-1 0 1	-1 -3 -1	0 3 2	M44
2 -3 3	0 -1 -1	3 1 -1	-3 -2 0	M45
2 3 -3	0 1 1	3 -1 1	-3 2 0	M46
3 3 -2	1 -1 0	-1 -1 -3	2 0 3	M47
-3 3 2	-1 -1 0	1 -1 3	-2 0 -3	M48

Table 44: I - lattice [2 3 3]

[uvw]	hkl A	hkl B	hkl C	M No.
2 3 3	0 1 -1	-6 2 2	6 -1 -3	M1
2 3 3	0 -1 1	6 -2 -2	-6 1 3	M2
2 -3 -3	0 -1 1	-6 -2 -2	6 1 3	M3
-2 3 -3	0 1 1	6 2 -2	-6 -1 3	M4
-2 -3 3	0 -1 -1	6 -2 2	-6 1 -3	M5
3 2 -3	1 0 1	2 -6 -2	-1 6 3	M6
-3 -2 -3	-1 0 1	-2 6 -2	1 -6 3	M7
3 -3 2	-1 -1 0	2 -2 -6	-3 1 6	M8
-3 -3 -2	1 -1 0	-2 -2 6	3 1 -6	M9
-2 3 3	0 -1 1	6 2 2	-6 -3 -1	M10
-2 -3 -3	0 1 -1	6 -2 -2	-6 3 1	M11
2 -3 -3	0 1 -1	6 2 2	-6 -1 -3	M12
-2 3 -3	0 -1 -1	-6 -2 2	6 1 -3	M13
-2 -3 3	0 1 1	-6 2 -2	6 -1 3	M14
3 2 -3	-1 0 -1	-2 6 2	1 -6 -3	M15
-3 -2 -3	1 0 -1	2 -6 2	-1 6 -3	M16
3 -3 2	1 1 0	-2 2 6	3 -1 -6	M17
-3 -3 -2	-1 1 0	2 2 -6	-3 -1 6	M18
-2 3 3	0 1 -1	-6 -2 -2	6 3 1	M19
-2 -3 -3	0 -1 1	-6 2 2	6 -3 -1	M20
3 2 3	-1 0 1	2 -6 2	-3 6 -1	M21
3 3 2	1 -1 0	2 2 -6	-1 -3 6	M22
-3 -2 3	1 0 1	-2 6 2	3 -6 -1	M23
-3 3 -2	-1 -1 0	-2 2 6	1 -3 -6	M24
3 -2 -3	-1 0 -1	2 6 -2	-3 -6 1	M25
-3 -3 2	-1 1 0	-2 -2 -6	1 3 6	M26
-3 2 -3	1 0 -1	-2 -6 -2	3 6 1	M27
3 -3 -2	1 1 0	2 -2 6	-1 3 -6	M28
3 2 3	1 0 -1	-2 6 -2	3 -6 1	M29
3 3 2	-1 1 0	-2 -2 6	1 3 -6	M30
-3 -2 3	-1 0 -1	2 -6 -2	-3 6 1	M31
-3 3 -2	1 1 0	2 -2 -6	-1 3 6	M32
3 -2 -3	1 0 1	-2 -6 2	3 6 -1	M33
-3 -3 2	1 -1 0	2 2 6	-1 -3 -6	M34
-3 2 -3	-1 0 1	2 6 2	-3 -6 -1	M35
3 -3 -2	-1 -1 0	-2 2 -6	1 -3 6	M36
-3 2 3	-1 0 -1	-2 -6 2	1 6 -3	M37
3 -2 3	1 0 -1	2 6 2	-1 -6 -3	M38
2 -3 3	0 1 1	-6 -2 2	6 3 -1	M39
2 3 -3	0 -1 -1	-6 2 -2	6 -3 1	M40
3 3 -2	-1 1 0	2 2 6	-3 -1 -6	M41
-3 3 2	1 1 0	-2 2 -6	3 -1 6	M42
-3 2 3	1 0 1	2 6 -2	-1 -6 3	M43
3 -2 3	-1 0 1	-2 -6 -2	1 6 3	M44
2 -3 3	0 -1 -1	6 2 -2	-6 -3 1	M45
2 3 -3	0 1 1	6 -2 2	-6 3 -1	M46
3 3 -2	1 -1 0	-2 -2 -6	3 1 6	M47
-3 3 2	-1 -1 0	2 -2 6	-3 1 -6	M48

Table 45: F - lattice [2 3 3]

[*uvw*]	*hkl A*	*hkl B*	*hkl C*	M No.
2 3 3	0 2 -2	-3 1 1	3 1 -3	M1
2 3 3	0 -2 2	3 -1 -1	-3 -1 3	M2
2 -3 -3	0 -2 2	-3 -1 -1	3 -1 3	M3
-2 3 -3	0 2 2	3 1 -1	-3 1 3	M4
-2 -3 3	0 -2 -2	3 -1 1	-3 -1 -3	M5
3 2 -3	2 0 2	1 -3 -1	1 3 3	M6
-3 -2 -3	-2 0 2	-1 3 -1	-1 -3 3	M7
3 -3 2	-2 -2 0	1 -1 -3	-3 -1 3	M8
-3 -3 -2	2 -2 0	-1 -1 3	3 -1 -3	M9
-2 3 3	0 -2 2	3 1 1	-3 -3 1	M10
-2 -3 -3	0 2 -2	3 -1 -1	-3 3 -1	M11
2 -3 -3	0 2 -2	3 1 1	-3 1 -3	M12
-2 3 -3	0 -2 -2	-3 -1 1	3 -1 -3	M13
-2 -3 3	0 2 2	-3 1 -1	3 1 3	M14
3 2 -3	-2 0 -2	-1 3 1	-1 -3 -3	M15
-3 -2 -3	2 0 -2	1 -3 1	1 3 -3	M16
3 -3 2	2 2 0	-1 1 3	3 1 -3	M17
-3 -3 -2	-2 2 0	1 1 -3	-3 1 3	M18
-2 3 3	0 2 -2	-3 -1 -1	3 3 -1	M19
-2 -3 -3	0 -2 2	-3 1 1	3 -3 1	M20
3 2 3	-2 0 2	1 -3 1	-3 3 1	M21
3 3 2	2 -2 0	1 1 -3	1 -3 3	M22
-3 -2 3	2 0 2	-1 3 1	3 -3 1	M23
-3 3 -2	-2 -2 0	-1 1 3	-1 -3 -3	M24
3 -2 -3	-2 0 -2	1 3 -1	-3 -3 -1	M25
-3 -3 2	-2 2 0	-1 -1 -3	-1 3 3	M26
-3 2 -3	2 0 -2	-1 -3 -1	3 3 -1	M27
3 -3 -2	2 2 0	1 -1 3	1 3 -3	M28
3 2 3	2 0 -2	-1 3 -1	3 -3 -1	M29
3 3 2	-2 2 0	-1 -1 3	-1 3 -3	M30
-3 -2 3	-2 0 -2	1 -3 -1	-3 3 -1	M31
-3 3 -2	2 2 0	1 -1 -3	1 3 3	M32
3 -2 -3	2 0 2	-1 -3 1	3 3 1	M33
-3 -3 2	2 -2 0	1 1 3	1 -3 -3	M34
-3 2 -3	-2 0 2	1 3 1	-3 -3 1	M35
3 -3 -2	-2 -2 0	-1 1 -3	-1 -3 3	M36
-3 2 3	-2 0 -2	-1 -3 1	-1 3 -3	M37
3 -2 3	2 0 -2	1 3 1	1 -3 -3	M38
2 -3 3	0 2 2	-3 -1 1	3 3 1	M39
2 3 -3	0 -2 -2	-3 1 -1	3 -3 -1	M40
3 3 -2	-2 2 0	1 1 3	-3 1 -3	M41
-3 3 2	2 2 0	-1 1 -3	3 1 3	M42
-3 2 3	2 0 2	1 3 -1	1 -3 3	M43
3 -2 3	-2 0 2	-1 -3 -1	-1 3 3	M44
2 -3 3	0 -2 -2	3 1 -1	-3 -3 -1	M45
2 3 -3	0 2 2	3 -1 1	-3 3 1	M46
3 3 -2	2 -2 0	-1 -1 -3	3 -1 3	M47
-3 3 2	-2 -2 0	1 -1 3	-3 -1 -3	M48
